# On the Number of Neurons and Time Scale of Integration Underlying the Formation of Percepts in the Brain

**DOI:** 10.1371/journal.pcbi.1004082

**Published:** 2015-03-20

**Authors:** Adrien Wohrer, Christian K. Machens

**Affiliations:** 1 Group for Neural Theory, Laboratoire de Neurosciences Cognitives, INSERM U960, École Normale Supérieure, Paris, France; 2 Champalimaud Neuroscience Programme, Champalimaud Centre for the Unknown, Lisbon, Portugal; The University of Texas at Austin, UNITED STATES

## Abstract

All of our perceptual experiences arise from the activity of neural populations. Here we study the formation of such percepts under the assumption that they emerge from a linear readout, i.e., a weighted sum of the neurons’ firing rates. We show that this assumption constrains the trial-to-trial covariance structure of neural activities and animal behavior. The predicted covariance structure depends on the readout parameters, and in particular on the temporal integration window *w* and typical number of neurons *K* used in the formation of the percept. Using these predictions, we show how to infer the readout parameters from joint measurements of a subject’s behavior and neural activities. We consider three such scenarios: (1) recordings from the complete neural population, (2) recordings of neuronal sub-ensembles whose size exceeds *K*, and (3) recordings of neuronal sub-ensembles that are smaller than *K*. Using theoretical arguments and artificially generated data, we show that the first two scenarios allow us to recover the typical spatial and temporal scales of the readout. In the third scenario, we show that the readout parameters can only be recovered by making additional assumptions about the structure of the full population activity. Our work provides the first thorough interpretation of (feed-forward) percept formation from a population of sensory neurons. We discuss applications to experimental recordings in classic sensory decision-making tasks, which will hopefully provide new insights into the nature of perceptual integration.

## Author Summary

This article deals with the interpretation of neural activities during perceptual decision-making tasks, where animals must assess the value of a sensory stimulus and take a decision on the basis of their percept. A “standard model” for these tasks has progressively emerged, whence the animal’s percept and subsequent choice on each trial are obtained from a linear integration of the activity of sensory neurons. However, up to date, there has been no principled method to estimate the parameters of this model: mainly, the typical number of neurons *K* from the population involved in conveying the percept, and the typical time scale *w* during which these neurons’ activities are integrated. In this article, we propose a novel method to estimate these quantities from experimental data, and thus assess the validity of the standard model of percept formation. In the process, we clarify the predictions of the standard model regarding two classic experimental measures in these tasks: *sensitivity*, which is the animal’s ability to distinguish nearby stimulus values, and *choice signals*, which assess the amount of correlation between the activity of single neurons and the animal’s ultimate choice on each trial.

## Introduction

Most cortical neurons are noisy, or at least appear so in experiments. When we record the responses of sensory neurons to well-controlled stimuli, their spike patterns vary from trial to trial. Does this variability reflect the uncertainties of the measurement process, or does it have a direct impact on behavior? These questions are central to our understanding of percept formation and decision-making in the brain and have been the focus of much previous work [1]. Many studies have sought to address these problems by studying animals that perform simple, perceptual decision-making tasks [2, 3]. In such tasks, an animal is typically presented with different stimuli *s* and trained to categorize them through a simple behavioral report. When this perceptual report is monitored simultaneously with the animal’s neural activity, one can try to find a causal link between the two.

One particular hypothesis about this link—which we refer to as the “sensory noise” hypothesis—postulates that the accuracy of the animal’s perceptual judgments is primarily limited by noise at the level of sensory neurons [4, 5]. In terms of signal detection theory, the hypothesis predicts a quantitative match between (1) the animal’s ability to discriminate nearby stimulus values—known as *psychometric* sensitivity, and (2) an ideal observer’s ability to discriminate nearby stimulus values based on the activities of the underlying neural population—known as *neurometric* sensitivity. Both types of sensitivities can be quantified as signal-to-noise ratios (SNR). With this idea in mind, several studies have compared the neurometric and psychometric sensitivities in various sensory systems and behavioral tasks (see [6, 7] for reference).

However, as was soon realized, any extrapolation from a few recorded cells to the entire population is fraught with implicit assumptions. For example, if neurons in a population behave independently one from another, then the SNR of the population is simply the sum of the individual SNRs. Consequently, any estimate of neurometric sensitivity will grow linearly with the number of recorded neurons *K*. However, if neurons in a population do not behave independently, the precise growth of neural sensitivity with *K* is determined by the correlation structure of noise in the population [8–10]. In addition, the neurometric sensitivities also depend on the time scale *w* that is used to integrate each neuron’s spike train in a given trial [3, 11–13]. Indeed, the more spikes are incorporated in the readout, the more accurate that readout will be. Adding extra neurons by increasing *K*, or adding extra spikes by increasing *w*, are two dual ways of increasing the readout’s overall SNR.

As there is no unique way of reading out information from a population of sensory neurons, the sensory noise hypothesis can only be tested if we understand how the organism itself “reads out” the relevant information. In other words, how many sensory neurons *K*, and what integration time scale *w*, provide a relevant description of the animal’s percept formation? Given the “*K*-*w*” duality mentioned above, we cannot answer that question based solely on sensitivity (SNR). Another experimental measure should also be included in the analysis.

A good candidate for such a measure are *choice signals*, i.e., measures of the trial-to-trial correlation between the activity of each recorded neuron and the animal’s ultimate choice on each trial. These signals, weak but often significant, arise from the unknown process by which each neuron’s activity influences—or is influenced by—the animal’s perceptual decision. In two-alternative forced choice (2AFC) discrimination tasks, they have generally been computed in the form of *choice probabilities* (CP) [14, 15]. The temporal evolution of CPs has been used to find the instants in time when a given population covaries with the animal’s percept [13, 16]. In a seminal study, Shadlen et al. (1996) proposed to jointly use sensitivity and choice signals, as two independent constraints characterizing the underlying neural code [17]. They derived a feed-forward model of perceptual integration in visual area MT, and studied numerically how the population’s sensitivity and CPs vary as a function of various model parameters. They acknowledged the existence of a link between CPs and pairwise noise correlations—both measures being (partial) reflections of how information is embedded in the neural population as a whole (see also [12, 18]). However, the quantitative nature of this link was only revealed recently, when Haefner et al. (2013) derived the analytical expression of CPs in the standard model of perceptual integration [19] (see Methods).

In this article, we show that the standard feed-forward model of percept formation gives rise to three *characteristic equations* that describe analytically the trial-to-trial covariance between neural activities and animal percept. These equations depend on the brain’s readout policy across neurons and time, and hold for any noise correlation structure in the neural population. In accordance with the intuition of Shadlen et al. (1996), we show that sensitivity and choice signals correspond to two distinct, characteristic properties of the readout. The equation describing choice signals is equivalent to the one derived by Haefner et al. (2013), but stripped from the non-linear complications inherent to the CP formulation. We use a linear formulation instead, which gives us a particularly simple prediction of choice signals at every instant in time.

We then show how these equations can be used in order to recover the time window and the number of neurons used in the formation of a percept. A quantitative analysis of choice signals allows us to overcome the “*K*–*w* trade-off” inherent to neurometric sensitivity. We specifically focus on situations in which only a finite sample of neurons has been measured from a large, unknown population. We show how to recover the typical number of neurons *K*, provided that the experimenter could record at least *K* neurons simultaneously. Finally, we discuss the scope and the limitations of our method, and how it can be applied to real experimental data.

## Results

### Experimental measures of behavior and neural activities

**Fig 1:**
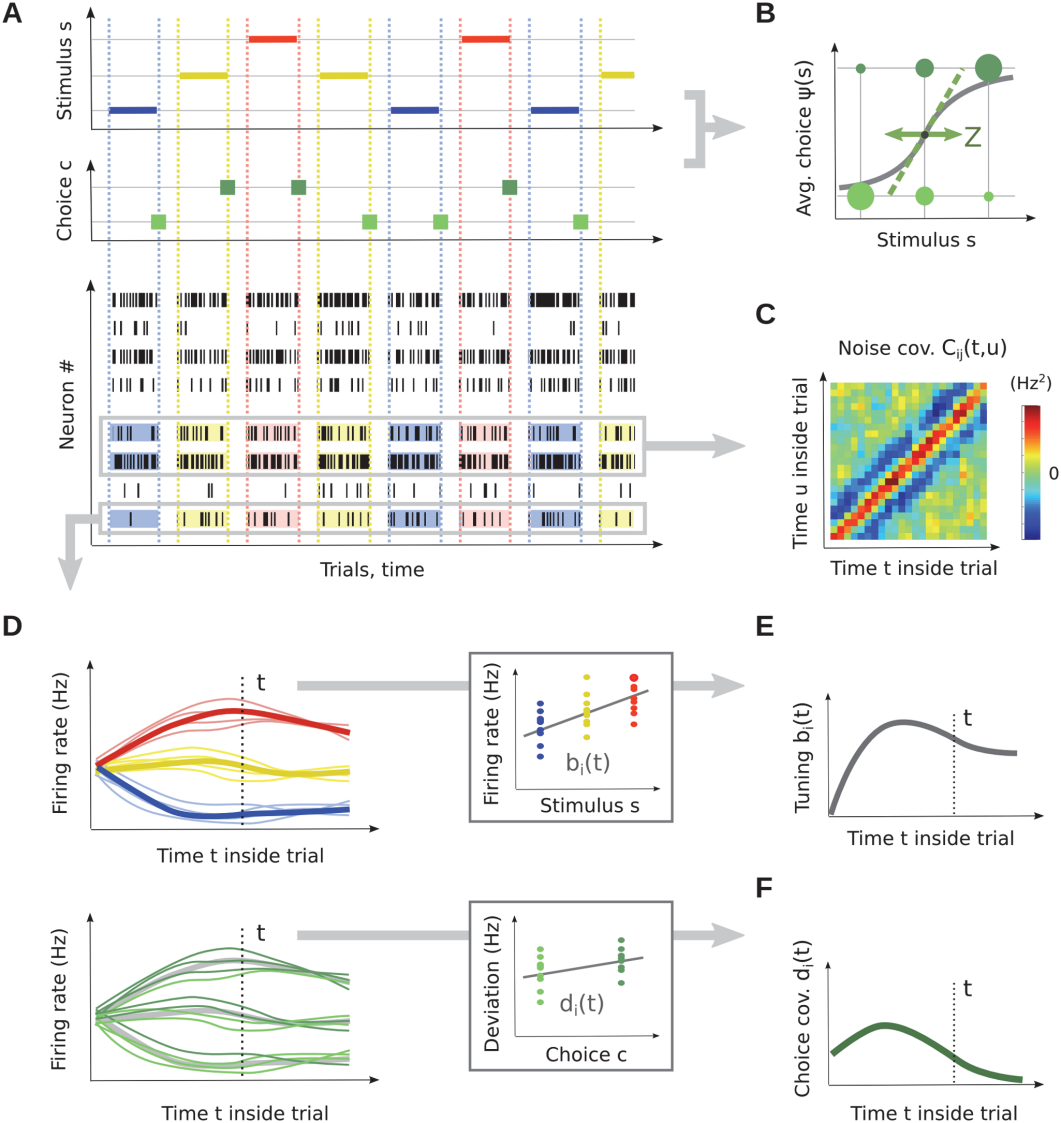
Framework and main experimental measures. (A) Experimental setup. Top: A set of stimulus values *s* (color-coded as blue, yellow, red) are repeatedly presented to an animal. Middle: The animal’s choice *c* on each trial (green) indicates whether the animal judged *s* to be larger or smaller than the fixed central value, *s*_0_. Bottom: In each session, several task-relevant sensory neurons are recorded simultaneously with the behavior. (B) The psychometric curve *ψ*(*s*) quantifies the animal’s sensory accuracy. Its inverse slope in *s*_0_ provides the just-noticeable-difference (JND), *Z*. (C) The noise covariance structure can be assessed in each pair of simultaneously recorded neurons, as their joint peri-stimulus histogram (JPSTH) *C*_*ij*_(*t*, *u*). (D) Responses of a particular neuron. Each thin line is the schematic (smoothed) representation of the spike train on one trial. Segregating trials according to stimulus (top), we access the neuron’s peri-stimulus histogram (PSTH, thick lines) and its tuning signal *b*_*i*_(*t*)—shown in panel (E). Fixing a stimulus value and segregating trials according to the animal’s choice *c* (bottom), we access the neuron’s choice covariance (CC) curve *d*_*i*_(*t*)—shown in panel (F).

We will study the formation of percepts in the context of perceptual decision-making experiments (Fig. 1, see Methods or Tables 1–3 for the corresponding formulas). In these experiments, an animal is typically confronted with a stimulus, *s*, and must then make a behavioral choice, *c*, according to the rules of the task. A specific example is the classic *discrimination task* in which the animal’s choice *c* is binary, and the animal must report whether it perceived *s* to be higher (*c* = 1) or lower (*c* = 0) than a fixed reference *s*_0_ (Fig. 1A, top and middle panels). While the animal is performing the task, the neural activity in a given brain area can be monitored (Fig. 1A, bottom panel). Typical examples from the literature include area MT in the context of a motion discrimination task [3], area MT or V2 in the context of a depth discrimination task [11, 20], or area S1 in the context of a tactile discrimination task [21]. For concreteness, we will mostly focus on these discrimination tasks, although the general framework can be applied to arbitrary perceptual decision-making tasks.

**Table 1:** Variables and notations: typography.

**Notation**	**Description**	**Examples**
Bold	Lower case: vector notation, across neurons Upper case: matrix notation, across neurons	**r**(*t*), **b**(*t*) **C**(*t*, *u*), $\bar{\bar{\text{C}}}$
Overline	Temporal integration, using readout parameters (*w*, *t*_*R*_)	${\bar{r}}_{i}$, ${\bar{\bar{C}}}_{ij}$, $\bar{\bar{q}}$
Starred	Pertaining to the animal’s true behavior (as opposed to model-based predictions)	*Z* ^⋆^, $d_{i}^{⋆}(t)$, $t_{R}^{⋆}$
E[⋅]	Expectation across trials (can also be conditional)	E[*s*], E[*r*_*i*_(*t*)∣*s*], E[*sr*_*i*_(*t*)]
Cov[⋅]	Covariance across trials (can also be conditional)	Cov[*r*_*i*_(*t*), *c* ^⋆^∣*s*]
**x**_*r*_	Vector (or matrix) **x** restricted to neurons in readout ensemble 𝓔	**a**_*r*_, ${\bar{\text{b}}}_{r}$, ${\bar{\bar{\text{C}}}}_{r}$, ${\bar{\text{C}}}_{ir}(t)$

**Table 2:** Variables and notations: experimental data.

**Raw experimental data**			**Ref in text**
*s*	Stimulus—a varying scalar value on each trial		
*s*_0_	Threshold stimulus value in the 2AFC task		
*c* ^⋆^	Animal choice—binary report on each trial		
*r*_*i*_(*t*)	Spike train from neuron *i* in a given trial		
$\sigma_{s}^{2}$	Stimulus variance across trials	$\sigma_{s}^{2}≔\text{Var}[s]$	after eq. 18
**Animal psychometry**			
*ψ* ^⋆^(*s*)	Psychometric curve	*ψ* ^⋆^(*s*)≔E[*c* ^⋆^∣*s*]	eq. 23
*Z* ^⋆^ $\mu_{d}^{⋆}$	Just-noticeable difference Decision bias	Best fit to $\psi^{⋆}(s)=\Phi (\frac{s+\mu_{d}^{⋆}-s_{0}}{Z^{⋆}})$	eq. 27
**Individual statistics for the neurons**			
*m*_*i*_(*t*; *s*)	PSTH for neuron *i* with stimulus *s*	*m*_*i*_(*t*; *s*)≔E[*r*_*i*_(*t*)∣*s*]	eq. 24
*b*_*i*_(*t*)	Tuning signal for neuron *i* (variation of the PSTH wrt. stimulus)	$b_{i}(t)≔\partial_{s}^{}\;m_{i}(t;s)$	eq. 30, 31
*C*_*ij*_(*t*, *u*)	JPSTH for neurons *i* and *j* (pairwise noise correlations)	$C_{ij}(t,u)≔\text{E}[\text{Cov}[r_{i}(t),r_{j}(u)|s]]$	eq. 25, 32
$\bar{\bar{\text{C}}}$	Noise covariance matrix (time average of *C*, with parameters (*w*, *t*_*R*_))	${\bar{\bar{C}}}_{ij}≔\text{E}[\text{Cov}[{\bar{r}}_{i},{\bar{r}}_{j}|s]]$	eq. 40
$d_{i}^{⋆}(t)$	Choice Covariance for neuron *i* (linear equivalent of choice probabilities)	$d_{i}^{⋆}(t)≔\text{E}[\text{Cov}[r_{i}(t),c^{⋆}|s]]$	eq. 26, 33

**Table 3:** Variables and notations: model and methods.

**Linear readout and decision model**			**Ref in text**
*w*	Readout window—duration of the temporal integration		
*t*_*R*_	extraction time—time at which the percept is formed		
*a*_*i*_	Readout weight—contribution of neuron *i* to the percept		
*σ*_*d*_	Decision noise—added to the percept at decision time		
$\hat{s}$	Readout—computed on every trial	$\hat{s}=a_{0}+\sum_{i=1}^{N_{\text{tot}}}a_{i}{\bar{r}}_{i}$	eq. 2
*c*	Choice—computed on every trial	$c=H(\hat{s}+\xi_{d}-s_{0})$	eq. 3
**Model predictions** (characteristic equations)			
*Z*	Just-noticeable difference	$Z^{2}={\text{a}}^{⊤}\bar{\bar{\text{C}}}\text{a}+\sigma_{d}^{2}$	eq. 8
**d**(*t*)	Choice covariance for every neuron	$\text{d}(t)=\kappa (Z)\;\bar{\text{C}}(t)\text{a}$	eq. 9
*κ*(*Z*)	Conversion factor from *Percept Covariance* to *Choice Covariance*		eq. 46
**Restricted optimality** hypothesis			
𝓔	Readout ensemble—neurons used for the readout		
*K*	Readout size—number of neurons in 𝓔		
**H**	Restriction matrix on 𝓔 (of size *K* × *N*_tot_)	${\text{x}}_{r}^{\;}=\text{Hx}$	eq. 54
**a**_*r*_	Optimal readout vector (over 𝓔)	${\text{a}}_{r}\sim {\left({\bar{\bar{\text{C}}}}_{r}\right)}^{-1}{\bar{\text{b}}}_{r}\;$	eq. 12
**Population-wide indicators for choice signals**			
*q*(*u*, *t*)	Population-wide link between tuning and CC	*q*(*u*, *t*)≔⟨*b*_*i*_(*u*)*d*_*i*_(*t*)⟩_*i*_	eq. 14
*V*	Deviation from linearity between tuning and CC	$V\;≔{\langle {\bar{b}}_{i}^{2}\rangle }_{i}^{}{\langle {\bar{d}}_{i}^{2}\rangle }_{i}^{}-\;{\bar{\bar{q}}}^{2}$	eq. 15
**Rescaled indicators** (used in the SVD analysis)			
**A**	Total covariance matrix	$\text{A}=\bar{\bar{\text{C}}}+\sigma_{s}^{2}\bar{\text{b}}{\bar{\text{b}}}^{⊤}$	eq. 50
*Y*	Sensitivity to stimulus	$Y≔\sigma_{s}^{2}\;/\;(\sigma_{s}^{2}+Z^{2})$	eq. 47
**e**	Total percept covariance	**e** ≔ **A** **a**	eq. 76
*Q*	Rescaled version of $\bar{\bar{q}}$	$Q\;≔\;{\left\langle e_{i}{\bar{b}}_{i}\right\rangle }_{i}$	eq. 18, 79
***η***	Tuning vector in the space of modes	$\bar{\text{b}}=\text{U}\text{Λ}\text{η}$	eq. 68

The animal’s behavior in a discrimination task can be quantified through the *psychometric curve* *ψ*(*s*). This curve measures the animal’s repartition of responses at each stimulus value *s* (Fig. 1B). If the animal is unbiased, it will choose randomly whenever the stimulus *s* is equal to the threshold value *s*_0_, so that *ψ*(*s*_0_) = 1/2. The slope of the psychometric curve at *s* = *s*_0_ determines the animal’s ability to distinguish near-threshold values of the stimulus, i.e., its psychometric sensitivity. We assess this sensitivity through the *just noticeable difference* (JND) or *difference limen*, noted *Z*. The more sensitive the animal, the smaller *Z*, and the steeper its psychometric curve.

We assume that the neural activity within the recorded brain area conveys the stimulus information that the animal uses to make its choice (Fig. 1A, bottom). We describe the activity of this neural population on every trial as a multivariate point process **r**(*t*) = {*r*_*i*_(*t*)}_*i* = 1…*N*_tot__, where each *r*_*i*_(*t*) is the spike train for neuron *i*, and *N*_tot_ denotes the full population size, a very large and unknown number. (The number of neurons actually recorded is generally much smaller.) As is common in electrophysiological recordings, we will quantify the raw spike trains by their first and second order statistics. First, neuron *i*’s trial-averaged activity in response to each tested stimulus *s* is given by the peri-stimulus time histogram (PSTH) or time-varying firing rate, *m*_*i*_(*t*; *s*) (Fig. 1D). In so-called “fine” discrimination tasks, the stimuli *s* display only moderate variations around the central value *s*_0_, so that the PSTH at each instant in time can often be approximated by a linear function of *s*: $m_{i}(t\;;s)≃m_{i}^{0}(t)+b_{i}(t)s$. The slope *b*_*i*_(*t*), defined at every instant in time, summarizes neuron *i*’s tuning properties (Fig. 1E). Second, we assume that several neurons can be recorded simultaneously, so that we can access samples from the trial-to-trial covariance structure of the population activity (Fig. 1C). For every pair of neurons (*i*, *j*) and instants in time (*t*, *u*), the joint peri-stimulus time histogram (JPSTH, [22]) *C*_*ij*_(*t*, *u*) summarizes the pairwise noise correlations between the two neurons (eq. 25). For simplicity, we furthermore assume that the JPSTHs do not depend on the exact stimulus value *s*.

Finally, we can measure a choice signal for each neuron, which captures the trial-to-trial covariation of neuron activity *r*_*i*_(*t*) with the animal’s choice (Fig. 1F). Traditionally, this signal is measured in the form of choice probability (CP) curves. We consider here a simpler linear equivalent, that we term *choice covariance* (CC) curves [3]. The CC curve for neuron *i*, denoted by *d*_*i*_(*t*), measures the difference in firing rate (at each instant in time) between trials where the animal chose *c* = 1 and trials where it chose *c* = 0—all experimental features (including stimulus value) being fixed.

Unlike many characterizations of neural activity that rely only on spike counts, our framework requires an explicit temporal description of neural activity through PSTHs, JPSTHs, and CC curves. Exact formulas for these statistical measures are provided in the Methods. By keeping track of time, we will be able to predict *when*, and *how long*, perceptual integration takes place in an organism.

### From the neural activities to the animal’s choice

#### Linear readout model

**Fig 2:**
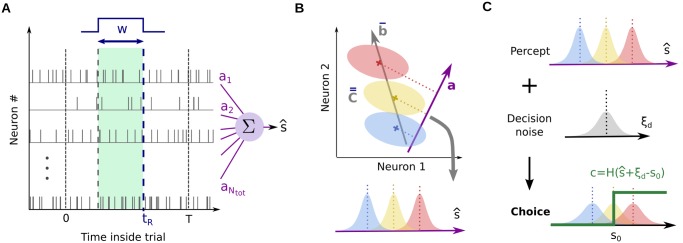
Linear readout and its interpretation. (A) We study a “standard” model of percept formation, with two parameters *w* and *t*_*R*_ defining integration in time, and a readout vector **a** defining integration across neurons. (B) Geometric interpretation of the model. The temporal parameters *w* and *t*_*R*_ define the tuning vector $\bar{\text{b}}$ and noise covariance matrix $\bar{\bar{\text{C}}}$ in the population. Colored ellipses represent the distribution of neural activities from trial to trial, for the three possible stimulus values. The readout $\hat{s}$ can be viewed as an orthogonal projection of neural activities in the direction given by **a**. (C) Behavioral part of the model. The percept $\hat{s}$ can be corrupted by decision noise *ξ*_*d*_. Then it is thresholded to produce a binary choice *c*.

Our goal is to quantify the mapping from the neural activities, **r**(*t*), to the animal’s choice, *c*. This can be done if we assume (1) how the stimulus information is extracted from the neural activities and (2) how the animal’s decision is formed. For (1) we assume the common linear readout model (Fig. 2A). Here, each neuron’s spike train *r*_*i*_(*t*) is first integrated into a single number describing the neuron’s activity over the trial. We write,

$$\begin{array}{c} {\bar{r}}_{i}=\frac{1}{w}\int_{t=0}^{t_{R}}\;dt\;h\left(\frac{t_{R}-t}{w}\right)\;r_{i}\left(t\right), \end{array}$$ (1)

where the kernel *h*(⋅) defines the shape of the integration window (e.g., square window, decreasing exponential, etc.), the parameter *w* controls the length of temporal integration, and the parameter *t*_*R*_ specifies the time at which the percept is built or read out. Second, the actual percept is given by a weighted sum over the neurons’ activities,

$$\begin{array}{c} \hat{s}=a_{0}+\sum_{i=1}^{N_{\mathrm{tot}}}a_{i}{\bar{r}}_{i}, \end{array}$$ (2)

where **a** = (*a*_1_, …, *a*_*N*_tot__) is a specific readout vector, or “perceptual policy”. This classic linear readout has sometimes been referred to as the “standard” model of perceptual integration [17, 19].

Previous studies have generally made ad hoc choices for the various constituents of this model. Most often, ${\bar{r}}_{i}$ is taken to be the total spike count for neuron *i*, in which case *t*_*R*_ = *w* coincides with the end of the stimulation period, and *h*(⋅) in eq. 1 is a square kernel. However, this readout is likely incorrect: the length of the integration window *w* influences the neurometric sensitivity, and experiments suggest that animals do not always use the full stimulation period to build their judgment [23]. Similarly, vector **a** is often defined over an arbitrary set of neurons, typically those recorded by the experimenter. Again, this choice is arbitrary, and it has a direct influence on the predicted sensitivities.

Instead, we view the readout window *w* and extraction time *t*_*R*_ as free parameters, and we generically define **a** over the full, unknown population of neurons. If a neuron does not contribute to the percept, it simply corresponds to a zero entry in **a**. For conceptual and implementation simplicity, we take *h*(⋅) to be a simple square window (see Discussion for a generalization). Our goal is now to understand whether the readout $\hat{s}$ can be a good model for the animal’s true percept formation and if yes, for what set of parameters.

#### Decision policy

The linear model builds a continuous-valued, internal percept $\hat{s}$ of stimulus value by the animal on each trial. To emulate the discrimination tasks, we also need to model the animal’s decision policy, which converts the continuous percept $\hat{s}$ into a binary choice *c*. While the linear model is rather universal, the decision model will depend on the specifics of each experimental task. To ground our argumentation, we model here the required decision in a classic random dot motion discrimination task [3]. However, the ideas herein could also be transposed to other types of behavioral tasks (see Discussion).

On each trial, we assume that an extraneous source of noise *ξ*_*d*_ is added to the animal’s percept $\hat{s}$ (Fig. 2C). Known in the literature as ‘decision noise’ or ‘pooling noise’, *ξ*_*d*_ encompasses all extra-sensory sources of variation which may influence the animal’s decision. We assume that *ξ*_*d*_ is a Gaussian variable with variance $\sigma_{d}^{2}$, which we take as an additional model parameter. Then, the animal’s choice on each trial is built deterministically, by comparing $\hat{s}+\xi_{d}$ to the threshold value *s*_0_ (Fig. 2C), so that

$$\begin{array}{c} c=H(\hat{s}+\xi_{d}-s_{0}), \end{array}$$ (3)

where *H*(⋅) is the Heaviside function. We note that the decision noise is negligible in the classic “sensory noise hypothesis”, in which case *σ*_*d*_ → 0.

### The characteristic equations of the standard model

The linear readout model and the animal’s decision policy specify both how the animal’s percepts are formed from its neural activities and how its choices are generated from these percepts. If we had recorded the activities of the entire neural population together with the animal’s behavior, then the parameters of this model could be estimated from the data using any standard regression method. However, this is generally not a realistic experimental situation. Instead, we take here a statistical approach to the problem, which (1) allows us to deal with incomplete recordings and (2) relates the estimation problem to the standard experimental measures described above.

#### Characteristic equations of the linear readout

Thanks to its linear structure, the readout defined in eq. 2 induces a simple covariance between the neural activities, **r**(*t*), and the resulting percept, $\hat{s}$ (Fig. 2B). Since the linear readout relies on the integrated spike trains, eq. 1, we need similarly integrated versions of the neural tuning and noise covariances in order to express the respective covariance relations. In general, we will denote these time-integrated quantities by an overhead bar, and alert the reader that the respective quantities depend implicitly on the readout window *w* and the extraction time *t*_*R*_. We will write ${\bar{b}}_{i}$ for the integrated version of the neural tuning, *b*_*i*_(*t*), we will write ${\bar{C}}_{ij}(t)$ for the once integrated noise covariances, and ${\bar{\bar{C}}}_{ij}$ for the doubly integrated noise covariance. This latter quantity, known in the literature as the ‘noise covariance matrix’, measures how the spike counts of two neurons, ${\bar{r}}_{i}$ and ${\bar{r}}_{j}$, covary due to shared random fluctuations across trials (stimulus *s* being held fixed). We can then summarize the covariances between neural activities and the resulting percepts by three characteristic equations (see Methods):

$$\begin{array}{c} \partial_{s}E\left[\hat{s}|s\right]={\bar{b}}^{⊤}a\;, \end{array}$$ (4)

$$\begin{array}{c} \mathrm{Var}[\hat{s}|s]=a^{⊤}\bar{\bar{C}}a\;, \end{array}$$ (5)

$$\begin{array}{c} \mathrm{Cov}[r\left(t\right),\hat{s}|s]=\bar{C}\left(t\right)a\;. \end{array}$$ (6)

On the left-hand sides of eq. 4–6, we find statistical quantities related to the percept $\hat{s}$. On the right-hand sides of these equations, we find the model’s predictions, which are based on the neurons’ (measurable) response statistics, *b* and *C*. More specifically, the first line describes the average dependency of $\hat{s}$ on stimulus *s*, the second line expresses the resulting variance for the percept, and the third line expresses the linear covariance between each neuron’s spike train, and the animal’s percept $\hat{s}$ on the trial.

#### Characteristic equations of the decision policy

To produce a binary choice, the continuous percept $\hat{s}$ is fed into the decision model (Fig. 2C). From the output of this decision model, we obtain a second set of characteristic equations (see Methods),

$$\begin{array}{c} 1=\partial_{s}E\left[\hat{s}|s\right]\;, \end{array}$$

$$\begin{array}{c} Z^{2}=\mathrm{Var}\left[\hat{s}|s\right]+\sigma_{d}^{2}\;, \end{array}$$

$$\begin{array}{c} d(t)=\kappa \left(Z\right)\;\mathrm{Cov}[r\left(t\right),\hat{s}|s]\;. \end{array}$$

Here the first equation simply expresses that both percept and decision are assumed to be unbiased. The second equation relates the JND, *Z*, extracted from the psychometric curve, to the variance in the percept, $\hat{s}$. The third equation restates the definition of choice covariance, except for the scaling factor, *κ*(*Z*), which will be constant for most practical purposes, and is described in detail in the Methods (eq. 46). Hence, in our full model of the task, we are able to predict both the psychometric sensitivity and the individual neurons’ choice signals from the first and second-order statistics of the neural responses. Specifically, by combining the characteristic equations for the linear readout and the decision policy, we obtain

$$\begin{array}{c} 1={\bar{b}}^{⊤}a\;, \end{array}$$ (7)

$$\begin{array}{c} Z^{2}=a^{⊤}\bar{\bar{C}}a+\sigma_{d}^{2}\;, \end{array}$$ (8)

$$\begin{array}{c} d(t)=\kappa \left(Z\right)\;\bar{C}\left(t\right)a\;. \end{array}$$ (9)

Importantly, since these equations deal with integrated versions of the raw neural signals, they depend on both the readout time window, *w*, and the extraction time, *t*_*R*_.

We note that the choice covariance equation (eq. 9) can also be derived in a simpler, time-averaged form. Let ${\bar{d}}_{i}$ be the time-integrated version of *d*_*i*_(*t*), using the readout’s temporal parameters (*w*, *t*_*R*_). Then, eq. 9 becomes

$$\begin{array}{c} \bar{d}=\kappa \left(Z\right)\;\bar{\bar{C}}a, \end{array}$$ (10)

which provides the linear covariance between each neuron’s spike count ${\bar{r}}_{i}$ on the trial, and the animal’s choice. This is essentially the relationship already revealed by Haefner et al. (2013) [19], that choice probabilities are related to readout weights through the noise covariance matrix. The simpler linear measure of choice covariance, used in this article, allows us (1) to get rid of some non-linearities inherent to the choice probability formulation, and (2) to easily extend the interpretation of choice signals in the time domain, with eq. 9.

### Estimating the parameters of sensory integration

Equations 7–9 describe the analytical link between measures of neural response to the stimulus (*b*_*i*_ and *C*_*ij*_) and measures related to the animal’s percept (*Z* and *d*_*i*_), based on the model’s readout parameters (**a**, *w*, *t*_*R*_, and *σ*_*d*_). This naturally raises the reverse question: can we estimate the parameters of the standard model (**a**, *w*, *t*_*R*_, and *σ*_*d*_) from actual measurements? From here on, we will denote the *true* (and unknown) values of these parameters, i.e., the values used in the animal’s actual percept formation, with a star (**a** ^⋆^, *w* ^⋆^, $t_{R}^{⋆}$, and $\sigma_{d}^{⋆}$).

**Fig 3:**
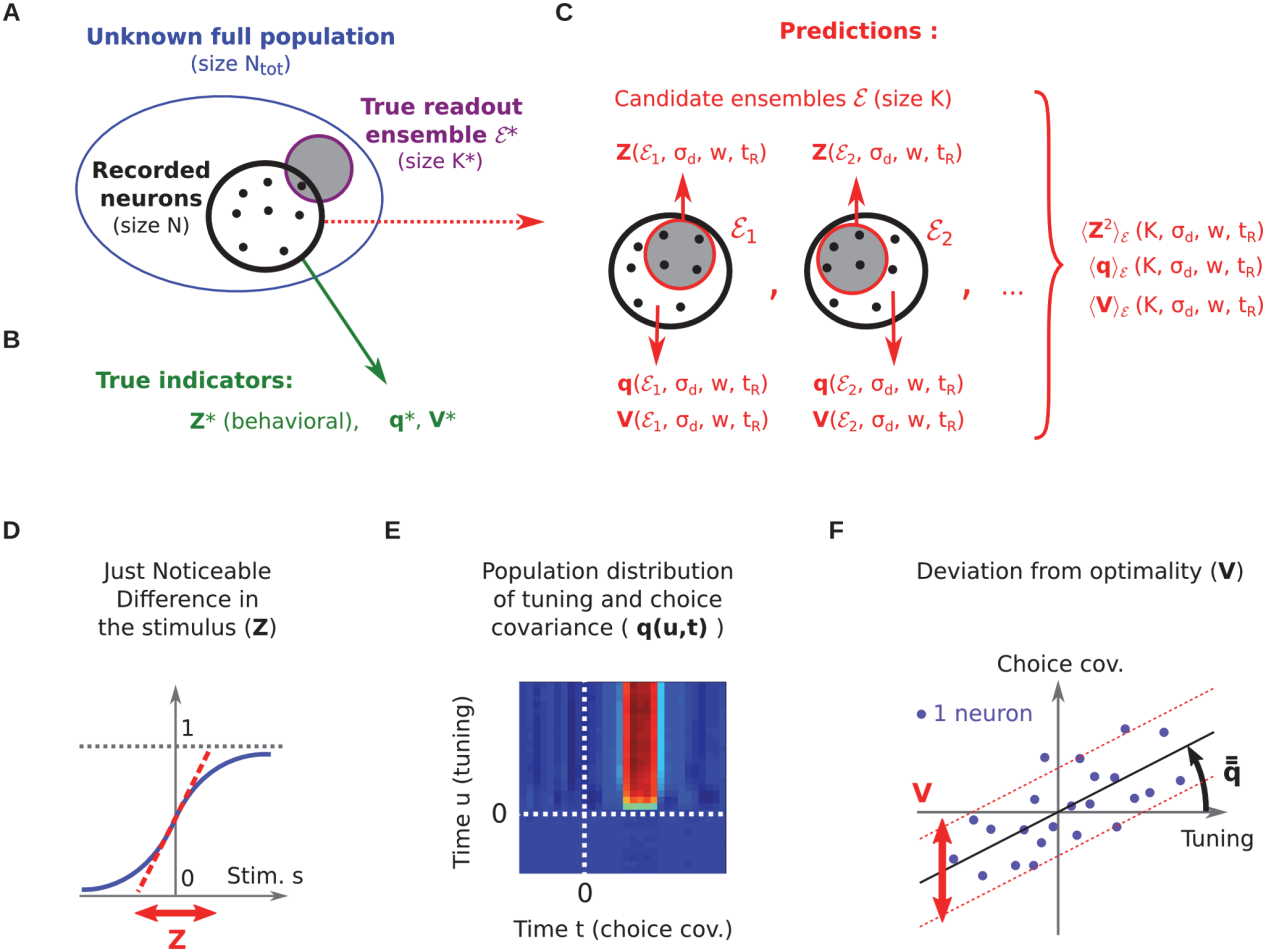
Statistical recovery of readout parameters: method. (A) The full population (of size *N*_tot_) and the true readout ensemble 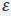 ^⋆^ (of size *K* ^⋆^), are not fully measured. Only subsets of *N* neurons are recorded simultaneously from the population. (B) True measures for the three statistical indicators: the animal’s psychometric JND *Z* ^⋆^, plus indicators *q* ^⋆^(*u*, *t*) and *V* ^⋆^ that summarize the distribution of the recorded neurons’ CC curves $d_{i}^{⋆}(t)$. (C) A large number of neural ensembles 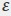 of size *K* are randomly selected from the experimental pool and proposed as the candidate readout ensemble. This yields model-based predictions for the indicators as a function of the proposed readout parameters (*K*, *σ*_*d*_, *w*, *t*_*R*_). (D-F) The three statistical indicators considered (see text for details).

As mentioned in the introduction, our primary interest concerns the trade-off between the time scale *w* ^⋆^ of integration, and the size *K* ^⋆^ of the functional population which conveys the animal’s percept to downstream areas. Thus, we assume that the animal’s percept is constructed from a specific sub-ensemble 𝓔^⋆^ of neurons, of size *K* ^⋆^ (Fig. 3A). Neurons inside 𝓔^⋆^ correspond to nonzero entries in the readout vector **a** ^⋆^, while neurons outside 𝓔^⋆^ have zero entries. Since only a subset of neurons within a cortical area will project to a downstream area, we can generally assume that *K* ^⋆^ < *N*_tot_.

Naturally, all those parameters are not measurable experimentally. For any candidate set of parameters, **a**, *w*, *t*_*R*_, and *σ*_*d*_, the characteristic equations 7–9 lead to *predictions* for *Z* and *d*_*i*_(*t*) (note the absence of star when referring to predictions). In turn, the experimenter *can* measure the animal’s actual choice *c* ^⋆^ on each trial, from which they can estimate the JND *Z* ^⋆^, and the CC curves $d_{i}^{⋆}(t)$ for all recorded neurons. In the next three sections, we study whether this information is sufficient to retrieve the true readout parameters, depending on the amount of data available.

In the ideal scenario where all neurons in the population are recorded simultaneously, *N* = *N*_tot_, all parameters can be retrieved exactly (Case 1). In most experimental recordings, however, we only measure the activities of a small subset of that population (Fig. 3A). If this subset is representative of the full population, we may want to retrieve the readout parameters through extrapolation. Unfortunately, any such extrapolation is fraught with additional assumptions—whether implicit or explicit—as it requires to replace the missing data with some form of (generative) model. In Case 2, we impose a generative model for the readout vector **a**. Coupled with a statistical principle, it allows us to estimate the true size *K* ^⋆^ of the readout ensemble, provided that the number of neurons recorded simultaneously, *N*, is larger: *N* > *K* ^⋆^. In Case 3, we study the scenario in which *N* ≤ *K* ^⋆^. Here, we need to assume a generative model for the neural activities themselves. Since the noise covariance structure assumed by that model exerts a strong influence on the predicted JND and CC curves, a direct inference of the readout scales becomes impossible.

### Case 1: all cells recorded

If all neurons in the population have been recorded, with a sufficient amount of trials to estimate the complete covariance structure of the population, then the only unknown quantities in eq. 7–9 are the readout parameters *w*, *t*_*R*_ and **a**, and the decision noise *σ*_*d*_. For fixed parameters *w* and *t*_*R*_, eq. 7 and 9 impose linear constraints on vector **a**. These constraints are generally over-complete, since **a** is *N*_tot_-dimensional, while each time *t* in eq. 9 provides *N*_tot_ additional linear constraints. Thus, in general, a solution **a** will only exist if one has targeted the true parameters *w* ^⋆^ and $t_{R}^{⋆}$, and it will then be unique. (If no choice of the readout parameters approximately fulfills the characteristic equations, we would have to conclude that the linear readout model is fundamentally wrong.) In practice, we can find the best solution to the characteristic equations by simply combining them and then minimizing the following mean-square error:

$$\begin{array}{c} L\left(w,t_{R},a,\sigma_{d}\right)={\left(1-{\bar{b}}^{⊤}a\right)}^{2}+\lambda {\left(Z^{⋆2}-\sigma_{d}^{2}-a^{⊤}\bar{\bar{C}}a\right)}^{2}+\mu \int dt{∥d^{*}\left(t\right)-\kappa \left(Z^{⋆}\right)\bar{C}\left(t\right)a∥}^{2}\;, \end{array}$$ (11)

where the parameters *λ* and *μ* trade off the importance of the errors in the different characteristic equations. Note that the loss function *L* depends not only on the readout weights **a** and the decision noise *σ*_*d*_, but also on the parameters *w* and *t*_*R*_, both of which enter all the time integrations that are denoted by an overhead bar. Once vector **a** ^⋆^ is estimated, the readout ensemble 𝓔^⋆^ will correspond to the set of neurons with nonzero readout weights.

### Case 2: more than *K* ^⋆^ cells recorded

Unfortunately, measuring the neural activity of a full population is essentially impossible, although optogenetic techniques are coming ever closer to this goal [24–26]. Nevertheless, if the activity patterns of the recorded cells are statistically similar to those of the readout ensemble, and if the number of simultaneously recorded cells exceeds the number of cells in the readout ensemble, we can still retrieve the readout parameters by making specific assumptions about the true readout vector **a** ^⋆^.

#### A statistical approach

Our central assumption will be that the system uses the principle of *restricted optimality*: we assume that the readout vector **a** ^⋆^ extracts as much information as possible from the neurons within the readout ensemble, 𝓔^⋆^, and no information from all other neurons. Since most of the neurons contributing to the readout were probably not recorded, we cannot directly estimate the true readout vector, **a** ^⋆^. However, we can form candidate ensembles from the recorded pool of neurons, 𝓔, compute their optimal readout vector, **a**_*r*_(𝓔), and then test to what extent these candidate ensembles can predict the JND or the CC curves (Fig. 3C). By changing the size of the candidate ensembles, *K*, we can in turn infer the number of neurons involved in the readout.

For an arbitrary candidate ensemble 𝓔, we can express its optimal readout vector, **a**_*r*_(𝓔) ≔ {*a*_*i*_}_*i* ∈ 𝓔_, on the basis of the neurons’ tuning and noise covariance, through a formula known as Fisher’s linear discriminant [27]:

$$\begin{array}{c} a_{r}=\frac{\;{\left({\bar{\bar{C}}}_{r}\right)}^{-1}{\bar{b}}_{r}^{\;}}{{\bar{b}}_{r}^{⊤}{\left({\bar{\bar{C}}}_{r}\right)}^{-1}{\bar{b}}_{r}^{\;}}. \end{array}$$ (12)

Here, the subscript *r* indicates that all quantities are only evaluated for the neurons within the ensemble 𝓔. The remaining neurons in the population do not participate in the readout. The resulting readout vector verifies eq. 7, and minimizes the just noticeable difference *Z* under the given constraints. Specifically, by entering the optimal readout into eq. 8, we obtain a prediction for the JND (Fig. 3D),

$$\begin{array}{c} Z^{2}=\frac{1}{{\bar{b}}_{r}^{⊤}{\left({\bar{\bar{C}}}_{r}\right)}^{-1}{\bar{b}}_{r}^{\;}}+\sigma_{d}^{2}\;. \end{array}$$ (13)

As for CC signals, the statistical description eliminates any reference to neuron identities, so we can no longer work directly with eq. 9. Instead, we re-express this equation in terms of two population-wide indicators, that summarize the CC signals of the individual neurons. The first indicator assesses the population-wide link between a neuron’s tuning at each time *u*, and its choice covariance at each time *t*. The second indicator measures the average deviation from this link (see also Methods):

$$\begin{array}{c} q(u,t)≔{\left\langle \;b_{i}\left(u\right)d_{i}\left(t\right)\;\right\rangle }_{i} \end{array}$$ (14)

$$\begin{array}{c} V≔{\left\langle {\bar{b}}_{i}^{2}\right\rangle }_{i}{\left\langle {\bar{d}}_{i}^{2}\right\rangle }_{i}-{\bar{\bar{q}}}^{2}. \end{array}$$ (15)

Here, the angular brackets ⟨⋅⟩_*i*_ denote averaging over the full neural population—or, in practice, over a representative ensemble of neurons (see Methods on how to construct this from actual data).

Experimentally, *q*(*u*, *t*) is expected to be globally positive, because the tuning of a neuron is often found to be somewhat correlated with its choice signal [11, 15] (Fig. 3E)—likely due to the fact that positively-tuned neurons contribute positively to stimulus estimation, and negatively-tuned neurons negatively. This correlation can be quantified under the assumption of restricted optimality (see Methods). The indicator *q*(*u*, *t*) has a simple interpretation which we will illustrate by focusing on its doubly time-integrated version, $\bar{\bar{q}}={\left\langle \;{\bar{b}}_{i}{\bar{d}}_{i}\;\right\rangle }_{i}$. When we seek to predict a neuron’s choice covariance ${\bar{d}}_{i}$ from its tuning ${\bar{b}}_{i}$, then $\bar{\bar{q}}$ is the best regression coefficient (Fig. 3F), so that

$$\begin{array}{c} {\bar{d}}_{i}=\frac{\bar{\bar{q}}}{{\left\langle {\bar{b}}_{j}^{2}\right\rangle }_{j}^{\;}}\;{\bar{b}}_{i}+\xi_{i}. \end{array}$$

The deviations from this prediction are indicated by *ξ*_*i*_, whose variance in turn is measured by the indicator *V* (Fig. 3F). A similar relation holds for the time-dependent indicator *q*(*u*, *t*).

We now seek readout parameters which provide the best fit to the indicators introduced above. We set a number of potential values for parameters *K*, *w*, *t*_*R*_, *σ*_*d*_, and we explore routinely all their possible combinations. For each tested value of the readout ensemble size, *K*, we repeatedly pick a random neural ensemble 𝓔 of size *K* from the pool of neurons recorded by the experimenter, and propose it as the source of the animal’s percept (Fig. 3C). Then, we compute the average indicators across ensembles of similar size (see Methods), which we will denote by ${\left\langle Z^{2}\right\rangle }_{𝓔}$, ${\left\langle q\right\rangle }_{𝓔}$, and ${\left\langle V\right\rangle }_{𝓔}$. Note that all of these indicators depend on the parameters *w*, *t*_*R*_, *K*, and *σ*_*d*_. Finally, we replace the loss function of Case 1 (eq. 11) by the following “statistical” loss function:

$$\begin{array}{c} L\left(w,t_{R},K,\sigma_{d}\right)={\left(Z^{⋆2}-{\left\langle Z^{2}\right\rangle }_{𝓔}^{\;}\right)}^{2}+\lambda \;\int \int \;\;dt\;du\;{\left(q^{⋆}\left(u,t\right)-{\left\langle q\left(u,t\right)\right\rangle }_{𝓔}\right)}^{2}+\mu {\left(V^{⋆}-{\left\langle V\right\rangle }_{𝓔}^{\;}\right)}^{2}\;. \end{array}$$ (16)

The minimum of the loss function then indicates what values of the readout parameters agree best with the recorded data.

#### Network simulations and retrieval of readout parameters

To validate these claims, we have tested our method on synthetic data—which are the only way to control the true parameters of integration, and thus to test our predictions. We implemented a recurrent neural network with *N* = 5000 integrate-and-fire neurons that encodes some input stimulus *s* in the spiking activity of its neurons, and we built a perceptual readout from that network according to our model, with parameters *K* ^⋆^ = 80 neurons, *w* ^⋆^ = 50 ms, $t_{R}^{⋆}=100$ ms, and $\sigma_{d}^{⋆}=1$ stimulus units (see Methods for a description of the network, and supporting S1 Text).

**Fig 4:**
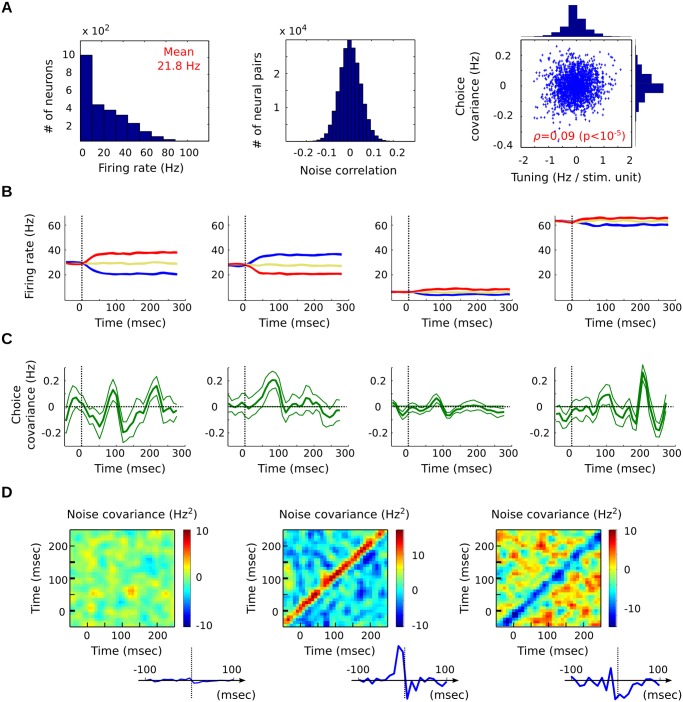
Simulated neural network used for testing the method. (A) Spike count statistics amongst the population of 5000 neurons (spike counts over 400 msec, on 3 × 180 stimulus repetitions). Note the weak, but significant, correlation between tuning (*b*_*i*_) and choice covariance (*d*_*i*_). (B) Sample PSTHs: the model neurons display varied firing rates, and tunings of different polarities. (C) Sample choice covariance curves for the same neurons as panel B (thin lines: bootstrap-based error bars). (D) Sample JPSTHs (noise correlations) for pairs of model neurons. Inset: corresponding cross-correlograms, obtained by projection along the diagonal. For better visibility, the curves in panels B-D were computed from a larger number of trials (3 × 3000) than used for the study itself (3 × 180), and time-averaged with a 10 msec Gaussian kernel.

Then, as experimenters, we observed on every trial the perceptual report *c* ^⋆^ and samples of network activity, from which we computed neural response statistics *b*_*i*_(*t*) and *C*_*ij*_(*t*, *u*), the psychometric curve *ψ* ^⋆^(*s*), and the neuron CC curves $d_{i}^{⋆}(t)$ (Fig. 4). From these (partial) measures, we extracted the three population-wide indicators *Z* ^⋆^, *q* ^⋆^(*u*, *t*) and *V* ^⋆^, and investigated whether the loss function from eq. 16 allows us to recover the system’s true scales of perceptual integration $\left(w^{⋆},t_{R}^{⋆},K^{⋆},\sigma_{d}^{⋆}\right)$.

**Fig 5:**
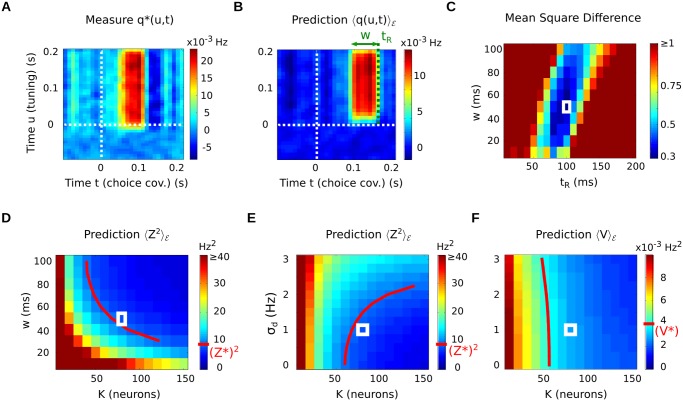
Statistical recovery of readout parameters: structure of the results. (A) Experimental indicator *q* ^⋆^(*u*, *t*). Note the noisiness due to limited amounts of data. (B) Prediction 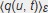 for a given set of readout parameters (*w*, *t*_*R*_, *K*, *σ*_*d*_). The temporal location of the CC signal is mostly governed by parameters *w* and *t*_*R*_. (C) (Normalized) mean square error between measured and predicted *q*(*u*, *t*), as a function of readout parameters (*w*, *t*_*R*_). The true values $\left(w^{⋆},t_{R}^{⋆}\right)$ are indicated by a white square. (D) Predicted JND 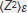 as a function of *K* and *w*. (E) Predicted JND 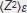 as a function of *K* and *σ*_*d*_. (F) Predicted deviation 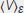 as a function of *K* and *σ*_*d*_. In panels C-F, the white square indicates the true (starred) value for the parameters being represented. The parameters not represented are always fixed at their true (starred) value. In panels D-F, the red curve marks the intersection of the predicted indicator with its measured value. All indicators have units derived from Hz, owing to stimulus *s* being itself a frequency (see Methods).

The results, summarized in Fig. 5, show that the recovery is indeed possible. Each indicator plays a specific role in recovering some of the parameters. First, indicator *q*(*u*, *t*) allows us to recover the temporal parameters of integration (*w*, *t*_*R*_). Indeed, it characterizes the time interval during which the population—as a whole—shows the strongest choice covariance (Fig. 5A), and the bounds of this interval are essentially governed by parameters (*w*, *t*_*R*_) (Fig. 5B). As a result, the match between true measure and prediction—second term in eq. 16—shows a clear optimum near the true values $\left(w^{⋆},t_{R}^{⋆}\right)$ (Fig. 5C). The bi-temporal structure of *q*(*u*, *t*) in eq. 14, with time index *u* corresponding to the neurons’ tuning *b*_*i*_(*u*), stabilizes the results by insuring that *q*(*u*, *t*) is globally positive.

Second, indicator *Z* allows us to target readouts with the correct ‘overall amount’ of integration, resulting in a JND compatible with the data. Fig. 5D depicts the predicted value for *Z* as a function of *w* and *K*. The mark of the ‘*K*-*w* trade-off’ is visible: higher sensitivity to stimulus can be achieved either through longer temporal integration (*w*), or through larger readout ensembles (*K*). Analytically, the JND *Z* depends on *w* because the covariance matrix $\bar{\bar{\text{C}}}$ will generally scale with *w* ^−1^ (under mild assumptions, supporting S1 Text). The red curve marks the pairs (*K*, *w*) for which the prediction matches the measured JND *Z* ^⋆^—thereby minimizing the first loss term in eq. 16. The true parameters (*K* ^⋆^, *w* ^⋆^) lie along that curve (white square in Fig. 5D). Since *w* ^⋆^ is recovered independently thanks to indicator *q*(*u*, *t*), this in turn allows us to recover parameter *K* ^⋆^.

If sensory noise is the main source of error in the animal’s judgments (meaning *σ*_*d*_ ≃ 0 in the model), the two indicators *q*(*u*, *t*) and *Z* suffice to characterize the readout parameters. But in the general case, the observed JND *Z* ^⋆^ can also be influenced by extraneous sources of noise in the animal’s decision, and bias the comparison between *Z* ^⋆^ and its prediction. To account for this potential effect, our model includes the decision-noise term *σ*_*d*_. For a fixed value of *w*, the JND *Z* is influenced both by parameters *K* and *σ*_*d*_ (eq. 13, Fig. 5E). However, both parameters can be disentangled thanks to the third indicator *V*, which depends mostly on *K* (Fig. 5F).

The signification of *V* hinges on the following result, that was first shown in [19]: when the readout is *truly* optimal over the full population (*K* = *N*_tot_), then each neuron’s choice covariance ${\bar{d}}_{i}$ is simply proportional to its tuning ${\bar{b}}_{i}$ (see Methods). Since the indicator *V* quantifies the deviations from perfect proportionality between ${\bar{b}}_{i}$ and ${\bar{d}}_{i}$ (eq. 15, Fig. 3F), it becomes a marker of the readout’s *global* optimality, and decreases to zero as *K* grows to large populations. At the same time, the dependency of *V* on parameter *σ*_*d*_ is minimal, and limited to the influence of the scaling factor *κ*(*Z*) in eq. 9 (see Methods).

When minimizing the loss function in eq. 16, we impose the joint fit of the three indicators *Z*, *q*(*u*, *t*) and *V*. Following the explanations above, this will be obtained for parameters close to the true values $\left(w^{⋆},t_{R}^{⋆},K^{⋆},\sigma_{d}^{⋆}\right)$. In our simulation, the minimum was achieved for the following values: *w* = 50 msec, *t*_*R*_ = 100 msec, *K* = 60 neurons, *σ*_*d*_ = 0.25 stimulus units (with the following levels of discretization: 10 neurons for *K*, 0.25 stimulus units for *σ*_*d*_, 10 msec for *w* and *t*_*R*_).

**Fig 6:**
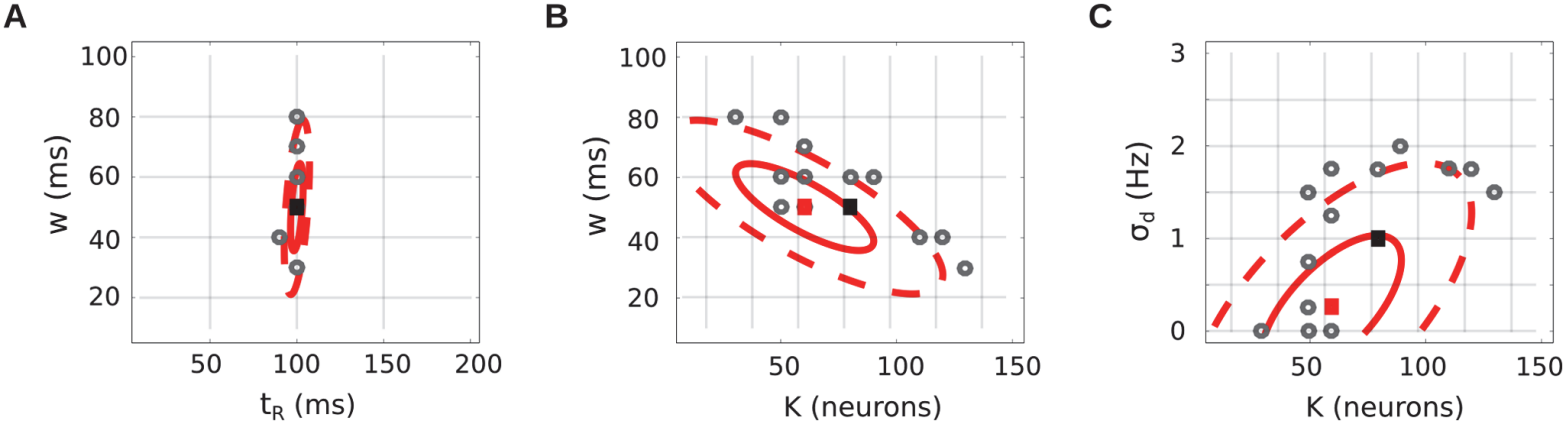
Statistical recovery of readout parameters: best fit parameters. Efficiency of the inference method, applied to our simulated LIF network. The three panels show different 2d projections of the underlying 4d parameter space: (*t*_*R*_, *w*) plane (A), (*K*, *w*) plane (B), (*K*, *σ*_*d*_) plane (C). Black square: true parameters $\left(K^{⋆},w^{⋆},t_{R}^{⋆},\sigma_{d}^{⋆}\right)$ used to produce the data. Red square: best fit parameters (*K*, *w*, *t*_*R*_, *σ*_*d*_), achieving the minimum of the loss function in eq. 16. Gray points: best fit parameters for 14 (bootstrap) resamplings of the original trials (some points are superimposed, due to the finite grid of tested parameters). Red ellipses: corresponding confidence intervals, as the 1- and 2- standard deviation of the bootstrap resamplings.

The best fit parameters are represented in Fig. 6, along with bootstrap confidence intervals derived from 14 resamplings of our original data. The temporal parameters (*w*, *t*_*R*_) are recovered with good precision (panel A). Conversely, parameters *K* and *σ*_*d*_ are somewhat underestimated (panels B and C) compared to their true values (black square). Indeed, the values of *K* and *σ*_*d*_ are disentangled thanks to indicator *V* which, of the three indicators introduced, is the most subject to measurement noise. As a result, the match between *V* ^⋆^ and its prediction *V* is not as precise as the other two: see Fig. 5F. Nevertheless, the true values are rather close to the final estimates, lying within the 1-standard deviation confidence region (Fig. 6C).

Importantly, only a reasonable amount of data is required to produce these estimates. Network activity was monitored on 15 independent runs, each run consisting of 180 repetitions for each of the 3 stimuli. On each run, a different set of *N* = 170 random neurons were simultaneously recorded—out of a total population of *N*_tot_ = 5000. As a result, (i) individual neuron statistics such as *C*_*ij*_(*t*, *u*) or $d_{i}^{⋆}(t)$ display an important amount of measurement noise, (ii) population statistics such as indicator *V* are computed from relatively few neurons *i*. Numerically, this noisiness introduces a number of biases in the above indicators, such as overfitting, which require counteracting with specific corrections (see Methods and supplementary material for details). Naturally, the width of the confidence intervals in Fig. 6 is directly related to the amount of data available.

In conclusion, if the data conform to a number of hypotheses (optimal linear readout from a neural ensemble typical of the full population, and smaller than the recording pool size), then it is possible to estimate the underlying readout’s parameters, from a plausible amount of experimental samples.

### Case 3: less than *K* ^⋆^ cells recorded

By construction, the method presented in Case 2 can only test ensemble sizes *K* smaller than *N*, the number of neurons recorded simultaneously by the experimenter. If *N* is smaller than the true size *K* ^⋆^, the method will provide biased estimates. In current-day experiments, *N* can range from a few tens to a few hundred neurons. While it is not excluded that typical readout sizes *K* ^⋆^ be of that magnitude in real neural populations (as suggested, e.g., by [8]), it is also possible that they are larger. In this case, the only way to estimate the readout parameters is to make specific assumptions about the nature of the full population activity. In turn, the extrapolated results will depend on these assumptions.

#### Singular value analysis of the linear readout

To investigate the underlying issues, and to explain why there is no “natural” extrapolation, we will study how the indicators *Z*, $\bar{\bar{q}}$, and *V* defined above evolve as a function of the number of neurons *K* used for the readout. For simplicity, we assume a fixed choice of (*w*, *t*_*R*_) and focus on the time-integrated neural activities ${\bar{r}}_{i}$ (eq. 1). We also suppose that the decision noise *σ*_*d*_ ≃ 0 is negligible. Finally, we consider alternative definitions for the indicators *Z* and $\bar{\bar{q}}$ that simplify the following analysis. We define

$$\begin{array}{c} Y≔\frac{\sigma_{s}^{2}}{Z^{2}+\sigma_{s}^{2}}, \end{array}$$ (17)

$$\begin{array}{c} Q≔\frac{1}{\kappa (Z)}\;\bar{\bar{q}}+\sigma_{s}^{2}{\left\langle {\bar{b}}_{i}^{2}\right\rangle }_{i}^{}, \end{array}$$ (18)

where $\sigma_{s}^{2}$ is the variance of the tested stimuli, i.e., $\sigma_{s}^{2}≔\text{E}[s^{2}]-\text{E}{\left[s\right]}^{2}$. The *sensitivity* *Y* is simply an inverse reparametrization of the JND, *Z*. More specifically, *Y* is the ratio between the signal-related variance and the total variance (see Methods), which grows from zero (if *Z* = ∞) to one (if *Z* = 0) as the readout’s sensitivity to the stimulus increases. As for *Q*, it is simply a convenient linear rescaling of $\bar{\bar{q}}$.

**Fig 7:**
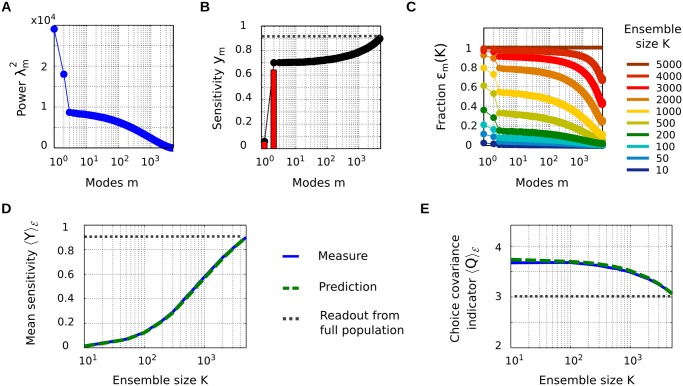
Readout properties as a function of ensemble size *K*. (A) The SVD decomposes population activity into a number of modes *m* with decreasing powers $\lambda_{m}^{2}$. (B) Each mode *m* has a sensitivity to stimulus, *y*_*m*_. Red bars: individual sensitivities, black dots: cumulative distribution. (C) The fractions *ϵ*_*m*_(*K*) describe the “proportion” of each mode which can be observed, on average, in random neural ensembles of size *K*. They are a function of the SVD decomposition, but bear no analytical expression in general. (D) Mean sensitivity from neural ensembles of size *K*, empirically measured (blue) and predicted with eq. 21 (green). The dashed black line indicates the optimal sensitivity, for a readout from the full population. (E) Same for the CC indicator *Q* (eq. 22). All panels computed from the spike counts $\bar{\text{r}}$ of the 5000 simulated neurons, over the first 10 msec after stimulus presentation, on 3 × 3000 recording trials (without correcting for measurement errors owing to the large dimensionality and limited number of trials).

Then, we re-express the population activity through a singular value decomposition (SVD) (see Methods for details). Specifically, we write the time-averaged activity of neuron *i* for the *q*-th presentation of stimulus *s* as

$$\begin{array}{c} {\bar{r}}_{i}^{sq}={\bar{r}}_{i}^{0}+\sum_{m=1}^{M}\lambda_{m}u_{i}^{m}v_{m}^{sq}, \end{array}$$ (19)

where ${\bar{r}}_{i}^{0}$ is the trial-averaged activity of each neuron. This decomposition is best interpreted as a change of variables, which re-expresses the neural activities ${\left\{{\bar{r}}_{i}\right\}}_{i\;=\;1\ldots N_{\text{tot}}}$ in terms of a new set of variables, {*v*_*m*_}_*m* = 1…*M*_, which we will call the activations of the population’s *modes*. These modes can be viewed as the underlying “patterns of activity” that shape the population on each trial. Each mode *m* has a strength *λ*_*m*_ > 0 which describes the mode’s overall impact on population activity. We assume *λ*_1_ ≥ … ≥ *λ*_*M*_, so we progressively include modes with lower strengths (Fig. 7A). The vector **u** ^*m*^ is the “shape” of mode *m* and describes how the mode affects the individual neurons. Finally, $v_{m}^{sq}$ is the mode’s activation variable, which takes a different (random) value on every trial *q* for a given stimulus *s*. The number of modes *M* is the intrinsic dimensionality of the neural population’s activity. In real populations we may expect *M* < *N*_tot_, because neural activities are largely correlated.

Since the singular value decomposition is simply a linear coordinate transform, we can redefine all quantities with respect to the activity modes. Of particular interest is the *sensitivity* of each mode, which is the square of its respective tuning parameter, or (see Methods)

$$\begin{array}{c} y_{m}={\left[\sigma_{s}\sum_{i=1}^{N_{\mathrm{tot}}}\lambda_{m}^{-1}u_{i}^{m}{\bar{b}}_{i}^{}\right]}^{2}. \end{array}$$ (20)

If the readout vector **a** is chosen optimally over the full population, the resulting percept’s sensitivity will be a simple sum over the modes: *Y* ^tot^ = ∑_*m*_ *y*_*m*_.

The mode sensitivities and their cumulative sum for the simulated network above are shown in Fig. 7B. Note the presence of a “dominant” mode for the sensitivity. This seems to be a rather systematic effect, which arises because the definition of total covariance (Methods, eq. 50) favors the appearance of a mode almost collinear with $\bar{b}$. Even so, this dominant mode accounted only for 71% of the population’s total sensitivity, so the residual sensitivity in the other modes is generally not negligible.

#### Sensitivity and choice covariance as a function of the size *K* of the readout ensemble

However, we wish to study the more general case where the readout is built from sub-ensembles of size *K*. In such a case, not all modes are equally observable, and we rather need to introduce a set of fractions, {*ϵ*_*m*_(*K*)}_*m* = 1…*M*_, that express to what extent each mode *m* is “observed”, on average, in sub-ensembles 𝓔 of size *K* (see Methods for a precise definition). Modes with larger power *λ*_*m*_ tend to be observed more, so *ϵ*_*m*_(*K*) globally decreases with *m*. Conversely, *ϵ*_*m*_(*K*) naturally increases with *K*. For the full population, *ϵ*_*m*_(*N*_tot_) = 1 for all modes *m*, meaning that all modes are fully observed (see Fig. 7C; here, the mode observation fractions were empirically computed by averaging over random neural sub-ensembles). Using these fractions, we can analytically approximate the values of *Y* and *Q* which are expected, on average, if the readout is based on ensembles of size *K*:

$$\begin{array}{c} {\left\langle Y\right\rangle }_{𝓔}\left(K\right)≃\sum_{m=1}^{M}\epsilon_{m}\left(K\right)y_{m}\;, \end{array}$$ (21)

$$\begin{array}{c} {\left\langle Q\right\rangle }_{𝓔}\left(K\right)≃\frac{1}{{\left\langle Y\right\rangle }_{𝓔}\left(K\right)}\sum_{m=1}^{M}\epsilon_{m}\left(K\right)y_{m}\lambda_{m}^{2}\;. \end{array}$$ (22)

Thus, the sensitivity ⟨*Y*⟩_𝓔_ grows with *K* as mode sensitivities *y*_*m*_ are progressively revealed by the fractions *ϵ*_*m*_(*K*). The sensitivity reaches its maximum value, *Y*(*N*_tot_) = *Y* ^tot^, when *ϵ*_*m*_(*K*) = 1 for all modes *m* with a nonzero *y*_*m*_ (Fig. 7D). Conversely, ⟨*Q*⟩_𝓔_ decreases with *K*. Indeed, it can be viewed as an average of the squared powers $\left\{\lambda_{m}^{2}\right\}$, each mode *m* contributing with a weight *ϵ*_*m*_(*K*)*y*_*m*_. As *ϵ*_*m*_(*K*) progressively reveals modes with lower power *λ*_*m*_, this average power is expected to decrease with *K*. Again, the minimum value is reached when all nonzero *y*_*m*_ are revealed (Fig. 7E).

The results for the simulated network in Fig. 7D-E illustrate that the approximations leading to eq. 21–22 are well justified in practice. As for the third indicator used in Case 2, *V*, it can also be expressed in the SVD basis (see Methods). However, being a second-order variance term, its approximation based solely on the average fractions {*ϵ*_*m*_(*K*)}, as in eq. 21–22, is generally poor.

#### The extrapolation problem revisited

What do these results imply in terms of extrapolation to larger neural ensembles than those recorded by the experimenter? Arguably, eq. 21–22 constitute an interesting basis for principled extrapolations to larger sizes *K*. These equations show that the evolution of *Y* and *Q* in growing ensembles of size *K* is mostly related to the interplay between the modes’ sensitivity spectrum {*y*_*m*_} and their power spectrum {*λ*_*m*_}. (Empirically, the observation fractions {*ϵ*_*m*_(*K*)} seem primarily governed by the decay rate of {*λ*_*m*_}, although the analytical link between the two remains elusive.) However, note that the spectra {*y*_*m*_}, {*λ*_*m*_} and {*ϵ*_*m*_(*K*)} are generally not accessible to the experimenter—this would precisely require to have recorded at least *N* > *M* neurons, and potentially the whole neural population if *M* = *N*_tot_.

To extrapolate sensitivity ⟨*Y*⟩_𝓔_(*K*) in ensembles of size *K* larger than those monitored, one must (implicitly or explicitly) assume a model for {*λ*_*m*_} and {*y*_*m*_}—which amounts to characterizing the relative embedding of signal and noise in the full population [28]. A number of reasonable heuristics could be used to produce such a model. For example, one may assume a simple distribution for {*λ*_*m*_}, such as a power law, and estimate its parameters from recorded data. Alternatively, it is often assumed that the noise covariance matrix is “smooth” with respect to the signal covariance matrix, so that the former can be predicted on the basis of the latter [19, 29]. Finally, the extrapolation could rely on more specific assumptions about how neural activities evolve, e.g., through linear dynamics with additive noise [30]. In all cases, the additional assumptions impose (implicit) constraints on the structure of the spectra {*λ*_*m*_} and {*y*_*m*_}.

However, most likely, any chosen model will be (1) difficult to fit rigorously on the basis of experimental data, (2) subject to pathological situations when extrapolations fail to produce the correct predictions. For example, one can imagine scenarios in which the most sensitive modes (those with highest *y*_*m*_) correspond to very local circuits of neurons, independent from the rest of the population, and thus invisible to the experimenter (see also [19]). Another pathological situation could be a neural network specifically designed to dispatch information non-redundantly across the full population [31, 32], resulting in a few ‘global’ modes of activity with very large SNR—meaning high *y*_*m*_ and low *λ*_*m*_. As a result, extrapolation to neural populations larger than those recorded is never trivial, and always subject to some *a priori* assumptions. The most judicious assumptions, and the extent to which they are justified, will depend on each specific context.

## Discussion

We have proposed a framework to interpret sensitivity and choice signals in a standard model of perceptual decision-making. Our study describes percept formation within a full sensory population, and proposes novel methods to estimate its characteristic readout scales on the basis of realistic samples of experimental data. Here, we briefly discuss the underlying assumptions and their restrictions, the possibility of further extensions, and the applicability to real data.

### The linear readout assumption

The readout model (eq. 2) used to analyze sensitivity and choice signals is an installment of the “standard”, feed-forward model of percept formation [17, 19]. As such it makes a number of hypotheses which should be understood when applying our methods to real experimental data. First, it assumes that the percept $\hat{s}$ is built linearly from the activities of the neurons—a common assumption which greatly simplifies the overall formalism (but see, e.g., [33] for a recent example of nonlinear decoding). Even if the real percept formation departs from linearity, fitting a linear model will most likely retain meaningful estimates for the coarse information (temporal scales, number of neurons involved) that we seek to estimate in our work.

More precisely, the model in eq. 1–2 assumes that spikes are integrated using a kernel that is separable across neurons and time, that is *A*_*i*_(*t*) = *a*_*i*_ *h*(*t*/*w*)/*w*. Theory does not prevent us from studying a more general integration, where each neuron *i* contributes with a different time course *A*_*i*_(*t*). The readout’s characteristic equations are derived equally well in that case. Rather, assuming a separable form reflects our intuition that the time scale of integration is somewhat uniform across the population. This time scale, *w*, is then the one crucial parameter of the integration kernel. Although the shape *h*(*t*) of the kernel could also be fit from data in theory, it seems more fruitful to assume a simple shape from the start. We assumed a classic square kernel in our applications. Other shapes may be more plausible biologically, such as a decreasing exponential mimicking synaptic integration by downstream neurons. However, given that our goal is to estimate the (coarse) time scale of percept formation, our method will likely be robust to various simple choices for *h*. As a simple example, we tested our method, assuming a square kernel, on data produced by an exponential readout kernel, and still recovered the correct parameters *w*, *t*_*R*_ and *K* (data not shown).

Through the process of integration across time and neurons, each instant in time could be associated to an “ongoing percept”, i.e., the animal’s estimate of stimulus value at current time. In our model, the animal’s estimate at time *t*_*R*_ serves as the basis for its behavioral report (Fig. 2A), and we designate this single number $\hat{s}$ as the “percept”. A second strong assumption of our model is that this perceptual readout occurs at the same time *t*_*R*_ on every stimulus presentation. In reality, there is indirect evidence that *t*_*R*_ could vary from trial to trial, as suggested by the subjects’ varying reaction times (RT) when they are allowed to react freely [34, 35]. In such tasks, we expect the variations in *t*_*R*_ to be moderate—because subjects generally react as fast as they can—and we may even try to correct for fluctuations across trials by measuring RTs. On the other hand, when subjects are forced to wait for a long period of time before responding, there is room for ample variations in *t*_*R*_ from trial to trial, and the model presented above may become insufficient.

**Fig 8:**
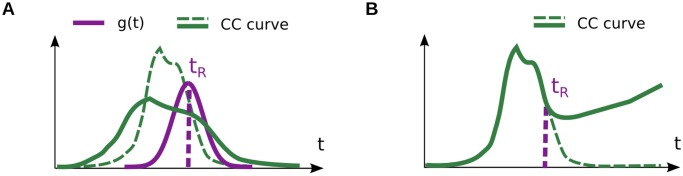
Discussion. (A) If the extraction time *t*_*R*_ varies strongly from trial to trial (with density *g*(*t*)), it leads to a flattening of CC signals (thick green curve) compared to the case with deterministic *t*_*R*_ (dashed green curve). (B) If a choice-related signal feedbacks into sensory areas, it leads to an increase of CC signals (thick green curve) after the extraction time *t*_*R*_, compared to the case without feedback (dashed green curve).

As a first step towards addressing this question, we derived a more general version of the characteristic equations 4–6 assuming that *t*_*R*_ in eq. 1 is itself a random variable, drawn on each trial following some probability distribution *g*(*t*) (supporting S1 Text). The main impact of this modification is on CC curves, which become broader and flatter; essentially, the resulting curve resembles a convolution of the deterministic CC curve by *g*(*t*) (Fig. 8A). This means that if a behavioral task is built such that *t*_*R*_ can display strong variations from trial to trial, the methods introduced above will produce biased estimates. In theory, this issue could be resolved by adding an additional parameter in the analysis, to describe *g*(*t*) (see supporting S1 Text).

### The decision model

The linear readout provides a percept $\hat{s}$ on every trial. In principle, behavioral experiments could be set up such that the subject directly reports this percept, so that $c=\hat{s}$. Such experiments could be treated completely without a decision model. However, almost all experiments that have been studied in the past involve a more indirect report of the animal’s percept. In these cases, some assumptions about how the percept is transformed into the behavioral report *c* need to be made.

In the choice of a decision model, we have followed the logic of the classic random dot motion discrimination task [3], in which a monkey observes a set of randomly moving dots whose overall motion is slightly biased towards the left (*s* < 0 in our notations) or towards the right (*s* > 0). The monkey must then press either of two buttons depending on its judgment of the overall movement direction. The simplest decision model assumes a fixed integration time window, additive noise on the percept, $\hat{s}$, and an optimal binary decision. A slightly more sophisticated model, the “integration-to-bound” model, assumes that the integration time is not fixed, but rather limited by a desired behavioral accuracy. This model requires variable readout windows, rather than the fixed readout window assumed here, and will require further investigation in the future.

In another classic task [2], the monkey must discriminate the frequencies *s*_1_ and *s*_2_ of two successive vibrating stimuli on their fingertip. They must press either of two buttons depending on whether they consider that *s*_1_ > *s*_2_ or not. In this task, the optimal behavioral model would be $c=H({\hat{s}}_{1}-{\hat{s}}_{2})$. In reality, however, the monkey needs to memorize *s*_1_ for a few seconds before *s*_2_ is presented, so potential effects of memory loss may also come into play (see e.g. [36] for a study of these problems).

More generally, behaving animals can display biases, lapses of attention, various exploratory and reward-maximization policies that lead to deviations from the optimal behavioral model. Choosing a relevant behavioral model is a connected problem that cannot be addressed here, and that will vary depending on the task and individual considered. For most tractable behavioral models, the predicted sensitivities and choice signals will ultimately rely on the quantities introduced in this article.

### The feedforward assumption

Finally, the standard model assumes that percept formation is exclusively feed-forward. The activities *r*_*i*_(*t*) of the sensory neurons are integrated to give rise to the percept $\hat{s}$ and the animal’s choice *c*, yet the formation of this decision does not affect sensory neurons in return. Recent evidence suggests that reality is more complex. By looking at the temporal evolution of CP signals in V2 neurons during a depth discrimination task, Nienborg and Cumming (2009) evidenced dynamics which are best explained by a top-down signal, biasing the activity of the neurons on each trial after the choice is formed [20]. In our notations, the population spikes *r*_*i*_(*t*) would thus display a choice-dependent signal which kicks in on every trial after time *t*_*R*_, resulting in CC signals that deviate from their prediction in the absence of feedback (Fig. 8B).

What descriptive power does our model retain, if such top-down effects are strong? The answer depends on the nature of the putative feedback. If the feedback depends linearly on percept $\hat{s}$ (and thus, on the spike trains), its effects are fully encompassed in our model. Indeed, this feedback signal will then be totally captured by the neurons’ linear covariance structure *C*_*ij*_(*t*, *u*), so that our predictions will naturally take it into account. On the other hand, if the feedback depends directly on the choice *c*—which displays a nonlinear, “all-or-none” dependency on $\hat{s}$—then it will not be captured by our model, and lead to possible biases. Even so, our model would still apply if percept and decision were essentially uncoupled before the putative extraction time *t*_*R*_, in which case one could simply compare true and predicted CC signals up to (candidate) time *t*_*R*_ (see Fig. 8B).

### Undersampled neural populations

In most real-life situations, experimenters only have access to samples from a large, unknown population, so they must resort to a statistical description of readout vector **a**. Our solution relies on an assumption of restricted optimality, based on Fisher’s linear discriminant formula (eq. 12). By assuming that readout is made optimally from some unknown neural ensemble 𝓔, we reformulated the problem of characterizing **a** in that of characterizing 𝓔, and could in turn exploit the characteristic equations 4–6 statistically.

In real experiments, the true readout profile **a** may not match this description: most vectors **a** do not implement optimal readout from a sub-ensemble. This potential discrepancy from the true readout is inescapable, once we start representing **a** through a statistical model. However, note that our model uses *two* distinct sources of non-optimality: (1) the size *K* of the readout ensemble, which can be much smaller than the full population, and (2) the decision noise *σ*_*d*_, which adds a ‘global’ non-optimality to the readout. Arguably, by combining both factors, our chosen model for **a** will be flexible enough to provide meaningful estimates when fit to real data.

At present, the main limitation is likely to be the size of ensembles of neurons that have been recorded simultaneously. Past work has often shown that small ensembles of neurons are completely sufficient to account for an animal’s behavior [3, 37]. However, there is an inherent trade-off between the number of neurons and the time scale of integration. One simple explanation for the small sizes of previous readout ensembles is that the true readout time scales used by subjects are much shorter. Unfortunately, as detailed above (Case 3), extrapolations from a finite-size recording onto the whole population always come at the price of strong additional assumptions.

However, as experimental techniques advance, and as the number of simultaneously recorded neurons reaches the number of neurons implied in the readout, we will eventually be able to directly infer the readout parameters from the data. In this case, our method can readily be tested on real data, and hopefully provide new insights into the nature of percept formation from populations of sensory neurons.

## Methods

The methods are organized as follows. First, we set our basic notations and definitions. Second, we derive the characteristic equations of the model, both for the linear part and decision part. Third, we detail the predictions in case of an optimal readout from some neural sub-ensemble 𝓔. Fourth, we re-express these predictions in the basis of the population’s SVD modes. Finally, we detail our methodology to empirically estimate the quantities used in this article, from limited amounts of experimental data. Tables 1–3 summarize the main variables and notations used in the article.

### Statistical notation

In the following, we generally deal with variables *x* that assume different values on different trials. An example is the spike count of a single neuron. Trials in turn can be grouped by stimulus *s* or choice *c*. We can make this explicit by writing *x* ^*scq*^ to denote the *q*-th trial in which the stimulus was *s* and the subject’s choice was *c*. Given such a variable, we will write E[*x*] for its expectation value, i.e., for the hypothetical value this quantity would take if it could be averaged over infinitely many trials. We will write E[*x*∣*s*] for the expectation value conditioned on stimulus *s*, i.e., for the expectation value computed over all trials in which the stimulus was *s*. A similar notation holds when conditioning on choices *c*. We note that for quantities that are already conditional expectations, for instance, *y*(*s*) = E[*x*∣*s*], their expectation value E[*y*(*s*)] will average out the stimuli according to their relative probabilities, i.e., E[*y*(*s*)] = ∑_*s*_ *p*(*s*)*y*(*s*). Thereby, each stimulus *s* contributes to the expectation in proportion to the number of trials associated to it. Then the notations are coherent, since we have E[E[x∣s]]=E[x]. Covariances are generically defined as Cov[*x*, *y*] = E[*xy*] − E[*x*]E[*y*], and variances as Var[*x*] = Cov[*x*, *x*]. For vectorial quantities, we assume Cov[**x**, **y**] = E[**x** **y** ^⊤^] − E[**x**]E[**y** ^⊤^], and introduce the shorthand Cov[**x**] ≔ Cov[**x**, **x**].

### Experimental statistics of neural activity and choice

Classic measures in decision-making experiments can be interpreted as estimates of the first- and second-order statistics of choice *c* and recorded spike trains *r*_*i*_(*t*), across all trials with a fixed stimulus value *s*:

$$\begin{array}{c} \psi (s)≔E[c|s], \end{array}$$ (23)

$$\begin{array}{c} m_{i}\left(t\;;s\right)≔E[r_{i}\left(t\right)|s], \end{array}$$ (24)

$$\begin{array}{c} C_{ij}\left(t,u\;;s\right)≔\mathrm{Cov}[r_{i}\left(t\right),r_{j}\left(u\right)|s], \end{array}$$ (25)

$$\begin{array}{c} d_{i}\left(t\;;s\right)≔\mathrm{Cov}[r_{i}\left(t\right),c|s]. \end{array}$$ (26)

Here, *ψ*(*s*) is the psychometric curve, *m*_*i*_(*t*; *s*) is known as the PSTH, and *C*_*ij*_(*t*, *u*; *s*) as the JPSTH. The choice covariance (CC) curve *d*_*i*_(*t*; *s*) is our proposal for measuring each neuron’s “choice signal”. Theoretically, the temporal signals in eq. 24–26 are well-defined quantities in the framework of continuous-time point processes [38]. In practice, they are estimated by binning spike trains *r*_*i*_(*t*) with a finite temporal precision, depending on the amount of data available.

From the psychometric curve, we also derive two simpler quantities: the animal’s *just-noticeable difference* (JND), *Z*, and *decision bias* *μ*_*d*_. We obtain them as the best (MSE) fit to the following formula:

$$\begin{array}{c} \psi (s)=\text{Φ}\left(\frac{s+\mu_{d}-s_{0}}{Z}\right), \end{array}$$ (27)

where Φ is the standard cumulative normal distribution. *Z* measures the inverse slope of the psychometric curve (up to a scaling factor $\sqrt{2\pi }$). The decision bias *μ*_*d*_, when non-zero, represents a bias towards one button when *s* = *s*_0_. This formula for the psychometric curve arises naturally when we model the decision task (see below).

### Choice covariance and choice probability

Throughout the article, we consider the special case of a binary choice *c* = {0, 1}. In this case, the variance of the choice conditioned on *s* is given by

$$\begin{array}{c} \sigma_{c}^{2}\left(s\right)≔\mathrm{Var}\left[c|s\right]=\psi \left(s\right)\left(1-\psi \left(s\right)\right), \end{array}$$ (28)

and a straightforward computation shows that

$$\begin{array}{c} d_{i}\left(t\;;s\right)=\sigma_{c}^{2}\left(s\right)\;\left(E\left[r_{i}\left(t\right)|s,c=1\right]-E\left[r_{i}\left(t\right)|s,c=0\right]\right). \end{array}$$ (29)

(These formulas, and all those below, assume that the choice takes values 0 and 1. Any other binary parametrization should first be reparametrized to {0, 1}.)

The term in brackets is the difference between the two conditional PSTHs, computed only from trials where the animal took one decision vs. the other (stimulus *s* keeping a fixed value). This measure is sometimes used as a simpler alternative to choice probabilities [3]. In fact, CC curves and CP curves can be analytically related if one assumes Gaussian statistics: see [19] or supporting S1 Text.

### Simplified dependencies on the stimulus

The neural statistics in eq. 24–26 are defined conditionally for each stimulus *s* used in the task. To ease the subsequent analysis, we assume that the activity of each neuron is well approximated by a time-varying, linear dependency on the stimulus *s*, and that *C*_*ij*_(*t*, *u*; *s*) is independent of *s*. Consequently,

$$\begin{array}{cc} m_{i}\left(t\;;s\right) & =m_{i}^{0}\left(t\right)+b_{i}\left(t\right)s,\\ C_{ij}\left(t,u\;;s\right) & =C_{ij}\left(t,u\right). \end{array}$$

Since we are modeling a discrimination task, in which stimuli *s* display only small variations around the central value *s*_0_, the linearity assumption seems reasonable. In turn, we can write

$$\begin{array}{c} b_{i}\left(t\right)≔\partial_{s}E\left[r_{i}\left(t\right)|s\right]. \end{array}$$ (30)

We will refer to *b*_*i*_(*t*) as the neural tuning. More precisely, it is the slope of the neuron’s tuning curve at each time point.

Naturally, actual data (even from a synthetic simulation) always somewhat deviate from this idealized situation. In practice, we obtain the best fits for *b*_*i*_(*t*) and *C*_*ij*_(*t*, *u*) using linear regression, so that

$$\begin{array}{c} b_{i}\left(t\right)=\frac{E\left[sm_{i}\left(t;s\right)\right]-E\left[s\right]E\left[m_{i}\left(t;s\right)\right]}{E\left[s^{2}\right]-E{\left[s\right]}^{2}}, \end{array}$$ (31)

$$\begin{array}{c} C_{ij}\left(t,u\right)=E[\;C_{ij}\left(t,u\;;s\right)]. \end{array}$$ (32)

Similarly, it is convenient to integrate the various CC curves *d*_*i*_(*t*; *s*) (eq. 26) into a single CC curve for each neuron, say *d*_*i*_(*t*). There is no obvious choice for this simplification, because *d*_*i*_(*t*; *s*) has to change with *s*. For example, the CC signal is non-zero only if stimulus *s* and threshold *s*_0_ are close enough for the animal to make occasional mistakes (this is reflected in eq. 29, since $\sigma_{c}^{2}(s)$ tends to zero when the animal makes no mistakes). In the experimental literature, a common choice is to focus only on the CC curve at threshold, that is *d*_*i*_(*t*) = *d*_*i*_(*t*; *s*_0_). In experiments with a limited number of trials, this has the inconvenience of losing the statistical power from nearby stimulus values *s* that were also tested. We thus propose an alternative definition:

$$\begin{array}{c} d_{i}\left(t\right)≔E[d_{i}\left(t\;;s\right)], \end{array}$$ (33)

which exploits each stimulus *s* in proportion to the number of associated trials. In our model, this averaging also limits the influence of the JND *Z* on the magnitude of CC signals: see eq. 45–46.

### Derivation of the linear characteristic equations

The readout defined in eq. 1–2 is linear with respect to the underlying spike trains {*r*_*i*_(*t*)}. To clarify the equations, let us introduce the temporal averaging kernel

$$\begin{array}{c} k\left(t\;|w,t_{R}\right)≔\frac{1}{w}h\left(\frac{t_{R}-t}{w}\right), \end{array}$$ (34)

where parameters *w* and *t*_*R*_ are generally implicit. Then, the integrated spike counts from eq. 1 are simply ${\bar{r}}_{i}=\int_{t}r_{i}(t)k(t)dt$.

Using this notation, eq. 2 becomes $\hat{s}=a_{0}+\sum_{i}\int_{t}a_{i}k(t)r_{i}(t)dt$. Thanks to the linear structure, the two first moments of $\hat{s}$ can easily be developed:

$$\begin{array}{c} E[\hat{s}|s]=a_{0}+\sum_{i}a_{i}\int_{t}E\left[r_{i}\left(t\right)|s\right]k\left(t\right)dt, \end{array}$$

$$\begin{array}{c} \mathrm{Var}[\hat{s}|s]=\sum_{ij}a_{i}a_{j}\int_{u}\int_{t}\;\mathrm{Cov}\left[r_{i}\left(t\right),r_{j}\left(u\right)|s\right]k\left(t\right)k\left(u\right)dt\;du, \end{array}$$

$$\begin{array}{c} \mathrm{Cov}[r_{i}\left(t\right),\hat{s}|s]=\sum_{j}a_{j}\int_{u}\mathrm{Cov}\left[r_{i}\left(t\right),r_{j}\left(u\right)|s\right]k\left(u\right)du. \end{array}$$

Given our various definitions (eq. 24–25), and after differentiating the first line with respect to *s*, see eq. 30, we obtain:

$$\begin{array}{ccc} \partial_{s}E\left[\hat{s}|s\right]=\sum_{i}a_{i}\int_{t}b_{i}\left(t\right)k\left(t\right)dt & & =a^{⊤}\bar{b}, \end{array}$$ (35)

$$\begin{array}{ccc} \mathrm{Var}[\hat{s}|s]=\sum_{ij}a_{i}a_{j}\int_{u}\int_{t}C_{ij}\left(t,u\right)k\left(t\right)k\left(u\right)dt\;du & & =a^{⊤}\bar{\bar{C}}a, \end{array}$$ (36)

$$\begin{array}{ccc} \mathrm{Cov}[r_{i}\left(t\right),\hat{s}|s]=\sum_{j}a_{j}\int_{u}C_{ij}\left(t,u\right)k\left(u\right)du & & ={\left[\bar{C}\left(t\right)a\right]}_{i}. \end{array}$$ (37)

These are exactly the characteristic equations 4–6 from the main text, after introducing the following vectors and matrices:

$$\begin{array}{c} {\bar{b}}_{i}≔\int_{t}b_{i}\left(t\right)k\left(t\right)dt, \end{array}$$ (38)

$$\begin{array}{c} {\bar{C}}_{ij}\left(t\right)≔\int_{u}C_{ij}\left(t,u\right)k\left(u\right)du, \end{array}$$ (39)

$$\begin{array}{c} {\bar{\bar{C}}}_{ij}≔\int_{t}{\bar{C}}_{ij}\left(t\right)k\left(t\right)\;dt, \end{array}$$ (40)

$$\begin{array}{c} {\bar{d}}_{i}≔\int_{t}d_{i}\left(t\right)k\left(t\right)dt, \end{array}$$ (41)

which simply correspond to the statistics of activity for the integrated spike counts ${\bar{r}}_{i}$ (eq. 1). Indeed, ${\bar{b}}_{i}=\partial_{s}\text{E}[{\bar{r}}_{i}|s]$ (tuning vector), ${\bar{\bar{C}}}_{ij}=\text{Cov}[{\bar{r}}_{i},{\bar{r}}_{j}|s]$ (noise covariance matrix), and ${\bar{d}}_{i}=\text{E}[\text{Cov}[{\bar{r}}_{i},c|s]]$ (choice covariance vector). Given our assumptions above, the resulting quantities are all independent of the stimulus *s*. Note though, that all quantities depend on the readout parameters *w* and *t*_*R*_. Importantly, one can show that the noise covariance matrix $\bar{\bar{\text{C}}}$ scales as *w* ^−1^, under mild assumptions (supporting S1 Text, section 2).

### The decision model of a fine-discrimination task

To produce a binary choice, the (continuous) percept $\hat{s}$ is fed into the decision model $c=H(\hat{s}-s_{0}+\xi_{d})$, where *H* is the Heaviside function, *s*_0_ is the (task-imposed) decision threshold, and *ξ*_*d*_ ∼ 𝒩(*μ*_*d*_, *σ*_*d*_) is a Gaussian variable representing additional noise and biases. The mean *μ*_*d*_ implements a possible bias towards one button when *s* = *s*_0_. The standard deviation *σ*_*d*_ implements additional sources of noise in the animal’s decision process.

Using this decision model, and mild additional assumptions, we can relate the left-hand sides of eq. 35–37 to experimental data. First, we assume that $\text{E}[\hat{s}|s]=s$, meaning that $\hat{s}$ follows *s* on average. (In statistical terminology, $\hat{s}$ is an *unbiased estimator* of *s*.) Then, the left-hand side of eq. 35 is simply equal to

$$\begin{array}{c} \partial_{s}E\left[\hat{s}|s\right]=1. \end{array}$$ (42)

Second, we assume that the distribution of **r**(*t*) (given *s*) is Gaussian. (In theory, this assumption is violated at small time scales due to the binary nature of *r*_*i*_(*t*). But in practice this is not an issue, as the spike trains always undergo some form of temporal integration afterwards.) Then, $\hat{s}$ (given *s*) is normally distributed, and eq. 36 ensures that its variance $\text{Var}[\hat{s}|s]$ is independent of *s* (see Fig. 2B). In these conditions, the predicted formula for the psychometric curve is exactly that of eq. 27, namely,

$$\begin{array}{c} \psi \left(s\right)=\text{Φ}\left(\frac{s+\mu_{d}-s_{0}}{Z}\right), \end{array}$$

and the JND, *Z*, is given by the following expression:

$$\begin{array}{c} Z^{2}=\mathrm{Var}\left[\hat{s}|s\right]+\sigma_{d}^{2}. \end{array}$$ (43)

Furthermore, under the same assumptions, we can predict the CC curve for each neuron. We use the following general result: for any bivariate normal variables (*X*, *Y*) and threshold *t*, Cov[*X*, *H*(*Y* − *t*)] = Cov[*X*, *Y*]𝒢(*t*; *μ*_*Y*_, *σ*_*Y*_), where 𝒢(⋅; *μ*, *σ*) is the normal density function. Here, we take *X* = *r*_*i*_(*t*), $Y=\hat{s}+\xi_{d}$ and *t* = *s*_0_, to obtain:

$$\begin{array}{c} d_{i}\left(t\;;s\right)=𝓖\left(s\;;\;s_{0}-\mu_{d},Z\right)\;\mathrm{Cov}\left[r_{i}\left(t\right),\hat{s}|s\right]. \end{array}$$ (44)

With *d*_*i*_(*t*) defined as an average CC curve over tested stimuli (eq. 33), we finally obtain

$$\begin{array}{c} d_{i}\left(t\right)=\kappa \left(Z\right)\;\mathrm{Cov}[r_{i}\left(t\right),\hat{s}|s], \end{array}$$ (45)

$$\begin{array}{c} \mathrm{with}\;\kappa (Z)=E\left[𝓖(s\;;\;s_{0}-\mu_{d},\;Z)\right]. \end{array}$$ (46)

The final equation for CC signals (eq. 9) is obtained by combining eq. 37 and 45.

In many experimental setups, the averaging over stimuli *s* will ensure that *κ*(*Z*) has only a mild dependency on its argument *Z*. Indeed, note the rough approximation *κ*(*Z*) ∝ ∫_*s*_d*s*𝒢(*s*; *s*_0_−*μ*_*d*_, *Z*) = 1, valid whenever the tested stimuli *s* are uniformly distributed over a range of values comparable to *Z*. This is another practical argument for considering the stimulus-averaged CC signal *d*_*i*_(*t*), from eq. 33.

### Signal, noise, and sensitivity

The just-noticeable difference (JND) and the sensitivity can be related to the variances of signal and noise in the population. Here, we briefly review these relations. The variance of any scalar variable *x* that changes from trial to trial can be decomposed in a signal term $\sigma_{x}^{2}≔\text{Var}[\text{E}[x|s]]$ and a noise term $Z_{x}^{2}≔\text{E}[\text{Var}[x|s]]$. Then, note that $\text{Var}[x]=\sigma_{x}^{2}+Z_{x}^{2}$.

The noise term *Z*_*x*_ defines the minimal level past which fluctuations in *x* can be attributed to *s* rather than intrinsic noise—hence the term JND. When a decision is taken on the basis of variable *x*, the JND governs the inverse slope of the corresponding psychometric curve (see eq. 27). We also define the *sensitivity* of variable *x* as

$$\begin{array}{c} Y_{x}≔\frac{\sigma_{x}^{2}}{\sigma_{x}^{2}+Z_{x}^{2}}, \end{array}$$ (47)

which is simply the ratio of the signal to the total variance. The sensitivity *Y*_*x*_ takes values between 0 and 1. It thus avoids singularities which may occur when *Z*_*x*_ tends to 0 or +∞.

We can also distinguish between signal-related and noise-related variance for the (time-averaged) neural activities $\bar{\text{r}}$. The signal covariance matrix, **Σ**, noise covariance matrix, $\bar{\bar{\text{C}}}$, and total covariance matrix, **A**, are given by the following relations:

$$\begin{array}{c} \text{Σ}≔\mathrm{Cov}\left[E[\bar{r}|s]\right]=\mathrm{Cov}\left[\bar{m}\left(s\right)\right]=\sigma_{s}^{2}\;\bar{b}{\bar{b}}^{⊤} \end{array}$$ (48)

$$\begin{array}{c} \bar{\bar{C}}≔E\left[\mathrm{Cov}[\bar{r}|s]\right] \end{array}$$ (49)

$$\begin{array}{c} A≔\mathrm{Cov}\left[\bar{r}\right]=\bar{\bar{C}}+\sigma_{s}^{2}\;\bar{b}{\bar{b}}^{⊤}. \end{array}$$ (50)

The last equality is the classic decomposition of total covariance into noise and signal terms. Note that **Σ** is a rank-1 matrix, owing to the system’s assumed linearity wrt. stimulus *s*.

In turn, these matrices allow to compute the signal- and noise- variances for any weighted sum of the neural activities. For our linear readout (with added decision noise *ξ*_*d*_), we have $x={\text{a}}^{⊤}\bar{\text{r}}+\xi_{d}$, and thus:

$$\begin{array}{c} \sigma_{x}^{2}=a^{⊤}\text{Σ}a=\sigma_{s}^{2}{\left(a^{⊤}\bar{b}\right)}^{2}, \end{array}$$ (51)

$$\begin{array}{c} Z_{x}^{2}=a^{⊤}\bar{\bar{C}}a+\sigma_{d}^{2}, \end{array}$$ (52)

$$\begin{array}{c} \sigma_{x}^{2}+Z_{x}^{2}=a^{⊤}Aa+\sigma_{d}^{2}. \end{array}$$ (53)

### Optimal readout from a neural ensemble 𝓔

We now assume that the readout vector **a** has support only on some neural ensemble 𝓔. Formally, we introduce the *K* × *N*_tot_ projection matrix **H**(𝓔), such that for *i* ∈ 𝓔 and every neuron *j*, *H*_*ij*_(𝓔) = *δ*_*ij*_. Then, the restrictions of vectors and matrices in neuron space, such as $\bar{\text{b}}$ and $\bar{\bar{\text{C}}}$, to ensemble 𝓔 will be denoted by a subscript *r* (for restriction), so that

$$\begin{array}{c} {\bar{b}}_{r}≔H\bar{b}, \end{array}$$ (54)

$$\begin{array}{c} {\bar{\bar{C}}}_{r}≔H\bar{\bar{C}}H^{⊤}. \end{array}$$ (55)

Our principle of (restricted) optimality selects the readout vector **a** which maximizes the signal-to-noise ratio of the resulting percept $\hat{s}$. Since ${\text{a}}^{⊤}\bar{\text{b}}=1$ (unbiased percept, eq. 35 and 42), the signal variance is imposed to be $\sigma_{x}^{2}=\sigma_{s}^{2}$ (eq. 51). Under this constraint, optimality is achieved by minimizing the noise variance ${\text{a}}^{⊤}\bar{\bar{\text{C}}}\text{a}$ (eq. 52)—or equivalently, the total variance **a** ^⊤^ **A** **a** (eq. 53). The solution, known as Fisher’s Linear Discriminant, is easily found with Lagrange multipliers (either based on $\bar{\bar{\text{C}}}$ or **A**):

$$\begin{array}{c} a_{r}=\frac{\;{\left({\bar{\bar{C}}}_{r}\right)}^{-1}{\bar{b}}_{r}^{\;}}{{\bar{b}}_{r}^{⊤}{\left({\bar{\bar{C}}}_{r}\right)}^{-1}{\bar{b}}_{r}^{\;}}=\frac{\;{\left(A_{r}\right)}^{-1}{\bar{b}}_{r}^{\;}}{{\bar{b}}_{r}^{⊤}{\left(A_{r}\right)}^{-1}{\bar{b}}_{r}^{\;}}. \end{array}$$ (56)

The second formulation of **a**_*r*_, based on the total covariance matrix **A**_*r*_, will prove more useful when we turn to the SVD analysis. It also has the advantage of avoiding the singularity which may occur when vector ${\bar{\text{b}}}_{r}$ lies outside the span of matrix ${\bar{\bar{\text{C}}}}_{r}$. In that case one simply replaces (**A**_*r*_)^−1^ by the (Moore-Penrose) pseudoinverse (**A**_*r*_)^+^.

When combining the optimal readout in eq. 56 with the equation for the JND (eq. 52), we obtain the JND predicted by the model:

$$\begin{array}{c} Z^{2}={\left({\bar{b}}_{r}^{⊤}{\left({\bar{\bar{C}}}_{r}\right)}^{-1}{\bar{b}}_{r}^{\;}\right)}^{-1}+\sigma_{d}^{2}. \end{array}$$ (57)

Equivalently, using the formulations based on total variance (eq. 47, 53, 56) we obtain the model’s prediction for sensitivity:

$$\begin{array}{c} Y=\frac{\sigma_{s}^{2}}{{\left({\bar{b}}_{r}^{⊤}{\left(A_{r}\right)}^{-1}{\bar{b}}_{r}\right)}^{-1}+\sigma_{d}^{2}}. \end{array}$$ (58)

### CC signals for the optimal readout

When combining the optimal readout in eq. 56 with the characteristic equation for the CC curves (eq. 9), we obtain the CC curves predicted by the model,

$$\begin{array}{c} d_{i}\left(t\right)=\kappa \left(Z\right)\left(Z^{2}-\sigma_{d}^{2}\right)\;{\bar{C}}_{ir}\left(t\right){\left({\bar{\bar{C}}}_{r}\right)}^{-1}{\bar{b}}_{r}^{\;}. \end{array}$$ (59)

Here, *d*_*i*_(*t*) is the resulting, predicted CC curve for every neuron *i* in the population (not only in ensemble 𝓔). Note that ${\bar{\text{C}}}_{ir}(t)$ is the restriction of vector ${\bar{\text{C}}}_{i}(t)$ (eq. 39) to neurons *j* ∈ 𝓔, but that *i* = 1…*N*_tot_ still runs over all neurons. Equation 59 can also be expressed in its temporally-integrated form, using the definition $\int_{t}\bar{\text{C}}(t)k(t)dt=\bar{\bar{\text{C}}}$:

$$\begin{array}{c} {\bar{d}}_{i}=\kappa \left(Z\right)\left(Z^{2}-\sigma_{d}^{2}\right)\;{\bar{\bar{C}}}_{ir}{\left({\bar{\bar{C}}}_{r}\right)}^{-1}{\bar{b}}_{r}^{\;}. \end{array}$$ (60)

If neuron *i* belongs to the readout ensemble 𝓔, matrix ${\bar{\bar{\text{C}}}}_{r}$ simplifies away from eq. 60, yielding:

$$\begin{array}{c} {\bar{d}}_{i}^{\left(𝓔\right)}=\kappa \left(Z\right)\left(Z^{2}-\sigma_{d}^{2}\right)\;{\bar{b}}_{i}^{\left(𝓔\right)}. \end{array}$$ (61)

This equation, first shown in [19], means that choice signals within the readout ensemble are simply proportional to tuning. This is not true, however, for neurons outside the readout ensemble.

This has two important implications. First, it proves that choice signals are markedly different for neurons inside or outside the readout ensemble (an observation made empirically by [12]). Second, as we consider readout ensembles 𝓔 larger and larger, eq. 61 will become true for more and more neurons. As a result the statistical indicator *V* (eq. 15), which measures the population-wide deviation from linearity between ${\bar{d}}_{i}$ and ${\bar{b}}_{i}$, is expected to decrease with the readout ensemble’s size *K*.

Finally, under the assumption of (restricted) optimality, the time-averaged statistical indicator $\bar{\bar{q}}$ is always positive. Indeed, averaging over all neurons *i* in the population is akin to a scalar product: $\bar{\bar{q}}={\left\langle \;{\bar{b}}_{i}{\bar{d}}_{i}\right\rangle }_{i}=N_{\text{tot}}^{-1}\;{\bar{\text{b}}}^{⊤}\bar{\text{d}}$. Using this relation and eq. 60, we get

$$\begin{array}{c} \bar{\bar{q}}=N_{\mathrm{tot}}^{-1}\;\kappa \left(Z\right)\left(Z^{2}-\sigma_{d}^{2}\right)\;{\bar{b}}^{⊤}\left(\bar{\bar{C}}H^{⊤}{\left({\bar{\bar{C}}}_{r}\right)}^{-1}H\right)\bar{b}\;, \end{array}$$ (62)

which is always positive because both matrices $\bar{\bar{\text{C}}}$ and ${\text{H}}^{⊤}{\left({\bar{\bar{\text{C}}}}_{r}\right)}^{-1}\text{H}$ are symmetric semi-definite positive.

### Singular value decomposition

We denote the time-averaged activities of neuron *i* in the *q*-th presentation of stimulus *s* as ${\bar{r}}_{i}^{sq}$. We interpret these activities as a very large *N*_tot_ × Ω matrix, where *N*_tot_ refers to the number of neurons and Ω to an idealized, and essentially infinitely large number of trials.

Next, we consider the singular value decomposition (SVD) of the neural activities. The (compact) SVD is a standard decomposition which can be applied to any rectangular matrix **R**. It is given by **R** = **U** **Λ** **V** ^⊤^, where **Λ** is an *M* × *M* diagonal matrix with strictly positive entries *λ*_*m*_ (the singular values), **U** is an *N*_tot_ × *M* matrix of orthogonal columns (meaning **U** ^⊤^ **U** = **Id**_*M*_), and **V** is an Ω × *M* matrix of orthogonal columns (meaning **V** ^⊤^ **V** = **Id**_*M*_).

Using the indices defined above, the SVD decomposition for the neural activities becomes

$$\begin{array}{c} {\bar{r}}_{i}^{sq}={\bar{r}}_{i}^{0}+\sum_{m=1}^{M}\lambda_{m}u_{i}^{m}v_{m}^{sq}, \end{array}$$ (63)

where ${\bar{r}}_{i}^{0}$ is the average activity of each cell over all trials and stimuli. The orthogonality of **U** implies that for all indices *m* and *n*, we have $\sum_{i}u_{i}^{m}u_{i}^{n}=\delta^{mn}$, while the orthogonality of **V** similarly implies $\sum_{sq}(v_{m}^{sq}v_{n}^{sq})=\delta_{mn}$.

### Statistics of activity, in the space of modes

The SVD decomposition (eq. 63) is best interpreted as a change of variables re-expressing neural activities ${\left\{{\bar{r}}_{i}^{sq}\right\}}_{i=1\ldots N_{\text{tot}}}$ in terms of mode appearance variables ${\left\{v_{m}^{sq}\right\}}_{m=1\ldots M}$. As a result, we can define the respective equivalents of all statistical quantities in the space of activity modes. Specifically, we can reinterpret sums over trials in the SVD as expectations, thus emphasizing the statistical interpretation of the SVD. First we note that ${\bar{r}}_{i}^{0}=\text{E}[{\bar{r}}_{i}^{sq}]$ for all neurons *i*, so that the data for the actual SVD has been “centered”. This centering implies for all modes *m* that

$$\begin{array}{c} E[v_{m}^{sq}]=0, \end{array}$$ (64)

$$\begin{array}{c} E[v_{m}^{sq}|s]=\eta_{m}\left(s-E[s]\right), \end{array}$$ (65)

where *η*_*m*_ is the tuning parameter of the *m*-th mode, just as ${\bar{b}}_{i}$ was the tuning parameter for the *i*-th neuron. Grouping all mode appearance variables in a vector **v**, we obtain the signal covariance and total covariance matrices in mode space as

$$\begin{array}{c} {\text{Σ}}_{v}≔\mathrm{Cov}\left[E[v|s]\right]=\mathrm{Cov}\left[\eta s\right]=\sigma_{s}^{2}\eta \eta^{⊤}, \end{array}$$ (66)

$$\begin{array}{c} A_{v}:=\mathrm{Cov}\left[v\right]=E\left[vv^{⊤}\right]={\mathrm{Id}}_{M}. \end{array}$$ (67)

where the last relation follows from the orthogonality of **V** explained in the previous section. The singular values *λ*_*m*_ and distribution vectors **u** ^*m*^ then allow us to relate the statistics at the levels of neurons and modes. Using the SVD formula (eq. 63) yields (in matrix form):

$$\begin{array}{c} \bar{b}=U\Lambda \eta , \end{array}$$ (68)

$$\begin{array}{c} A=U\Lambda^{2}U^{⊤}. \end{array}$$ (69)

### Sensitivity of sub-ensembles, in the space of modes

We now wish to understand which factors govern the sensitivity embedded in a neural sub-ensemble 𝓔 of cardinality *K*. For simplicity, we will consider the case for which the decision noise is negligible, i.e., *σ*_*d*_ → 0. Then, from eq. 58, we have

$$\begin{array}{c} Y=\sigma_{s}^{2}\;{\bar{b}}_{r}^{⊤}{\left(A_{r}\right)}^{+}{\bar{b}}_{r}^{\;}. \end{array}$$ (70)

Here we use explicitly the most general formula, based on the pseudo-inverse of matrix **A**_*r*_. To re-express this sensitivity of finite sub-ensembles 𝓔 into mode space, we need to find the equivalent, restricted expressions of eq. 68–69. For that purpose, we introduce the *design matrix* associated to ensemble 𝓔 in mode space:

$$\begin{array}{c} X≔\text{Λ}U^{⊤}H^{⊤}, \end{array}$$ (71)

where **H** is the restriction operator from eq. 54. **X** is an *M* × *K* matrix with elements $x_{i}^{m}≔\lambda_{m}u_{i}^{m}$. Using this matrix, we obtain from eq. 68–69 that ${\bar{\text{b}}}_{r}={\text{X}}^{⊤}\text{Ζ}$ and **A**_*r*_ = **X** ^⊤^ **X**, so that eq. 70 becomes

$$\begin{array}{cc} Y & =\sigma_{s}^{2}\;\eta^{⊤}X{\left(X^{⊤}X\right)}^{+}X^{⊤}\eta \\ & =\sigma_{s}^{2}\;\eta^{⊤}P\eta , \end{array}$$ (72)

where we have defined the *M* × *M* matrix

$$\begin{array}{c} P≔X{\left(X^{⊤}X\right)}^{+}X^{⊤}. \end{array}$$ (73)

Note that **P** is simply the orthogonal projector on Im(X), since **P** = **P** ^2^ = **P** ^⊤^, and Im(**P**) = Im(**X**).

The projector **P** = **P**(𝓔) spans more and more space as the size *K* of ensemble 𝓔 increases. In the limiting case, when *K* is larger than the number of modes *M*, then necessarily **P** = **Id**_*M*_, and we obtain

$$\begin{array}{c} Y_{\mathrm{tot}}=\sigma_{s}^{2}\eta^{⊤}\eta =\sum_{m=1}^{M}\sigma_{s}^{2}\eta_{m}^{2}. \end{array}$$ (74)

In other words, all modes are available experimentally, and sensitivity estimates saturate to their maximum value, independently of ensemble 𝓔. We can explicitly denote the sensitivity of each mode’s activation variable *v*_*m*_ by defining

$$\begin{array}{c} y_{m}≔\sigma_{s}^{2}\eta_{m}^{2}. \end{array}$$ (75)

By solving eq. 68 for ***η***, we obtain $\eta_{m}=\sum_{i}\lambda_{m}^{-1}u_{i}^{m}{\bar{b}}_{i}$, which in turn yields eq. 20 from the main text.

### CC signals, in the space of modes

Similarly, we can express CC signals in mode space. First, we re-express the CC equation (eq. 10) as a function of the total covariance **A** (eq. 50) to obtain

$$\begin{array}{cc} \bar{d} & =\kappa \left(Z\right)\;\bar{\bar{C}}a\\ & =\kappa \left(Z\right)\;\left(A-\sigma_{s}^{2}\bar{b}{\bar{b}}^{⊤}\right)a. \end{array}$$

We further recall that ${\text{a}}^{⊤}\bar{\text{b}}=1$ (unbiased percept, see eq. 35 and 42). Hence, up to a scaling and shift, the CC vector $\bar{\text{d}}$ can be replaced by the *total percept covariance* vector

$$\begin{array}{c} e≔Aa=\kappa {\left(Z\right)}^{-1}\;\bar{d}+\sigma_{s}^{2}\;\bar{b}. \end{array}$$ (76)

In the case of an optimal readout, vector **a** is given by eq. 56, so that we obtain

$$\begin{array}{c} e=\frac{AH^{⊤}A_{r}^{+}{\bar{b}}_{r}}{{\bar{b}}_{r}^{⊤}{\left(A_{r}\right)}^{+}{\bar{b}}_{r}}. \end{array}$$ (77)

Second, using the corresponding sensitivity *Y* (eq. 70), and the SVD expressions for **A** and $\bar{\text{b}}$ (eq. 68–69), and for **A**_*r*_ and ${\bar{\text{b}}}_{r}$ as a function of matrix **X** (eq. 71), we write:

$$\begin{array}{cc} e & =\sigma_{s}^{2}\;Y^{-1}AH^{⊤}A_{r}^{+}{\bar{b}}_{r}\\ & =\sigma_{s}^{2}\;Y^{-1}\;U\Lambda X{\left(X^{⊤}X\right)}^{+}X^{⊤}\eta \\ & =\sigma_{s}^{2}\;Y^{-1}\;U\Lambda P\eta . \end{array}$$ (78)

Here also, the final result can be expressed as a function of **P**, the projection matrix associated to ensemble 𝓔 in the space of modes (eq. 73). Note again that **e** provides the CC signal for every neuron *i* in the population (not only in ensemble 𝓔). As 𝓔 tends to the full population, **P** = **P**(𝓔) tends to **Id**_*M*_ and we recover $\text{e}(\infty )=\sigma_{s}^{2}Y_{\text{tot}}^{-1}\;\bar{\text{b}}$, the prediction for choice signals in the case of a (globally) optimal readout [19].

Using eq. 78, we can finally compute the analytical predictions for the two CC statistical indicators, $\bar{\bar{q}}$ and *V*. Precisely, we compute the following population-wide regression coefficient between **e** and $\bar{\text{b}}$:

$$\begin{array}{cc} Q≔ & {\left\langle e_{i}{\bar{b}}_{i}\right\rangle }_{i}\\ = & N_{\mathrm{tot}}^{-1}\;{\bar{b}}^{⊤}e\\ = & \sigma_{s}^{2}\;N_{\mathrm{tot}}^{-1}Y^{-1}\;\eta^{⊤}\Lambda U^{⊤}U\Lambda P\eta \\ = & \sigma_{s}^{2}\;N_{\mathrm{tot}}^{-1}Y^{-1}\;\eta^{⊤}\Lambda^{2}P\eta . \end{array}$$ (79)

Again, we made use of the SVD expressions for $\bar{\text{b}}$ (eq. 68) and **e** (eq. 78). Note that, since **e** is a linear rescaling of $\bar{\text{d}}$, *Q* is a similar rescaling of indicator $\bar{\bar{q}}$, as pointed in the main text (eq. 18). Finally, a very similar computation leads to the expression of indicator *V* (eq. 15) in the space of modes:

$$\begin{array}{c} V=\kappa {\left(Z\right)}^{2}\;N_{\mathrm{tot}}^{-2}\;\sigma_{s}^{4}\;Y^{-2}\;\eta^{⊤}\Lambda^{2}\left(\eta \eta^{⊤}P-P\eta \eta^{⊤}\right)\Lambda^{2}P\eta . \end{array}$$ (80)

### Sensitivity and CC signals as a function of *K*

We are now better armed to understand how sensitivity and CC indicators vary as a function of the readout ensemble 𝓔. We are mostly interested in averages of these quantities over very large numbers of randomly chosen ensembles 𝓔 of size *K*; we thus use the generic notation E[*x*∣*K*]≔E[*x*(𝓔)∣Card(𝓔) = *K*] to denote the expected value of a variable *x* when averaging over ensembles of size *K*. Note that this notation is equivalent to the more explicit notation used in the main text, so that E[*x*∣*K*] = ⟨*x*⟩_𝓔_(*K*). From eq. 72 we find: $\text{E}[Y|K]=\sigma_{s}^{2}\;\eta^{⊤}\;\text{E}[\text{P}|K]\;\eta$.

To understand the properties of the (*M* × *M*) matrix E[**P**∣*K*], we view the (*M* × *K*) design matrix **X**(𝓔) (eq. 71) as a collection of *K* random vectors **x**_*i*_ in mode space, viewing neuron identities *i* as the random variable. Thus, **P**(𝓔) is the orthogonal projector on the linear span of the *K* sample vectors {**x**_*i*_}_*i* ∈ 𝓔_. As a projector, its trace is equal to its rank, so we have Tr(E[P∣K])=K. Furthermore, since *K*+1 samples span on average more space than *K* samples, we are ensured that E[**P**∣*K*+1] ≽ E[**P**∣*K*], in the sense of positive semidefinite matrices.

Finally, intuition and numerical simulations suggest that E[**P**∣*K*] is almost diagonal. Indeed, as the various modes are linearly independent, there is no linear interplay between the different dimensions of **x**_*i*_ across samples *i*. More precisely, the expectation value over neurons is ${\left\langle x_{i}^{m}x_{i}^{n}\right\rangle }_{i}^{\;}=N_{\text{tot}}^{-1}\lambda_{m}^{2}\delta^{mn}$. This leads to the matrix expression:

$$\begin{array}{c} E\left[XX^{⊤}|K\right]=KN_{\mathrm{tot}}^{-1}\Lambda^{2}. \end{array}$$

Let us consider the (compact) SVD decomposition **X** **X** ^⊤^≔ **W** **D** **W** ^⊤^, with **W** ^⊤^ **W** = **Id**, and **D** an invertible diagonal matrix. Then, the projection matrix **P** is simply equal to **W** **W** ^⊤^. As for the previous equation, it rewrites

$$\begin{array}{c} E\left[WDW^{⊤}|K\right]=KN_{\mathrm{tot}}^{-1}\Lambda^{2}. \end{array}$$

Here, both matrices **D** and **Λ** are diagonal. So, if we assume a form of independence between **W** and **D**, it is reasonable to suppose that E[**W** **W** ^⊤^∣*K*] = E[**P**∣*K*] is close to diagonal as well. (Actually, we postulate that E[**P**∣*K*] is exactly diagonal when the random vectors **x**_*i*_ follow a normal distribution. In the general case, small or moderate deviations from diagonality can be observed.) We denote these diagonal terms as

$$\begin{array}{c} \epsilon (K)≔\text{diag}(E[P|K]). \end{array}$$ (81)

The properties of E[**P**∣*K*] stated above imply that ∑_*m*_ *ϵ*_*m*_(*K*) = *K* (trace property), and *ϵ*_*m*_(*K*+1) ≥ *ϵ*_*m*_(*K*) (growth property). Finally, we can consider the resulting approximations of sensitivity (eq. 72) and CC indicator (eq. 79):

$$\begin{array}{c} E[Y|K]≃\sigma_{s}^{2}\sum_{m=1}^{M}\epsilon_{m}\left(K\right)\eta_{m}^{2}, \end{array}$$ (82)

$$\begin{array}{c} E[YQ|K]≃N_{\mathrm{tot}}^{-1}\sigma_{s}^{2}\;\sum_{m=1}^{M}\epsilon_{m}\left(K\right)\lambda_{m}^{2}\eta_{m}^{2}. \end{array}$$ (83)

In this expression, we recognize the individual mode sensitivities $y_{m}=\sigma_{s}^{2}\eta_{m}^{2}$. For CC signals, we also make the approximation E[*YQ*∣*K*] ≃ E[*Y*∣*K*]E[*Q*∣*K*], and recover eq. 21–22 from the main text. Unfortunately, there is no such simple approximation for indicator *V*, that would lead from eq. 80 to E[*V*∣*K*].

### Validation on a simulated neural network

In this final part of the Methods, we provide additional information for applying our inference method (Case 2) to experimental data. The neural network used to test our methods is described in detail in supporting S1 Text (section 3). Briefly, on each trial, 2000 input Poisson neurons fire with rate *s*, taking one of three possible values 25, 30 and 35 Hz (so in our simulation, stimulus units are Hz). The encoding population *per se* consists of 5000 leaky integrate-and-fire (LIF) neurons. 1000 of these neurons receive sparse excitatory projections from the input Poisson neurons, which naturally endows them with a positive tuning to stimulus *s*. Another 1000 neurons receive sparse inhibitory projections from the Poisson neurons, which naturally endows them with negative tuning. The remaining 3000 neurons receive no direct projections from the input. Instead, all neurons in the encoding population are coupled through a sparse connectivity with random delays up to 5 msec. Synaptic weights are random and balanced, leading to a mean firing rate of 21.8 Hz in the population. We implemented and simulated the network using Brian, a spiking neural network simulator in Python [39].

The “true” perceptual readout from this network was built from a fixed random set of *K* ^⋆^ = 80 neurons, with temporal parameters *w* ^⋆^ = 50 msec and $t_{R}^{⋆}=100$ msec, and decision noise $\sigma_{d}^{⋆}=1$ stimulus units (Hz). The readout vector **a** ^⋆^ was built optimally given these constraints (eq. 12). The trials used to learn **a** ^⋆^ were not used in the subsequent analysis. The resulting JND for the “animal” was *Z* ^⋆^ ≈ 3 stimulus units (Hz).

Then, “experimentally”, neural activity was observed through 15 pools of 170 simultaneously recorded neurons, each pool being recorded on 3 × 180 trials. For the statistical inference method, we assumed a square integration kernel *h*. We tested all combinations of the following readout parameters (in matrix notation): *K* = 10:10:150 neurons, *w* = 10:10:100 msec, *t*_*R*_ = 10:10:200 msec, *σ*_*d*_ = 0:0.25:3 stimulus units (Hz). For each tested size *K*, we picked 2000 random candidate ensembles 𝓔 (always within one of the 15 simultaneous pools) to build the predictions. For each ensemble 𝓔, another ensemble ℐ of 20 neurons, segregated from 𝓔, were used to predict CC signals outside the readout ensemble (this was always possible since recording pools had size 170, and *K* ≤ 150). The details of these predictions are explained in the following paragraph. Finally, the three terms in the “statistical” loss function (eq. 16) were weighted according to the power of the respective, true measures. That is:

$$\begin{array}{c} \lambda =\frac{{\left(Z^{⋆}\right)}^{4}}{\;\int \int \;\;dt\;du\;{\left(q^{⋆}\left(u,t\right)\right)}^{2}}\;\mathrm{and}\;\mu =\frac{{\left(Z^{⋆}\right)}^{4}}{{\left(V^{⋆}\right)}^{2}}. \end{array}$$

### Experimental predictions for CC indicators

Here, we detail how to compute the CC indicators *q*(*u*, *t*) and *V* (eq. 14–15) from actual data. For the *measured* versions *q* ^⋆^(*u*, *t*) and *V* ^⋆^(*w*, *t*_*R*_), this is straightforward. One considers the true, *measured* CC signals $d_{i}^{⋆}(t)$, and computes the population averages in eq. 14–15 over as many neurons *i* as were recorded. Note however that the final indicators can be corrupted by noise, whenever each measure $d_{i}^{⋆}(t)$ comes from too few recording trials (this problem is addressed in the next section). Also note that, since the definition of *V* requires a temporal integration, we actually have to produce a different “true” *V* ^⋆^ for each tested set of temporal parameters *w* and *t*_*R*_.

Conversely, special care must be taken when it comes to *predicted* CC indicators. Whenever a candidate ensemble 𝓔 is proposed as the source of the readout, eq. 59 predicts the resulting CC signal *d*_*i*_(*t*∣𝓔) for every neuron *i* in the population. However, in practice, the noise covariance term ${\bar{\text{C}}}_{ir}(t)$ is required in the computation, so neuron *i* and ensemble 𝓔 must have been recorded simultaneously during the same run. This limits the number of neurons *i* which can participate in the population averages.

Furthermore, choice covariances will generally differ between neurons that are part of the readout ensemble and neurons that are not (see eq. 61 and the associated discussion). As a result, the two following averages must be predicted separately:

$$\begin{array}{c} q_{𝓔}^{}\left(u,t\;|𝓔\right)≔{\left\langle b_{i}\left(u\right)d_{i}\left(t|𝓔\right)\right\rangle }_{i\in 𝓔}, \end{array}$$ (84)

$$\begin{array}{c} q_{\mathrm{out}}\left(u,t\;|𝓔\right)≔{\left\langle b_{i}\left(u\right)d_{i}\left(t|𝓔\right)\right\rangle }_{i\notin 𝓔}, \end{array}$$ (85)

before one can recombine them in the correct proportions:

$$\begin{array}{c} p(𝓔)≔\frac{K}{N_{\mathrm{tot}}}, \end{array}$$ (86)

$$\begin{array}{c} q(u,t\;|𝓔)=p\left(𝓔\right)q_{𝓔}^{}\left(u,t\;|𝓔\right)+\left(1-p(𝓔)\right)q_{\mathrm{out}}\left(u,t\;|𝓔\right), \end{array}$$ (87)

and similarly for *V*(𝓔). To compute $q_{\text{out}}^{}$ experimentally, each tested candidate ensemble 𝓔 (of size *K*) is associated to a complimentary set of neurons ℐ (of size *I*), which we use to approximate the average in eq. 85:

$$\begin{array}{c} q_{𝓘}^{}\left(u,t\;|𝓔\right)≔{\left\langle b_{i}\left(u\right)d_{i}\left(t|𝓔\right)\right\rangle }_{i\in 𝓘}. \end{array}$$ (88)

All neurons in ensembles 𝓔 and ℐ must have been recorded during the same run, which imposes that *I*+*K* ≤ *N*. Hence in our simulations, we chose a size *I* = 170−150 = 20 neurons.

Clearly, 20 neurons is not sufficient for *q*_ℐ_ to be a reliable population average. So in practice, we cannot estimate reliably each prediction *q*(*u*, *t* ∣𝓔) from eq. 87. Luckily, we are not interested in their value for each individual readout ensemble 𝓔. We simply need to estimate their means across all tested ensembles 𝓔 of similar size:

$$\begin{array}{c} {\left\langle q\left(u,t\right)\right\rangle }_{𝓔}^{\;}≔{\left\langle q(u,t|𝓔)\right\rangle }_{𝓔\;\mathrm{with}\;\mathrm{Card}\;(𝓔)=K} \end{array}$$ (89)

$$\begin{array}{c} {\left\langle V\right\rangle }_{𝓔}^{\;}≔{\left\langle V(𝓔)\right\rangle }_{𝓔\;\mathrm{with}\;\mathrm{Card}\;(𝓔)=K} \end{array}$$ (90)

which will be reliable as soon as we test a sufficient amount of candidate ensembles 𝓔.

Note that in the final inference (eq. 16), a match is sought between the true indicators *q* ^⋆^ and *V* ^⋆^—which arise from a single readout ensemble 𝓔^⋆^, and the predictions ${\left\langle q\right\rangle }_{𝓔}^{\;}$ and ${\left\langle V\right\rangle }_{𝓔}^{\;}$—which are average values across all readout ensembles 𝓔 of size *K*. Thus, a prediction error can occur whenever the true readout ensemble 𝓔^⋆^ is not a “typical” representative of its size *K* ^⋆^. To quantify these potential errors, one should also estimate the indicators’ *variance* across ensembles 𝓔 of same size.

### Correcting for the finite amounts of data

The computations of *Z*, *q* and *V*, as described above, can produce imprecise results when the data are overly limited. Generically, for any quantity *X* estimated from the data, we can write

$$\begin{array}{c} X_{\mathrm{noisy}}=X_{\mathrm{ideal}}+\xi , \end{array}$$

where *ξ* represents the measurement error on *X* due to the finite amounts of data. If we could recompute *X* from a different set of neurons and/or a different set of trials, variable *ξ* would take a different value—meaning that Var(*ξ*) > 0. This is an inescapable phenomenon for experimental measures.

More problematically, variable *ξ* can display a systematic *bias*, meaning that E(*ξ*) ≠ 0. Since the bias is generally different for the ‘true’ and ‘predicted’ versions, the comparison between the two (eq. 16) will be systematically flawed. To counteract this effect, we applied a number of correction procedures when computing indicators *Z*, *q* and *V*, to ensure that they are globally unbiased. We only provide an overview here, and refer to supporting S1 Text for a detailed description.

First, when the optimal vector **a** is computed with Fisher’s linear discriminant, it systematically underestimates the JND *Z* (overestimates the sensitivity *Y*). Essentially, vector **a**_*r*_ computed through eq. 12 finds artificial “holes” in matrix ${\bar{\bar{\text{C}}}}_{r}$ which are only due to its imprecise measurement—a phenomenon known as statistical *overfitting*. The less recording trials, the more overfitting there will be [40, 41]. We addressed this problem with a regularization technique, inspired by Bayesian linear regression [42]. We replaced eq. 12 by the following:

$$\begin{array}{c} a_{r}=\frac{\;{\left({\bar{\bar{C}}}_{r}+\lambda \mathrm{Id}\right)}^{-1}{\bar{b}}_{r}^{\;}}{{\bar{b}}_{r}^{⊤}{\left({\bar{\bar{C}}}_{r}+\lambda \mathrm{Id}\right)}^{-1}{\bar{b}}_{r}^{\;}}, \end{array}$$

where the strength of parameter *λ* imposes the degree of regularization. We chose *λ* according to an ‘empirical Bayes’ principle, to maximize the likelihood of the data under a given statistical model (supporting S1 Text, section 4). It largely mitigated the effects of overfitting, without totally suppressing them—as can be seen in Fig. 5D-E.

Second, indicator *V* (eq. 15) can also display substantial biases (E(*ξ*) ≠ 0 in the above discussion). Indeed, its computation relies on squared quantities—such as ${\bar{d}}_{i}^{2}$ or ${\bar{\bar{q}}}^{2}$—that systematically transform measurement errors into positive biases. The required corrections are very similar to the classic “*N*/(*N*−1)” correction for the naive variance estimator, with the additional difficulty that *V* is affected by *two* sources of noise: the finite number of recording trials, and the finite number of recorded neurons. The exact corrections to ensure an unbiased estimation of *V* are detailed in supporting S1 Text, section 5.

Third, indicator *q*(*u*, *t*) displays little or no measurement bias—because its computation is essentially linear. Yet, it can display an important level of measurement noise (Var(*ξ*) ≫ 0 in the above discussion) that may deteriorate the subsequent inference procedure. We mitigated this measurement noise by applying a bi-temporal Gaussian smoothing to *q* ^⋆^(*u*, *t*) and predictions *q*(*u*, *t*), with time constant 10 msec.

To estimate the measurement errors due to the finite number of trials, we produced 14 sets of surrogate data by sampling our original trials with replacement (bootstrap procedure). These resamplings were used to derive some of the correction terms for *V*, and *also* to derive confidence intervals on our final estimators, as shown in Fig. 6. This departure from the statistical canon was imposed by the length of the whole inference procedure (see supporting S1 Text, section 5, for details).

### Reproduction of our results and implementation

In the Supporting Information, we provide a generic implementation of the inference method (“Case 2” above) in MATLAB, which can be applied to any data from a 2AFC discrimination task. We also provide the Python code for the network simulation, and MATLAB scripts for the reproduction of the experimental Figures in this article (Fig. 4–7).

## Supporting Information

S1 Text Supporting text. Contains additional information about Choice Probabilities (section **1**), the influence of parameter *w* on stimulus sensitivity (section **2**), the encoding neural network used for testing the method (section **3**), the Bayesian regularization procedure on Fisher’s linear discriminant (section **4**), unbiased computation of CC indicators in the presence of measurement noise (section **5**), and an extended readout model with variable extraction time *t*_*R*_ (section **6**).
(PDF)

S1 Compressed file archive Supporting code for the article. (GZ)
