# Supplementary material for: On the Number of Neurons and Time Scale of Integration Underlying the Formation of Percepts in the Brain
Source: PLoS Comput Biol. 2015 Mar 20;11(3):e1004082. doi: 10.1371/journal.pcbi.1004082 (PMC4368836; doi:10.1371/journal.pcbi.1004082)
Supplement: S1 Text — Contains additional information about Choice Probabilities (section 1), the influence of parameter w on stimulus sensitivity (section 2), the encoding neural network used for testing the method (section 3), the Bayesian regularization procedure on Fisher’s linear discriminant (section 4), unbiased computation of CC indicators in the presence of measurement noise (section 5), and an extended readout model with variable extraction time t R (section 6). (PDF) [file pcbi.1004082.s001.pdf]

Supplementary information for the article "*On the number of neurons and time scale of integration underlying the formation of percepts in the brain*".

A. Wohrer, C. K. Machens

Contents

1 Predictions for choice probabilities **1**

2 Sensitivity as a function of w **2**

3 Encoding neural network **3**

4 Bayesian regularization of the optimal readout **6**

5 Unbiased estimation of CC indicators q and V **9**

6 Extended model with non-deterministic extraction time t_R **15**

1 Predictions for choice probabilities

Most often, choice signals are computed in the form of choice probabilities. This first requires to compute some temporal average $\bar{r}_i$ of the underlying spike trains. Then, CP for every neuron i measures the area under the ROC curve between the two distributions of $\bar{r}_i$, respectively conditioned on $c = 0$ and $c = 1$ [5]. With the same assumptions as in our main text (optimal behavioral model, Gaussian statistics), Haefner et al. [6] have shown that the CP signal for each neuron i is approximately proportional to the (Pearson) correlation between $\bar{r}_i$ and $\widehat{s}$, that is:

$$\text{CP}_i - \frac{1}{2} \simeq \frac{\sqrt{2}}{\pi} \frac{\text{Cov}[\bar{r}_i, \widehat{s}]}{\sigma(\bar{r}_i)\sigma(\widehat{s})} = \frac{\sqrt{2}}{\pi} \frac{\kappa(Z)^{-1}\bar{d}_i}{Z\,\sigma(\bar{r}_i)}. \tag{1}$$

The second equality makes the link with the notations of our article. For example, let us consider a typical neuron from our simulations, and its spike count $\bar{r}_i$ over $w = 400$ msec of stimulation (as in main text, Figure 4a). We assume a CC value $\bar{d}_i = 0.1$ Hz, and a Poisson-like firing rate at 20 Hz leading to $\sigma(\bar{r}_i) = \sqrt{20/0.4} \simeq 7$ Hz. The JND for our "animal" is $Z^\star \simeq 3$ Hz, and with our chosen stimuli we have $\kappa(Z^\star) \simeq 0.067$ (eq. 46 from the main text). In these conditions, we recover a typical value $\text{CP}_i \simeq 0.53$.

In our notations, CP_i is predicted to be proportional to the choice covariance $\bar{d}_i$, computed with the same temporal averaging as $\bar{r}_i$. However, CP_i is also inversely proportional to the standard deviation of the spike counts $\sigma(\bar{r}_i)$, which depends on the time window w used to compute them. For example, if we used instead an integration of $w = 40$ msec, we would find $\sigma(\bar{r}_i) = \sqrt{20/0.04} \simeq 22.3$ Hz, and consequently $\text{CP}_i \simeq 0.51$. As a result there is no single "reference" experimental value for CPs. Conversely, choice covariance signals $d_i(t)$ used in our article do not suffer from that complication.

2 Sensitivity as a function of w

In the forms of the main text, it is not explicit how the value of w influences the variance of $\widehat{s}$, and thus the readout's sensitivity. Here we show that under mild assumptions, the doubly-integrated covariance matrix $\bar{\bar{\mathbf{C}}}$ (eq. 40 from the main text) scales as w^{-1}.

To allow a direct comparison between different possible choices for the shape function $h(x)$, we impose that it should be positive ($h(x) \geq 0$), normalized ($\int_x h(x)dx = 1$), and have a unit time constant ($\int_x h(x)^2 dx = 1$). Common choices could include a square kernel, a decreasing exponential, a Gaussian kernel, etc.[1] For concision, we note the integration kernel at scale w: $h_w(u) := w^{-1}h(u/w)$. Then, let us note $G_w(u,v) := h_w(t_R - u)h_w(t_R - v)$. It verifies the following property: $\int_t G_w(t, t+\tau)dt = (h_w \star h_w)(\tau)$, the autocorrelation of kernel h_w. As a result, we can rewrite the variance equation (Methods, eq. 36) in the form:

$$\begin{aligned}
\mathrm{Var}[\widehat{s}|s] &= \sum_{ij} a_i a_j \int_{t,\tau} G_w(t,t+\tau)C_{ij}(t,t+\tau)dt\,d\tau \\
&= \sum_{ij} a_i a_j \int_\tau (h_w \star h_w)(\tau)\Big(\int_t \frac{G_w(t,t+\tau)}{h_w \star h_w(\tau)} C_{ij}(t,t+\tau)dt\Big)d\tau \\
&= w^{-1} \sum_{ij} a_i a_j \int_\tau (h \star h)(\tau/w)\widetilde{C_{ij}}(\tau)d\tau. \qquad (2)
\end{aligned}$$

In the second line, the function of t defined by the fraction is positive and has an integral of 1, so it operates as a temporal averaging on $C_{ij}(t, t+\tau)$. The resulting average over t, noted $\widetilde{C_{ij}}(\tau)$ in the third line, is thus a form of *cross-correlogram* between neurons i and j, measuring the average covariance between the spikes from i and j separated by a time lag τ.

Since the shape h has a unit time constant, its autocorrelation function typically has support on $[-1, 1]$, and verifies $(h \star h)(0) = 1$. On the other hand, $\widetilde{C_{ij}}(\tau)$ typically has support on some interval $[-\tau_C, \tau_C]$, where τ_C is the typical time scale of noise correlations in the population. As a result, as soon as w gets bigger than τ_C, the integral in eq. 2 approaches a constant value, and the variance of $\widehat{s}$ scales as w^{-1}. The variance of $\widehat{s}$ also scales as w^{-1} when $w \to 0$, because all auto-correlograms $\widetilde{C_{ii}}(\tau)$ display a Dirac peak in $\tau = 0$, due to the spiking nature of the neurons [3].

This whole analysis holds similarly in the more general case of a non-deterministic t_R, only with a slightly different definition of kernel $G_w(u, v)$ (supplementary section 6).

[1] Square : $h(x) = \mathbf{1}_{[0,1]}(x)$. Exponential : $h(x) = \mathbf{1}_{\mathbb{R}^+}(x)2\mathrm{e}^{-2x}$. Gaussian : $h(x) = \sqrt{2}\mathrm{e}^{-2\pi x^2}$.

3 Encoding neural network

We detail here the architecture of the artificial encoding network used to test our method. This ad-hoc network was designed to display some classic features of sensory cortical neurons involved in perceptual decision-making tasks (e.g, V2, MT, S1, S2, etc.). To reproduce the diversity of response naturally observed at the population level [7], neurons in our network have broadly distributed firing rates and pairwise noise correlations (main text, Figure 4a). We also wished to reproduce the continuum of stimulus tuning observed in real populations, where some neurons have positive tuning (rate increase when s increases), and other neurons have negative tuning.

The network consists of two distinct layers of spiking neurons, of which only the second layer (encoding layer) is "visible" to the experimenter (Figure 1). The first layer (L1) consists of $2000 = 2 \times 1000$ independent Poisson neurons, whose firing intensity s constitutes the stimulus encoded by the second layer. On each trial, s takes one of three possible values 25, 30 and 35 Hz. All neurons in L1 are equivalent, but segregated in two distinct populations according to their projections on the second layer. The Poisson firing constitutes the only source of randomness in the network from trial to trial.

The second layer (L2) consists of 5000 leaky integrate-and-fire (LIF) neurons, some of which receive input from L1, and who are all coupled through a sparse, balanced connectivity. The neurons are modeled according to the following differential equations for their voltages,

$$\tau \frac{dV_i^{(y)}}{dt} = -V_i^{(y)}(t) + V_{\text{rest}} + I^{(y)} + \sum_{j \in \text{L1}} W_{ji}^{(1,y)} \delta(t - t_j) + \sum_{k \in \text{L2}} W_{ki}^{(2)} \delta(t - t_k - \Delta_{ki}).$$

The neuron emits a spike at each time t_i when $V_i^{(y)}$ reaches threshold V_{thr}, after what the neuron's potential is reinitialized at resting value V_{rest}, and held there during a refractory period of 3 msec. All neurons share the same membrane time constant $\tau = 20$ msec, threshold $V_{\text{thr}} = -50$ mV, and resting potential $V_{\text{rest}} = -60$ mV. Upper index y denotes one of three possible subtypes of neurons in L2: Positively-biased neurons ($y = p$, 1000 neurons), negatively-biased neurons ($y = n$, 1000 neurons) and unbiased neurons ($y = u$, 3000 neurons).

Positively-biased neurons receive sparse excitatory connections from 1000 neurons in L1 ($W_{ji}^{(1,p)} \geq 0$), whereas negatively-biased neurons receive sparse inhibitory connections from the 1000 other neurons in L1 ($W_{ji}^{(1,n)} \leq 0$). Unbiased neurons receive no direct input from L1 ($W_{ji}^{(1,u)} = 0$). The connection matrices $\mathbf{W}^{(1,y)}$ are sparse with (Erdös-Renyi) connection probability $p = 0.01$. As these asymmetries create biases in the total synaptic inputs to each type of cell, the intrinsic currents $I^{(p)}$, $I^{(n)}$ and $I^{(u)}$ also vary depending on neuron subtype, to ensure homogeneous firing properties inside the three populations (see Table 1).

Then, all L2 neurons are connected through a single matrix $\mathbf{W}^{(2)}$ of recurrent connections with (Erdös-Renyi) connection probability $p = 0.03$—independently of their subtype. Non-zero connection strengths are picked uniformly in an interval $[w_{\min}, w_{\max}]$: see Table 1. Note that L2 recurrent connections can be both excitatory and inhibitory, a departure from biology allowing for an easier implementation. Finally, the recurrent connections in L2 are associated to synaptic delays: for each pair (i, k) of connected L2 neurons, the random delay Δ_{ki} is drawn uniformly between 0 and 5 msec. This substantially increases the diversity of neural responses in the population, particularly at the level of JPSTHs (Figure 4d from the main text). This is interesting because our method is specifically designed to analyze generic, heterogeneous population activities.

Subtype	$I^{(y)}$	$w^{(1,y)}_{\min}$	$w^{(1,y)}_{\max}$	$w^{(2)}_{\min}$	$w^{(2)}_{\max}$
Pos. biased (p)	0	0	2	-2	2
Neg. biased (n)	14	-4	0	-2	2
Unbiased (u)	5	0	0	-2	2

Table 1: Connectivity parameters in the three subtypes of L2 neurons. All values are expressed in millivolts.

We implemented and simulated the network using Brian, a spiking neural network simulator in Python [4]. Our simulation consisted of many successive epochs of 300 msec with all possible successions of the three stimulus values s (as in Figure 1a from the main text). Since the input Poisson neurons were always firing close to 30 Hz, there was no strong transient at stimulus onset as is often observed in real sensory neurons. In our case, the change of activity between two successive stimuli was always only differential, and rather weak (see Figure 4b from the main text).

Figure 2 displays additional information (in complement to main text, Figure 4) regarding noise correlations in the network. Panel (a) displays the joint distribution of signal and noise correlations in the network. Here, signal correlation is defined over only three numbers per neuron, namely, the neuron's mean firing rate for each of the three stimuli. As a result of this simplistic definition, most neural pairs have correlations close to 1 (same tuning polarity) or -1 (opposite tuning polarities). The red curve is the average value of the noise correlation, conditioned on the signal correlation. It stays at zero, indicating that noise correlations in the population are balanced, and essentially decoupled from signal correlations.

Panel (b) summarizes the temporal structure of noise correlations in the network. It is constructed as in [1] (their Figure 5D) : for each temporal offset τ, we counted the proportion of neural pairs (i, j) having a significantly non-null value for their cross-correlogram $\mathrm{CCG}_{ij}(\tau)$. [2] The typical correlation times range from 0 to a few tens of msec. Bair et al. 01 [1] find very similar time scales in area MT. On the other hand, they observe a larger proportion of the cells with significant correlations (around 70% of tested pairs, at time lag $\tau = 0$ msec).

[2]We deemed "significant" any value $|\mathrm{CCG}_{ij}(\tau)|$ exceeding $3\sigma_{ij}(\tau)$, where $\sigma^2_{ij}(\tau)$ is the expected variance for the measure $\mathrm{CCG}_{ij}(\tau)$ under the hypothesis that the two neurons are independent. If $\mathrm{CCG}_{ij}(\tau)$ is estimated from T (discrete, contiguous) time bins over N trial repetitions, and we assume stationary firing for the neurons, then one can show that

$$\sigma^2_{ij}(\tau) = \frac{1}{NT^2} \sum_{\Delta=-T}^{T} \mathrm{CCG}_{ii}(\Delta)\mathrm{CCG}_{jj}(\Delta)\big(T - |\Delta|\big).$$

As a result, $\sigma^2_{ij}(\tau)$ can be estimated experimentally from the neurons' auto-correlograms $\mathrm{CCG}_{ii}(\Delta)$ and $\mathrm{CCG}_{jj}(\Delta)$.

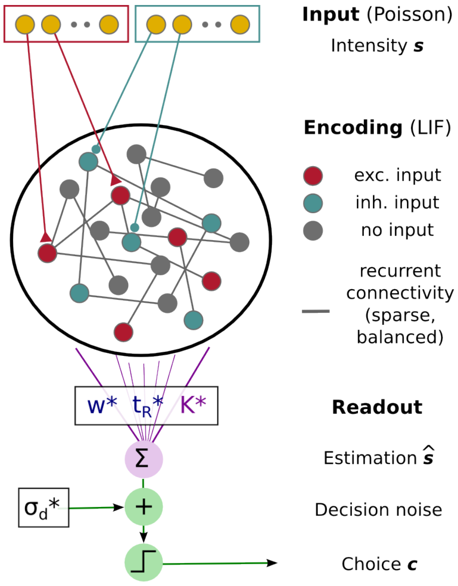

Figure 1: **Simulated neural network for testing the inference method** (see text for details).

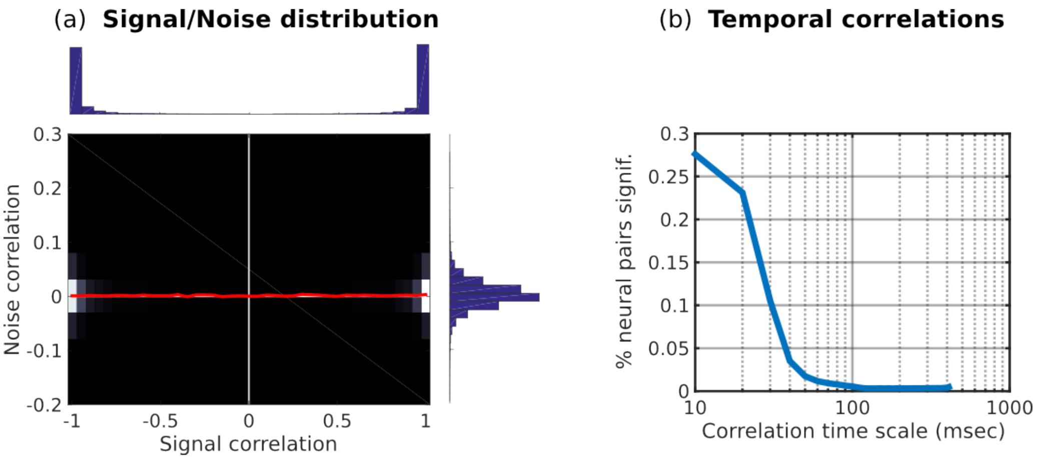

Figure 2: **Supplementary information about noise correlations in the network.** (a) Joint distribution of signal and noise correlations (for spike counts over 300 ms after stimulus onset). (b) Percentage of neural pairs with a significantly non-null value of the cross-correlogram $\mathrm{CCG}_{ij}(\tau)$, as a function of time lag τ. See the text for details.

4 Bayesian regularization of the optimal readout

The regularization procedure aims to counteract overfitting effects that may appear in Fisher's linear discriminant (main text, eq. 12) when the computation is based on too little data points. Our procedure is directly inspired by *Bayesian linear regression*, as exposed in Bishop 2006 [2], chapter 3. We refer the reader to this book for additional details.

We start with our notations—mostly similar to those introduced in the main text. We directly consider the time-integrated versions of the spike trains, with some fixed parameters w and t_R, and focus on some candidate readout ensemble $\mathcal{E}$ of size K. Then, our experimental data consist of the recorded spike counts $\bar{r}_{iq}$, where $i = 1 \dots K$ runs over neurons in $\mathcal{E}$, and $q = 1 \dots T$ represents the set of recorded trials. We will note $\bar{\mathbf{r}}_q$ for the vector of neural activities on trial q, and s_q for the stimulus value used on trial q.

We will generally note $\mathrm{E}[X] = T^{-1} \sum_{q=1}^{T} X_q$ for the (empirical) average of quantity X_q over trials. Without loss of generality, we make the offset $\bar{\mathbf{r}}_q \leftarrow \bar{\mathbf{r}}_q - \mathrm{E}[\bar{\mathbf{r}}]$ and $s_q \leftarrow s_q - \mathrm{E}[s]$. This will simplify the following formulas by removing cumbersome constant terms. As in the main text, we introduce the following (empirical) statistics:

$$\mathbf{A} := \mathrm{E}[\bar{\mathbf{r}}\bar{\mathbf{r}}^\top]$$
$$\sigma_s^2 := \mathrm{E}[s^2]$$
$$\bar{\mathbf{b}} := \sigma_s^{-2}\mathrm{E}[s\bar{\mathbf{r}}].$$

Matrix $\mathbf{A}$ is the total covariance matrix, $\bar{\mathbf{b}}$ is the tuning vector, and σ_s^2 is the variance of the tested stimuli. Note the simplified definitions as compared to the main text, because we now have $\mathrm{E}[\bar{\mathbf{r}}] = 0$ and $\mathrm{E}[s] = 0$.

The goal of Fisher's linear discriminant is to find a vector $\mathbf{a}$ maximizing the signal-to-noise ratio (SNR) of variable $\mathbf{a}^\top\bar{\mathbf{r}}$, with respect to the stimulus s. Under our assumption that the system depends linearly on s, the signal term writes $\sigma_s^2(\mathbf{a}^\top\bar{\mathbf{b}})^2$ (main text, eq. 51). Mathematically, the simplest option is to fix this signal term once and for all. Then, the SNR will simply be maximized by minimizing the total covariance, $\mathbf{a}^\top\mathbf{A}\mathbf{a}$ (main text, eq. 53). The logical choice for the signal term is to assume $\mathbf{a}^\top\bar{\mathbf{b}} = 1$ (unbiased percept). This imposes that vector $\mathbf{a}$ lie in a certain hyperplane, of dimension $K - 1$. We hard-code this constraint by the following reparametrization:

$$\mathbf{a} = \frac{\bar{\mathbf{b}}}{\|\bar{\mathbf{b}}\|^2} + \mathbf{M}\mathbf{c}, \tag{3}$$

where $\mathbf{M}$ is a $(K \times K - 1)$ matrix whose columns constitute an orthogonal basis of $\{\bar{\mathbf{b}}\}^\perp$, the orthogonal space of vector $\bar{\mathbf{b}}$. Vector $\mathbf{c} \in \mathbb{R}^{K-1}$ constitutes our reparametrization of vector $\mathbf{a}$.

At this point, we can make the link with classic linear regression. Indeed, the mean square error of $\mathbf{a}^\top\bar{\mathbf{r}}$ as

an estimator of stimulus s writes:

$$
\begin{aligned}
F_{\text{data}}(\mathbf{a}) &:= \sum_{q=1}^{T} (s_q - \mathbf{a}^\top \bar{\mathbf{r}}_q)^2 \\
&= \Big(\sum_{q=1}^{T} s_q^2 \Big) - 2\mathbf{a}^\top \Big(\sum_{q=1}^{T} s_q \bar{\mathbf{r}}_q \Big) + \mathbf{a}^\top \Big(\sum_{q=1}^{T} \bar{\mathbf{r}}_q \bar{\mathbf{r}}_q^\top \Big) \mathbf{a} \\
&= T \Big(\sigma_s^2 - 2\sigma_s^2 \, \mathbf{a}^\top \bar{\mathbf{b}} + \mathbf{a}^\top \mathbf{A} \mathbf{a} \Big).
\end{aligned}
$$

Having imposed $\mathbf{a}^\top \bar{\mathbf{b}} = 1$, we are left with $F_{\text{data}}(\mathbf{a}) = T(\mathbf{a}^\top \mathbf{A}\mathbf{a} - \sigma_s^2)$. In turn, $\mathbf{a}^\top \mathbf{A}\mathbf{a}$ is the total covariance. So ultimately, maximizing the SNR is equivalent to minimizing $F_{\text{data}}(\mathbf{a})$ under the constraint that $\mathbf{a}^\top \bar{\mathbf{b}} = 1$.

In Bayesian linear regression, one does not simply minimize $F_{\text{data}}(\mathbf{a})$, but uses it to derive a full distribution *a posteriori* for vector $\mathbf{a}$ given the data [2]. One first considers a (Gaussian) generative model for the data given $\mathbf{a}$, depending on a hyperparameter named β:

$$
\mathrm{P}(s_q | \mathbf{a}, \bar{\mathbf{r}}_q, \beta) = \Big(\frac{\beta}{2\pi} \Big)^{\frac{1}{2}} \exp\Big(-\beta \frac{(s_q - \mathbf{a}^\top \bar{\mathbf{r}}_q)^2}{2} \Big).
$$

On the other hand, one requires that the inferred vector $\mathbf{a}$ be *regular*: its entries a_i should not take too big values, because this is typically a mark of overfitting, see [2]. We thus impose a regularization prior on $\mathbf{a}$, depending on a hyperparameter named α:

$$
\mathrm{P}(\mathbf{a}|\alpha) = \Big(\frac{\alpha}{2\pi} \Big)^{\frac{K}{2}} \exp\Big(-\alpha \frac{\|\mathbf{a}\|^2}{2} \Big).
$$

This gives rise to the overall generative model for the data:

$$
\mathrm{P}(\{s_q\}_{q=1\ldots T}, \mathbf{c} | \alpha, \beta) = \Big(\frac{\beta}{2\pi} \Big)^{\frac{T}{2}} \Big(\frac{\alpha}{2\pi} \Big)^{\frac{K-1}{2}} \exp\Big(-\frac{\beta}{2} F_{\text{data}}\Big(\frac{\bar{\mathbf{b}}}{\|\bar{\mathbf{b}}\|^2} + \mathbf{M}\mathbf{c} \Big) - \frac{\alpha}{2} \|\mathbf{c}\|^2 \Big). \tag{4}
$$

Note the reparametrization of vector $\mathbf{a}$ with vector $\mathbf{c}$ (eq. 3), to account for the fact that $\mathbf{a}^\top \bar{\mathbf{b}} = 1$. Just as in classic Bayesian regression, the log-probability in eq. 4 is a quadratic function of vector $\mathbf{c}$.

The regularization procedure *per se* consists of finding the hyperparameters (α, β) which maximize the overall likelihood of the data, when marginalizing over the readout vector $\mathbf{c}$. In other words, we seek (α, β) that maximize the so-called *evidence function*:

$$
\mathrm{P}(\{s_q\}_{q=1\ldots T} | \alpha, \beta) = \int_{\mathbf{c} \in \mathbb{R}^{K-1}} \mathrm{P}(\{s_q\}_{q=1\ldots T}, \mathbf{c} | \alpha, \beta) d\mathbf{c}. \tag{5}
$$

This maximization is often referred to as *empirical Bayes*. It can be performed based on an EM algorithm, where $\mathbf{c}$ is the latent variable. We refer to [2] for details, and only provide here the algorithm. We note $\mathbf{c} \sim \mathcal{N}(\mathbf{m}_T, \mathbf{\Sigma}_T)$ for the posterior distribution of vector $\mathbf{c}$. In the E step, we deduce the values of $\mathbf{m}_T$ and $\mathbf{\Sigma}_T$

from eq. 4, assuming a fixed value of α and β:

$$\boldsymbol{\Sigma}_T = \left(\alpha\mathbf{Id} + \beta T\mathbf{M}^\top\mathbf{A}\mathbf{M}\right)^{-1},$$
$$\mathbf{m}_T = -\beta T\boldsymbol{\Sigma}_T\mathbf{M}^\top\mathbf{A}\frac{\bar{\mathbf{b}}}{\|\bar{\mathbf{b}}\|^2},$$
$$\mathbf{a}_T = \frac{\bar{\mathbf{b}}}{\|\bar{\mathbf{b}}\|^2} + \mathbf{M}\mathbf{m}_T.$$

(Vector $\mathbf{a}_T$ is directly obtained from $\mathbf{m}_T$ with eq. 3.) In the M step, we find the hyperparameters α and β which maximize the likelihood in eq. 4, marginalized over $\mathbf{c}$ assuming the distribution $\mathbf{c} \sim \mathcal{N}(\mathbf{m}_T, \boldsymbol{\Sigma}_T)$:

$$\alpha = \frac{K-1}{\|\mathbf{m}_T\|^2 + \mathbf{tr}(\boldsymbol{\Sigma}_T)},$$
$$\beta = \left(\mathbf{a}_T^\top\mathbf{A}\mathbf{a}_T + \mathbf{tr}(\mathbf{M}^\top\mathbf{A}\mathbf{M}\boldsymbol{\Sigma}_T)\right)^{-1}.$$

By iterating these two steps, the EM algorithm is guaranteed to converge to a (local) maximum for the evidence function in eq. 5.

At convergence, we obtain a solution to our original problem of regularization. The hyperparameters α and β implement the optimal trade-off between minimizing $\mathbf{a}^\top\mathbf{A}\mathbf{a}$ (maximizing the SNR), and minimizing $\|\mathbf{a}\|^2$ (regularization). This optimal trade-off will mostly depend on T, the number of trials entering the computation of $\mathbf{A}$ and $\bar{\mathbf{b}}$. The final vector $\mathbf{a}_T$ is the maximum a posteriori estimate for vector $\mathbf{a}$. A simple computation shows that $\mathbf{M}^\top(\beta T\mathbf{A} + \alpha\mathbf{Id})\mathbf{a}_T = 0$, meaning that $(\beta T\mathbf{A} + \alpha\mathbf{Id})\mathbf{a}_T$ is proportional to $\bar{\mathbf{b}}$. Hence, as stated in the main text, one has

$$\mathbf{a}_T = \frac{(\mathbf{A} + \lambda\mathbf{Id})^{-1}\bar{\mathbf{b}}}{\bar{\mathbf{b}}^\top(\mathbf{A} + \lambda\mathbf{Id})^{-1}\bar{\mathbf{b}}},$$

with the strength of regularization given by $\lambda = \alpha/(\beta T)$. (We have not investigated whether the optimal λ found by our procedure coincides with the one that would be found in classic Bayesian regularization, without imposing $\mathbf{a}^\top\bar{\mathbf{b}} = 1$. In general, they are probably of comparable magnitude.)

We define the corrected JND, Z, as the expected value for the noise variance $\mathbf{a}^\top\bar{\bar{\mathbf{C}}}\mathbf{a}$ under the posterior distribution for $\mathbf{a}$ (plus the potential decision noise, σ_d^2). One can show that it verifies : $Z^2 = \beta^{-1} - \sigma_s^2 + \sigma_d^2$ (see main text, eq. 53). Similarly, we define CC curves (main text, eq. 9) as the expected value for $\bar{\mathbf{C}}(t)\mathbf{a}$ under the posterior distribution for $\mathbf{a}$. By linearity, this is simply $\bar{\mathbf{C}}(t)\mathbf{a}_T$.

Naturally, Bayesian linear regression is just a model-based inference from the data, so it is not guaranteed to always counteract overfitting in the optimal fashion. In our empirical tests, the JNDs recovered after regularization were still somewhat smaller than their true values (as can be seen in the main text, Figure 5d-e), meaning that there was some residual overfitting. Nonetheless, the situation was much improved compared to when the correction was not applied, and we would strongly recommend to apply this procedure in data-scarce situations. As a drawback, the simple linear inversion required in the original formula for Fisher's LD (main text, eq. 12) is replaced by multiple iterations of the EM algorithm—which must be performed anew for every tested set of readout parameters. So the regularization procedure is quite time-costly.

5 Unbiased estimation of CC indicators q and V

When it comes to computing CC indicators $q(u,t)$ and V, the finite amount of data can bias the results in two ways. First, due to the *finite number of trials*, each neuron's individual statistic (say, $d_i^\star$) will differ from the "perfect" value that would be obtained from an infinite number of trials. Second, due to the *finite number of neurons*, each population-wide indicator (say, $\bar{\bar{q}}^\star =< \bar{b}_i \bar{d}_i^\star >_i$) will differ from the "perfect" value that would be obtained if all neurons in the population had been recorded.

Here, we present a generic description of this problem, which applies both to the "true" and "predicted" CC indicators from the main text, and we detail the specific corrections to ensure unbiasedness.

5.1 Nature of the problem, notations

We are given a neural population of interest, Ω, of cardinal M. Each neuron i in the population has two attributes b_i and d_i, and we wish to estimate the following population-wide indicators :

$$q := \langle b_i d_i \rangle_{i\in\Omega}\,, \tag{6}$$

$$B := \langle b_i^2 \rangle_{i\in\Omega}\,, \tag{7}$$

$$D := \langle d_i^2 \rangle_{i\in\Omega}\,, \tag{8}$$

$$V := BD - q^2\,. \tag{9}$$

The names are directly inspired from the main text, without the complications due to temporal dependencies, temporal averaging, stars, readout parameters, etc. For clarity, we also give explicit names to the two intermediate quantities B and D, involved in the computation of V.

Unfortunately, all the data are not available to perform these computations exactly, and we need to introduce some more notations. First, we will use the generic notation $\langle x_i \rangle_{i\in\mathcal{R}}$ to denote the population average of quantity x_i over neurons i in some fixed neural ensemble $\mathcal{R}$, possibly smaller than Ω. That is:

$$\langle x_i \rangle_{i\in\mathcal{R}} := \frac{1}{\mathrm{Card}(\mathcal{R})} \sum_{i\in\mathcal{R}} x_i.$$

In our case, $\mathcal{R}$ is a generic notation for any subset of the neurons that were actually recorded ; we note R for its cardinal. We assume that the choice of $\mathcal{R}$ amongst Ω is totally random and independent from everything else.

Second, due to the finite number of recording trials, we do not access the "perfect" values b_i and d_i, but experimental versions $\widehat{b}_i$ and $\widehat{d}_i$ which are corrupted by measurement noise. In our case, we may assume that $\widehat{b}_i$ and $\widehat{d}_i$ are unbiased estimators of their true values, meaning that

$$\mathrm{E}[\widehat{b}_i] = b_i,$$

$$\mathrm{E}[\widehat{d}_i] = d_i.$$

Here, the expectation refers to the random nature of the recording trials which lead to the measures of $\widehat{b}_i$ and $\widehat{d}_i$. In turn, the variances $\mathrm{Var}[\widehat{b}_i]$ and $\mathrm{Var}[\widehat{d}_i]$ give the strength of the respective measurement errors. For simplicity,

we assume that the measurement error on each $\widehat{b}_i$ or $\widehat{d}_i$ is independent from all other errors. There are several ways of estimating these errors in practice (e.g., based on a model). In this work, we estimated each quantity $\mathrm{Var}[\widehat{x}_i]$ from a bootstrap principle, as detailed below.

5.2 Unbiased estimators

Unbiased estimator for q. Given the above notations, our naive estimator for q in eq. 6 is:

$$\widehat{q} := \langle \widehat{b}_i \widehat{d}_i \rangle_{i\in\mathcal{R}}. \tag{10}$$

This estimator is directly suitable for our needs, because it is unbiased. Indeed:

$$\mathrm{E}\big[\widehat{q}\big] = \mathrm{E}\big[\frac{1}{R}\sum_{i\in\Omega} \mathbb{1}_i^{\mathcal{R}} \widehat{b}_i \widehat{d}_i\big] = \frac{1}{R}\sum_{i\in\Omega} \mathrm{E}\big[\mathbb{1}_i^{\mathcal{R}}\big]\mathrm{E}\big[\widehat{b}_i\big]\mathrm{E}\big[\widehat{d}_i\big] = \frac{1}{R}\sum_{i\in\Omega}\frac{R}{M} b_i d_i = q.$$

Conversely, the squaring operations involved in eq. 7-9 systematically transform measurement errors into positive biases. For example, $\widehat{q}^2$ is *not* an unbiased estimator of q^2, so we need to introduce specific corrections. The following lemma allows to derive all the corrections required below.

Lemma. *Let $\{x_i, y_i\}_{i\in\Omega}$ a set of values of interest over a population Ω, of cardinal M. Suppose that*

- *we access the values of x_i through estimators $\widehat{x}_i$ for $i \in \mathcal{S}$, a sub-ensemble of Ω of cardinal $S \leq M$,*
- *we access the values of y_i through estimators $\widehat{y}_i$ for $i \in \mathcal{R}$, a sub-ensemble of $\mathcal{S}$ of cardinal $R \leq S$,*

and assume the following properties for the various estimators:

$$\begin{aligned} \mathrm{E}(\widehat{x}_i) &= x_i && \textit{(unbiased estimator of } x_i\textit{)} \\ \mathrm{E}(\widehat{y}_i) &= y_i && \textit{(unbiased estimator of } y_i\textit{)} \\ \mathrm{Cov}(\widehat{x}_i, \widehat{y}_j) &= \delta_{ij} W_i && \textit{(possible element-wise variance)} \end{aligned}$$

Then, the following quantity:

$$\langle \widehat{x}_i \rangle_{i\in\mathcal{S}} \langle \widehat{y}_i \rangle_{i\in\mathcal{R}} \quad - \quad \underbrace{\frac{M-S}{(S-1)M}\Big(\langle \widehat{x}_i \widehat{y}_i \rangle_{i\in\mathcal{R}} - \langle \widehat{x}_i \rangle_{i\in\mathcal{S}} \langle \widehat{y}_i \rangle_{i\in\mathcal{R}}\Big)}_{\text{“X term”}} \quad - \quad \underbrace{\frac{1}{M}\langle W_i \rangle_{i\in\mathcal{R}}}_{\text{“W term”}}$$

provides an unbiased estimator of $\langle x_i \rangle_{i\in\Omega} \langle y_i \rangle_{i\in\Omega}$.

The proof is given at the end of this section. Rephrased, the sum of the “X” and “W” terms provides an unbiased estimator of the quantity $\mathrm{Cov}\big(\langle \widehat{x}_i \rangle_{i\in\mathcal{S}}, \langle \widehat{y}_i \rangle_{i\in\mathcal{R}}\big)$. On the one hand, the “X term” assesses the covariations of x_i and y_i from one neuron to the other. On the other hand, the “W term” will appear when $\widehat{x}_i = \widehat{y}_i$, so that $W_i = \mathrm{Var}[\widehat{x}_i]$, our measurement error on each x_i.

Unbiased estimator for q^2. In this case, we apply the lemma with $\mathcal{R} = \mathcal{S}$, $x_i = y_i = b_i d_i$, and their unbiased estimator $\widehat{x}_i = \widehat{y}_i = \widehat{b}_i \widehat{d}_i$, yielding the following unbiased estimator for q^2:

$$\widehat{[q^2]} := \widehat{q}^2 - \frac{M-R}{(R-1)M}\Big(\big\langle \widehat{b}_i^2 \widehat{d}_i^2 \big\rangle_{i\in\mathcal{R}} - \widehat{q}^2\Big) - \frac{1}{M}\big\langle \mathrm{Var}[\widehat{b}_i \widehat{d}_i]\big\rangle_{i\in\mathcal{R}}. \tag{11}$$

Unbiased estimator for BD. First, it is simple to obtain unbiased estimators for the intermediate quantities B and D in eq. 7-8. For example:

$$\mathrm{E}\big[\big\langle \widehat{b}_i^2 \big\rangle_{i\in\mathcal{S}}\big] = \big\langle b_i^2 \big\rangle_{i\in\Omega} + \mathrm{E}\big[\big\langle \mathrm{Var}[\widehat{b}_i]\big\rangle_{i\in\mathcal{S}}\big],$$

where the second term (average measurement error in the population) corresponds to the bias. A similar relationship holds for $\widehat{d}_i$. As a result:

$$\widehat{B} := \big\langle \widehat{b}_i^2 \big\rangle_{i\in\mathcal{S}} - \big\langle \mathrm{Var}[\widehat{b}_i]\big\rangle_{i\in\mathcal{S}} \quad \text{is an unbiased estimator of } B, \tag{12}$$

$$\widehat{D} := \big\langle \widehat{d}_i^2 \big\rangle_{i\in\mathcal{R}} - \big\langle \mathrm{Var}[\widehat{d}_i]\big\rangle_{i\in\mathcal{R}} \quad \text{is an unbiased estimator of } D. \tag{13}$$

(Note that the available population for the average can be different for $\widehat{B}$ and $\widehat{D}$.)

In turn, $\widehat{B}\widehat{D}$ is *not* an unbiased estimator of BD. To derive the corrections, we apply the lemma with $x_i = b_i^2$ measured over ensemble $\mathcal{S}$, and $y_i = d_i^2$ measured over ensemble $\mathcal{R}$ (assumed to be included in $\mathcal{S}$). Their respective unbiased estimators are $\widehat{x}_i = \widehat{b}_i^2 - \mathrm{Var}[\widehat{b}_i]$ and $\widehat{y}_i = \widehat{d}_i^2 - \mathrm{Var}[\widehat{d}_i]$ (and so $W_i = 0$). Furthermore, concerning the term $\widehat{x}_i \widehat{y}_i$ which appears in the lemma, we note that for every $i \in \mathcal{R}$:

$$\mathrm{E}\big[\widehat{x}_i \widehat{y}_i\big] = b_i^2 d_i^2 = \mathrm{E}\big[\widehat{b}_i^2 \widehat{d}_i^2\big] - \mathrm{Var}[\widehat{b}_i \widehat{d}_i],$$

so the leftmost term can be replaced by the rightmost term without modifying its expected value. We deduce the following unbiased estimator for BD:

$$\widehat{[BD]} := \widehat{B}\widehat{D} - \frac{M-S}{(S-1)M}\Big(\big\langle \widehat{b}_i^2 \widehat{d}_i^2 \big\rangle_{i\in\mathcal{R}} - \big\langle \mathrm{Var}[\widehat{b}_i \widehat{d}_i]\big\rangle_{i\in\mathcal{R}} - \widehat{B}\widehat{D}\Big). \tag{14}$$

5.3 Application : "true" and "predicted" indicators

Estimating measurement errors. We estimate all measurement errors thanks to a bootstrap principle. We generate T surrogate versions of our data, by resampling with replacement from the original trials. Then, each neuron's individual statistic (say, $\widehat{d}_i$) gives rise to T surrogate measures $\widehat{d}_i^{(rs)}$, and we estimate the measurement error on d_i as:

$$\mathrm{Var}[\widehat{d}_i] := \Big[\frac{1}{T}\sum_{rs=1}^{T} (\widehat{d}_i^{(rs)})^2\Big] - \widehat{d}_i^2. \tag{15}$$

For the large-scale simulation presented in the article, we use $T = 14$ resamplings. Furthermore, we use the *same* 14 resamplings to derive error bars on our final estimates (see main text). This departure from the

statistical canon was imposed by the length of the whole inference procedure. As a result, when correcting for measurement errors on a resampled set of data, we remove *twice* the estimated measurement error from eq. 15. For example, for D in eq. 13, our "unbiased estimator" on resamplings is

$$\widehat{D}^{(rs)} = \big\langle (\widehat{d}_i^{(rs)})^2 \big\rangle_{i\in\mathcal{R}} - 2\big\langle \mathrm{Var}[\widehat{d}_i] \big\rangle_{i\in\mathcal{R}}.$$

This allows for $\widehat{D}^{(rs)}$ to have the same expectation as the original $\widehat{D}$. Without this ad hoc procedure, the final CC indicators computed from the resamplings ($q^{(rs)}$ and $V^{(rs)}$) would be systematically biased compared to the original ones (q and V).

True indicators. Let $\mathcal{N}$ be the set of all recorded neurons, of cardinal N. For the "true" indicators, the individual neural data are $\widehat{b}_i$ and $\widehat{d}_i^\star$. The population of interest Ω is the full population under study, of cardinal $M = N_{\mathrm{tot}}$, while the available ensemble is $\mathcal{R} = \mathcal{S} = \mathcal{N}$. Then, eq. 10-14 lead to the following unbiased estimators:

$$\begin{aligned}
\widehat{q^\star} &= \big\langle \widehat{b}_i \widehat{d}_i^\star \big\rangle_{i\in\mathcal{N}} \\
\widehat{[q^\star]^2} &= \widehat{q^\star}^2 - \frac{N_{\mathrm{tot}} - N}{(N-1)N_{\mathrm{tot}}}\Big(\big\langle \widehat{b}_i^2 \widehat{d}_i^2 \big\rangle_{i\in\mathcal{N}} - \widehat{q}^2 \Big) - \frac{1}{N_{\mathrm{tot}}} \big\langle \mathrm{Var}[\widehat{b}_i \widehat{d}_i] \big\rangle_{i\in\mathcal{N}}. \\
\widehat{B} &= \big\langle \widehat{b}_i^2 - \mathrm{Var}\big[\widehat{b}_i\big] \big\rangle_{i\in\mathcal{N}} \\
\widehat{D^\star} &= \big\langle (\widehat{d}_i^\star)^2 - \mathrm{Var}\big[\widehat{d}_i^\star\big] \big\rangle_{i\in\mathcal{N}} \\
\widehat{[BD^\star]} &= \widehat{B}\widehat{D^\star} - \frac{N_{\mathrm{tot}} - N}{(N-1)N_{\mathrm{tot}}}\Big(\big\langle \widehat{b}_i^2 \widehat{d}_i^2 \big\rangle_{i\in\mathcal{N}} - \big\langle \mathrm{Var}[\widehat{b}_i \widehat{d}_i] \big\rangle_{i\in\mathcal{N}} - \widehat{B}\widehat{D^\star} \Big). \\
\widehat{V^\star} &= \frac{N(N_{\mathrm{tot}} - 1)}{(N-1)N_{\mathrm{tot}}}\Big(\widehat{B}\widehat{D^\star} - \widehat{q^\star}^2 \Big) + \frac{N_{\mathrm{tot}} - 1}{(N-1)N_{\mathrm{tot}}} \big\langle \mathrm{Var}[\widehat{b}_i \widehat{d}_i] \big\rangle_{i\in\mathcal{N}}.
\end{aligned}$$

The last line is directly obtained as $\widehat{V^\star} = \widehat{[BD^\star]} - \widehat{[q^\star]^2}$. As announced, specific corrections appear owing to the finite amounts of data. Concerning the *finite number of neurons*, the correction on the first term is akin to the famous "$N/(N-1)$" correction on a sample's empirical variance, in order to produce an unbiased estimator of the true variance. Concerning the *finite number of trials*, we need to correct all our formulas with the (bootstrap-estimated) measurement errors $\mathrm{Var}[\widehat{b}_i]$ (to compute $\widehat{B}$), $\mathrm{Var}[\widehat{d}_i]$ (to compute $\widehat{D}$) and $\mathrm{Var}[\widehat{b}_i \widehat{d}_i]$.

Predicted indicators. For the "predicted" versions, we face two additional difficulties. First, for each candidate readout ensemble $\mathcal{E}$, the corresponding CC indicators are obtained as the compound of *two* predictions:

- On the one hand, the prediction for CC signals *inside* the readout ensemble $\mathcal{E}$: $q_\mathcal{E} := \langle b_i d_i \rangle_{i\in\mathcal{E}}$.
- On the other hand, the prediction for CC signals *outside* the readout ensemble $\mathcal{E}$: $q_{\text{out}} := \langle b_i d_i \rangle_{i\notin\mathcal{E}}$.

Both predictions are then mixed with a weighting $p := K/N_{\text{tot}}$ (see main text), so that:

$$\begin{aligned} q &= p\, q_\mathcal{E} \;+\; (1-p)\, q_{\text{out}}\,, \\ V &= B\Big(p\, D_\mathcal{E} + (1-p)\, D_{\text{out}}\Big) - q^2\,. \end{aligned}$$

Second, the final CC indicators are obtained by averaging q and V over several candidate ensembles $\mathcal{E}$ (see main text). To ease the implementation of this final averaging, we always use the same estimator $\widehat{B}$ computed from all recorded neurons—independently of the candidate ensemble $\mathcal{E}$:

$$\widehat{B} = \big\langle \widehat{b}_i^2 - \text{Var}\big[\widehat{b}_i\big] \big\rangle_{i\in\mathcal{N}}.$$

For CC signals *inside* $\mathcal{E}$, we apply eq. 10-11 with $\Omega = \mathcal{R} = \mathcal{E}$, of cardinal $M = R = K$. Then we apply eq. 14 with $\mathcal{S} = \mathcal{N}$ and $\mathcal{R} = \mathcal{E}$. We thus obtain the following unbiased estimators:

$$\begin{aligned} \widehat{q_\mathcal{E}} &= \big\langle \widehat{b}_i \widehat{d}_i \big\rangle_{i\in\mathcal{E}} \\ \widehat{[q_\mathcal{E}^2]} &= \widehat{q_\mathcal{E}}^{\,2} - \frac{1}{K}\big\langle \text{Var}[\widehat{b}_i\widehat{d}_i]\big\rangle_{i\in\mathcal{E}} \\ \widehat{D_\mathcal{E}} &= \big\langle \widehat{d}_i^2 - \text{Var}\big[\widehat{d}_i\big]\big\rangle_{i\in\mathcal{E}} \\ \widehat{[BD_\mathcal{E}]} &= \widehat{B}\widehat{D_\mathcal{E}} - \frac{N_{\text{tot}} - N}{(N-1)N_{\text{tot}}}\Big(\big\langle \widehat{b}_i^2\widehat{d}_i^2\big\rangle_{i\in\mathcal{E}} - \big\langle \text{Var}[\widehat{b}_i\widehat{d}_i]\big\rangle_{i\in\mathcal{E}} - \widehat{B}\widehat{D_\mathcal{E}}\Big). \end{aligned}$$

For CC signals *outside* $\mathcal{E}$, the population of interest Ω has cardinal $M = N_{\text{tot}} - K$. We apply eq. 10-11 with $\mathcal{R} = \mathcal{I}$, the set of complimentary neurons, of cardinal $R = I$ (see main text). Then we apply eq. 14 with $\mathcal{S} = \mathcal{N}$ and $\mathcal{R} = \mathcal{I}$. We thus obtain the following unbiased estimators:

$$\begin{aligned} \widehat{q_{\text{out}}} &= \big\langle \widehat{b}_i \widehat{d}_i \big\rangle_{i\in\mathcal{I}} \\ \widehat{[q_{\text{out}}^2]} &= \widehat{q_{\text{out}}}^{\,2} - \frac{N_{\text{tot}} - K - I}{(I-1)(N_{\text{tot}} - K)}\Big(\big\langle \widehat{b}_i^2\widehat{d}_i^2\big\rangle_{i\in\mathcal{I}} - \widehat{q_{\text{out}}}^{\,2}\Big) - \frac{1}{N_{\text{tot}} - K}\big\langle \text{Var}[\widehat{b}_i\widehat{d}_i]\big\rangle_{i\in\mathcal{I}} \\ \widehat{D_{\text{out}}} &= \big\langle \widehat{d}_i^2 - \text{Var}\big[\widehat{d}_i\big]\big\rangle_{i\in\mathcal{I}} \\ \widehat{[BD_{\text{out}}]} &= \widehat{B}\widehat{D_{\text{out}}} - \frac{N_{\text{tot}} - N}{(N-1)N_{\text{tot}}}\Big(\big\langle \widehat{b}_i^2\widehat{d}_i^2\big\rangle_{i\in\mathcal{I}} - \big\langle \text{Var}[\widehat{b}_i\widehat{d}_i]\big\rangle_{i\in\mathcal{I}} - \widehat{B}\widehat{D_{\text{out}}}\Big) \end{aligned}$$

Finally, our unbiased compound estimators are:

$$\begin{aligned} \widehat{q} &= p\,\widehat{q_\mathcal{E}} + (1-p)\,\widehat{q_{\text{out}}}\,, \\ \widehat{V} &= p\,\widehat{[BD_\mathcal{E}]} \;+\; (1-p)\,\widehat{[BD_{\text{out}}]} \;-\; p^2\,\widehat{[q_\mathcal{E}^2]} \;-\; (1-p)^2\,\widehat{[q_{\text{out}}^2]} \;-\; 2p(1-p)\,\widehat{q_\mathcal{E}}\widehat{q_{\text{out}}}\,. \end{aligned}$$

These "predicted" estimators are generally not reliable, owing to the small size of complimentary ensemble $\mathcal{I}$. However, the final prediction is obtained as an average over a large number of pairs ($\mathcal{E}$, $\mathcal{I}$)—see main text. Thanks to the linear structure of $\widehat{q}$ and $\widehat{V}$, this averaging can be performed "online", without storing the results for each tested pair ($\mathcal{E}$, $\mathcal{I}$). Since each component is unbiased, the final average will also be unbiased, and reliable.

5.4 Proof of the lemma

The expectation of $\langle\widehat{x}_i\rangle_{i\in\mathcal{S}}\langle\widehat{y}_i\rangle_{i\in\mathcal{R}}$ writes as follows.

$$\begin{aligned}
\mathrm{E}\big[\langle\widehat{x}_i\rangle_{i\in\mathcal{S}}\langle\widehat{y}_i\rangle_{i\in\mathcal{R}}\big] &= \mathrm{E}\Big[\frac{1}{SR}\sum_{i,j\in\Omega}\mathbb{1}_i^{\mathcal{S}}\mathbb{1}_j^{\mathcal{R}}\widehat{x}_i\widehat{y}_j\Big] \\
&= \frac{1}{SR}\Big(\mathrm{E}\Big[\sum_{i\in\Omega}\mathbb{1}_i^{\mathcal{S}}\mathbb{1}_i^{\mathcal{R}}\widehat{x}_i\widehat{y}_i\Big] + \sum_{i\neq j}\mathrm{E}\big[\mathbb{1}_i^{\mathcal{S}}\mathbb{1}_j^{\mathcal{R}}\widehat{x}_i\widehat{y}_j\big]\Big) \\
&= \frac{1}{SM}\sum_{i\in\Omega}\mathrm{E}\big[\widehat{x}_i\widehat{y}_i\big] + \frac{S-1}{SM(M-1)}\sum_{i\neq j}\mathrm{E}\big[\widehat{x}_i\widehat{y}_j\big] \\
&= \frac{1}{SM}\sum_{i\in\Omega}[x_iy_i + W_i] + \frac{S-1}{SM(M-1)}\sum_{i\neq j}x_iy_j \\
&= \frac{(S-1)M}{S(M-1)}\langle x_i\rangle_{i\in\Omega}\langle y_i\rangle_{i\in\Omega} + \frac{M-S}{S(M-1)}\langle x_iy_i\rangle_{i\in\Omega} + \frac{1}{S}\langle W_i\rangle_{i\in\Omega}
\end{aligned}$$

In the third line, we note that $\mathcal{R}\subset\mathcal{S}$, so that $\mathrm{E}\big[\mathbb{1}_i^{\mathcal{S}}\mathbb{1}_i^{\mathcal{R}}\big] = \frac{R}{M}$ and, when $i\neq j$, $\mathrm{E}\big[\mathbb{1}_i^{\mathcal{S}}\mathbb{1}_j^{\mathcal{R}}\big] = \frac{R(S-1)}{M(M-1)}$.
In the last line, we note that $\sum_{i\neq j}x_iy_j = (\sum_\Omega x_i)(\sum_\Omega y_j) - \sum_\Omega x_iy_i$, and we collect all terms of interest with their respective weightings.

The last two terms in the above sum can be replaced by their unbiased estimators. Namely :

$$\begin{aligned}
\langle W_i\rangle_{i\in\mathcal{R}} &\quad \text{is an unbiased estimator of } \langle W_i\rangle_{i\in\Omega}\,, \\
\langle\widehat{x}_i\widehat{y}_i - W_i\rangle_{i\in\mathcal{R}} &\quad \text{is an unbiased estimator of } \langle x_iy_i\rangle_{i\in\Omega}\,.
\end{aligned}$$

Then, multiplying the above relationship by $\frac{S(M-1)}{(S-1)M}$ and collecting terms again, we find that

$$\mathrm{E}\left[\frac{S(M-1)}{(S-1)M}\langle\widehat{x}_i\rangle_{i\in\mathcal{S}}\langle\widehat{y}_i\rangle_{i\in\mathcal{R}} - \frac{M-S}{(S-1)M}\langle\widehat{x}_i\widehat{y}_i\rangle_{i\in\mathcal{R}} - \frac{1}{M}\langle W_i\rangle_{i\in\mathcal{R}}\right] = \langle x_i\rangle_{i\in\Omega}\langle y_i\rangle_{i\in\Omega}.$$

The final result of the lemma is a rearranged form of this equation, making more explicit the corrective nature of term "X".

6 Extended model with non-deterministic extraction time t_R

We detail here the analytical modifications when the model's extraction time t_R is non-deterministic. We thus assume that t_R is itself a random variable, drawn on each trial according to some density function $g(t)$, independently of neural activities $\mathbf{r}(t)$. (Note that this is a restrictive hypothesis. In particular, it rules out a direct application of the model to an integration-to-bounds framework.) The full readout model is then given by:

$$\begin{aligned} t_R &\sim g(t), \\ \widehat{s} &= \sum_i \int_{u>0} a_i r_i(t_R - u) h_w(u) du. \end{aligned}$$

For concision, we have noted the integration kernel at scale w: $h_w(u) := w^{-1}h(u/w)$. This model naturally encompasses the simpler version presented in the main text. To obtain a deterministic readout time t_R, we simply need to set $g(t) = \delta(t - t_R)$ where $\delta(\cdot)$ corresponds to the Dirac delta-function.

Derivation. For any deterministic function of time $x(t)$, we note $\mathrm{E}_R(x) := \mathrm{E}(x(t_R)) = \int_{t=-\infty}^{+\infty} g(t)x(t)dt$, and $\mathrm{Var}_R(x) := \mathrm{E}_R(x^2) - \mathrm{E}_R(x)^2$. Then, for any random process $X(t)$ constructed from the spike trains (and hence, independent of t_R), we have:

$$\begin{aligned} \mathrm{E}(X(t_R)) &= \mathrm{E}_R\big(\mathrm{E}(X)\big), \\ \mathrm{Var}(X(t_R)) &= \mathrm{E}_R\big(\mathrm{Var}(X)\big) + \mathrm{Var}_R\big(\mathrm{E}(X)\big), \end{aligned}$$

this last line stemming from the law of total variance. These two formulas allow to compute the moments of $\widehat{s}$ given s, in a fashion similar to the main text (Methods, eq. 35-37). After these computations, we find that the general form of the characteristic equations still holds:

$$\begin{aligned} \partial_s \mathrm{E}[\widehat{s}|s] &= \bar{\mathbf{b}}^\top \mathbf{a}\,, \\ \mathrm{Var}[\widehat{s}|s] &= \mathbf{a}^\top \mathbf{\Gamma} \mathbf{a}\,, \\ \mathrm{Cov}[\mathbf{r}(t), \widehat{s}|s] &= \bar{\mathbf{C}}(t)\mathbf{a}\,, \end{aligned}$$

but with more general definitions for $\bar{\mathbf{b}}$, $\bar{\mathbf{C}}(t)$ and $\mathbf{\Gamma}$:

$$\bar{b}_i := \int_u (g \star h_w)(u) b_i(u)\, du, \tag{16}$$

$$\bar{C}_{ij}(t) := \int_u (g \star h_w)(u) C_{ij}(t, u)\, du, \tag{17}$$

$$\Gamma_{ij} := \iint_{(u,v)} G_w(u, v) C_{ij}(u, v)\, du\, dv + V_{ij}^{temp}, \tag{18}$$

having defined $g \star h_w(u) = \int_t g(t)h_w(t - u)dt$ and $G_w(u, v) := \int_t g(t)h_w(t - u)h_w(t - v)dt$.

Interpretation. Through eq. 17, $g(t)$ acts a weighting factor over the CC curves that would be obtained for each t_R: $\bar{\mathbf{C}}(t|g) = \int_u g(u)\bar{\mathbf{C}}(t|t_R = u)du$. This leads to the spreading of CC curves sketched in main text, Fig 8a. Furthermore, matrix V_{ij}^{temp} is an additional source of variance that appears only when $g(t)$ has an extended temporal support, i.e., when t_R is non-deterministic:

$$V_{ij}^{temp} := \iint_{(u,v)} G_w(u,v)\mathrm{E}\Big[\Big(m_i(u;s) - \overline{m_i}(s)\Big)\Big(m_j(v;s) - \overline{m_j}(s)\Big)\Big]\, du\, dv,$$

where $\overline{m_i}(s) := \int_t (g\star h_w)(t) m_i(t;s)dt$ is the corresponding temporal average for $m_i(t;s)$. Thus, V_{ij}^{temp} measures a form of temporal covariance in the PSTHs for the neurons[3]. Indeed, if t_R varies from trial to trial, any variation of firing rates in time creates an additional source of variability in $\widehat{s}$.

Extension of our method. Could the statistical approach introduced in the main text be extended, to recover a non-deterministic extraction function $g(t)$? One possible concern is that the temporal evolution of CC signals is only determined by the aggregate function $(g \star h_w)(t)$ (eq. 17), which cannot be used to disentangle $g(t)$ and w separately.

However, note that in the sensitivity equation (eq. 18), one now has $\bar{\bar{\mathbf{C}}} \neq \mathbf{\Gamma}$ in general—as opposed to the case with deterministic t_R. Instead, the respective effects of $g(t)$ and w on $\bar{\mathbf{C}}(t)$ and $\mathbf{\Gamma}$ (eq. 17-18) can roughly be thought of as a scaling:

$$\bar{\bar{\mathbf{C}}} \simeq \rho(w,g)\mathbf{\Gamma}, \tag{19}$$

because the overall "shape" of covariance between neurons (as opposed to its "strength") does not depend much on the precise temporal integration used to compute their activity. For example, eq. 19 is exactly verified if (1) neural activities are stationary and (2) the profile of temporal correlation is homogeneous across neurons: $C_{ij}(t,u) = \bar{\bar{C}}_{ij} F(|t-u|)$. Precisely, in this case,

$$\rho(w,g) = \frac{\int_\xi \widetilde{F}(\xi)\|\widetilde{h_w}(\xi)\|^2 \|\widetilde{g}(\xi)\|^2 d\xi}{\int_\xi \widetilde{F}(\xi)\|\widetilde{h_w}(\xi)\|^2 d\xi}$$

expressed in terms of Fourier transforms.

As a result, the CC indicator $q(u,t)$ (main text, eq. 14) is predicted to scale as $\rho(w,g)$. So, while matching the temporal support of $\langle q\rangle_{\mathcal{E}}(u,t)$ and $q^\star(u,t)$ constrains the value of $(g\star h_w)(t)$, matching their overall amplitude constrains $\rho(w,g)$. Thus, we may hope that minimizing the loss term $\iint_{u,t}(\langle q\rangle_{\mathcal{E}}(u,t) - q^\star(u,t))^2 dudt$ will allow to disentangle the values of $g(t)$ and w separately. In practice though, this would require the fitting of at least one additional temporal parameter ; typically, the standard deviation of t_R from trial to trial.

[3] The average $\mathrm{E}[\cdot]$, running over the different tested stimuli s, is only here to produce a stimulus-independent covariance structure, in keeping with the main text. In the general case, it is possible that $V_{ij}(s)$ have an explicit dependency on the tested stimulus s.

References

[1] W. Bair, E. Zohary, and W. T. Newsome. Correlated firing in macaque visual area MT: time scales and relationship to behavior. *Journal of Neuroscience*, 21(5):1676–97, 2001.

[2] C. M. Bishop. *Pattern recognition and machine learning*. Springer Verlag, New York, USA, 2006.

[3] D.J. Daley and D. Vere-Jones. *An introduction to the theory of point processes*, volume 1. Springer Verlag, New York, USA, 2007.

[4] D. Goodman and R. Brette. Brian: a simulator for spiking neural networks in python. *Frontiers in neuroinformatics*, 2, 2008.

[5] D.M. Green and J.A. Swets. *Signal detection theory and psychophysics*, volume 1974. Wiley, New York, USA, 1966.

[6] R. M. Haefner, S. Gerwinn, J. H. Macke, and M. Bethge. Inferring decoding strategies from choice probabilities in the presence of correlated variability. *Nature Neuroscience*, 16(2):235–242, 2013.

[7] A. Wohrer, M. D. Humphries, and C. K. Machens. Population-wide distributions of neural activity during perceptual decision-making. *Progress in Neurobiology*, 103:156–193, 2013.
