# Supplementary material for: On the Number of Neurons and Time Scale of Integration Underlying the Formation of Percepts in the Brain
Source: PLoS Comput Biol. 2015 Mar 20;11(3):e1004082. doi: 10.1371/journal.pcbi.1004082 (PMC4368836; doi:10.1371/journal.pcbi.1004082)
Supplement: S1 Compressed file archive — (GZ) [file pcbi.1004082.s002.gz › WohrerMachens14_code/Instructions.pdf]

On the number of neurons and time-scale of integration underlying the formation of percepts in the brain

A. Wohrer and C. Machens, 2014

This archive contains Matlab code implementing the inference method, presented as `Case 2' of the article. The Matlab functions are designed to be general, so they can be applied to any data from a 2AFC task, with a minimal amount of modifications. The overall usage is described in Section I of this document.

For a deeper look, each of the main functions is thoroughly documented through a dedicated page in folder **doc/html/**, which can be opened with any browser. For a faster look at the overall architecture of the Matlab code, you can type **help matlab_code** under Matlab. You can similarly explore the two specific subdirectories : **help lif_simulation** and **help tools**. Finally, the code of most functions is carefully commented.

Remark : In order for Matlab to know the location of the functions, directory **matlab_code/** and its subfolders should be included in your Matlab PATH. The simplest way to do this is to execute the Matlab script **startup.m** contained in the archive's main directory (this is done automatically if you start Matlab in this directory).

This archive also provides *data* on which to test the inference method, as exposed in the article : that is, a network of recurrent LIF neurons encoding a simple form of stimulus. Two options are available in this regard.

1) The results of a default LIF simulation are already included in the archive, inside folder **DATA/lif_simulation_1/**. This corresponds to a medium-sized network, with 500 coupled LIF neurons. This is the data on which the inference method is applied, by default.

2) Python code is included, to produce other types of LIF simulations with the same overall architecture. See Section II of this document for explanations.

In the article, the inference method is illustrated on a large-scale network with 5000 coupled LIF neurons. The raw data for this experiment is too large to be included in the archive – so the user has to regenerate it, using the Python functions. The parameters for this simulation, and for the subsequent analysis in Matlab, are stored inside folder **article_simulation/**. Instructions are also provided to reproduce all figures from the article.

WARNING : the whole simulation + analysis with the parameters of the article can take up to a few days. We recommend to start with the shorter analysis proposed by default.

I – Inference method

Open Matlab in the archive's main directory, and open the script **launch_analysis_lif.m**. You will find a list of parameter definitions and function calls implementing the full inference method. The LIF data used by default are those provided in **DATA/lif_simulation_1/**.

Launching the script succcessively performs the 4 stages detailed below.

Important remark : The first stage is only required when applying the inference method to the synthetic LIF simulations of the article. To apply the inference method on another set of data (e.g., true experimental data), you should directly start at section (2) [if the data are from a 2AFC task with a single scalar stimulus and fixed threshold], or at section (3) [other types of perceptual decision making tasks, to be worked out on a case-by-case basis].

1) Transform the LIF simulation into a full 2AFC experiment

First, we create a perceptual readout from the simulated spikes. The goal of the inference method will precisely be to *recover* the parameters of this readout.

Second, we implement the interface between the data produced by the Python simulations, and the raw data format which is used for subsequent processing in Matlab.

The output of this stage is a file **DATA/lif_simulation_1/1_expe.mat**, which provides Matlab with all the information required to access the raw experimental data in the task (spikes + `animal' percept on every trial).

The main Matlab function in charge of this part is called **simul_build_experiment**. All related Matlab functions are stored in subfolder **matlab_code/lif_simulation**.

2) Compute the basic statistics for individual neurons

Namely : PSTHs, JPSTHs, Choice covariance curves, and the psychometric curve.

These quantities serve as basic material for the subsequent inference method. We compute, not only the empirical statistics from the raw data, but also a number of ***trial-resampled*** versions, which will ultimately allow to derive confidence intervals.

The output of this stage is a directory **DATA/lif_simulation_1/1_stats/**, which contains all the required measures under a stringent format (see the **doc/html/** documentation for more details).

The main Matlab function in charge of this part is called **compute_individual_statistics**. To visualize the computed statistics, use function **visualize_statistics**.

3) Compute the indicators Z, q(u,t) and V, used in the inference method

On the one hand, we compute the `true' versions $Z^*$, $q^*$ and $V^*$, directly measured from the data. On the other hand, we compute the `predicted' versions Z, q and V, based on the *restricted optimality* assumption, for different sets of readout parameters (w ,t_R, K, σ_d). The output of this part is a file **DATA/lif_simulation_1/1_predictions.mat**, storing all the true values and predictions for the indicators.

The main Matlab function in charge of this part is called **compute_predictions.**
It implements all the core computations of the inference method. It can be quite long (up to several days). In case of a program interruption, it is possible to relaunch it without losing (too much of) the stuff already computed. See the **doc/html/** documentation for the function (bottom remarks).

4) Infer the most plausible readout parameters (w^*, t_R^*, K^*, σ_d^*)

This is done by seeking for the best match between the `true' and `predicted' versions of the indicators. We finally represent the results of the inference.

The main Matlab function in charge of this part is called **infer_readout_scales**.

II – Generate synthetic LIF data

In order to test the method on another set of synthetic data than the one proposed by default, we include some code to simulate a network of LIF neurons with intrinsic tuning to some input stimulus, as used in the article. The code is in Python, based on the neural network simulator Brian. So **make sure to have Python+Brian installed** before going any further !

Then, you should make sure that Python can locate our functions, no matter where you call it from. Under Unix, open a Bash terminal in the archive's main directory and type

```
export PYTHONPATH=${PYTHONPATH}:$PWD/python_code/
```

(Under Windows or with another shell than Bash, you must find for yourself the appropriate equivalent command :p).

1) Define all network parameters

All parameters of the LIF network are defined in a script called **define_network.py**. Typing

```
python  python_code/define_network.py
```

will read this script, and create the network accordingly. We refer to the article (supplementary material) for a detailed description of the network implementation.

By default, the network parameters correspond to the LIF simulation already included in the archive, with 500 neurons. Modify them to create differently parametrized networks. By default, the network parameters are stored in :

DATA/lif_simulation_2/network_definition.mpd.

Here, **.mpd** is a homemade format (standing for `*Matlab Python Data*') which allows a simple sharing of data between Matlab and Python.

2) Simulate the LIF network

With the command

```
python  python_code/launch_network.py
```

This creates a number of files containing all the raw spike trains, saved as **DATA/lif_simulation_2/simulations/file_[x].mpd**, where **[x]** encodes a number of repetitions of the experiment (50 by default). Again, all parameters can be changed by editing the script. (For example : location of the file **network_definition.mpd**, nature of the stimulus, number of repetitions, etc.)

NOTA : This can be quite long (up to several days in case of large networks !). In case of a program interruption, you can relaunch the script **launch_network.py** from where it stopped. Open the script and change the first number in variable **simRange**, in order to skip all simulation files that have already been created. Then relaunch the network simulation.
