# Supplementary material for: On the Number of Neurons and Time Scale of Integration Underlying the Formation of Percepts in the Brain
Source: PLoS Comput Biol. 2015 Mar 20;11(3):e1004082. doi: 10.1371/journal.pcbi.1004082 (PMC4368836; doi:10.1371/journal.pcbi.1004082)
Supplement: S1 Compressed file archive — (GZ) [file pcbi.1004082.s002.gz › WohrerMachens14_code/doc/html/simul_build_experiment.html]

simul\_build\_experiment 

# simul\_build\_experiment

Produce "full" experimental data from our synthetic LIF network simulation. Starting from the spikes produced by the LIF simulation (in Python), this function:

1) Adds a "perceptual report" to the simulation:

```
      a) Define all readout parameters (readout ensemble, w, tR, sigD, etc.)
      b) Build the report on each trial
```

2) Saves all experimental data (spikes + report) under a "standard" format, which will be readable by function compute\_individual\_statistics.

## Contents

- Usage
- Required contents of the baseDir
- For more details

## Usage

**simul\_build\_experiment(baseDir, expeFile, report, data)**

- baseDir (string) : base directory for the experiment.
- expeFile (string) : name of the output file (inside baseDir). The content of this file (loaded as a Matlab structure) will provide the input structure "expe" to function compute\_individual\_statistics.
- report : Matlab structure with all parameters required to define/compute the perceptual report, on the basis of network activity.
- data : Matlab structure defining the location, and contextual parameters, of the raw experimental data.

## Required contents of the baseDir

When launching this function, the baseDir should contain the following files, produced by the LIF simulation (in Python) :

- The 'network\_definition.mpd' file defining all parameters of the LIF network
- A subdirectory (or symbolic link) named 'simulations/', containing all output files from the simulation ('file\_x.mpd').

## For more details

check out the comments inside the function's code.

Published with MATLAB® R2013b
