# Supplementary material for: On the Number of Neurons and Time Scale of Integration Underlying the Formation of Percepts in the Brain
Source: PLoS Comput Biol. 2015 Mar 20;11(3):e1004082. doi: 10.1371/journal.pcbi.1004082 (PMC4368836; doi:10.1371/journal.pcbi.1004082)
Supplement: S1 Compressed file archive — (GZ) [file pcbi.1004082.s002.gz › WohrerMachens14_code/doc/html/visualize_statistics.html]

visualize\_statistics 

# visualize\_statistics

Visualize all "individual statistics" for the neurons, as saved inside a statDir architecture.

## Contents

- Usage
- Remark

## Usage

**visualize\_statistics(baseDir, statDir, Tchar, doExtras)**

- baseDir (string) : base directory for the experiment.
- statDir (string) : sub-directory of baseDir containing all the individual neuron statistics.
- Tchar (float) : characteristic time constant for (Gaussian) smoothing of the curves. (Tchar = 0 produces unbiased, but noisy, statistics).
- doExtras (boolean, default true) : also plot the PSTHs for the neurons, and psychometric curve for the animal. (Strictly speaking, these statistics are not a requirement of function compute\_predictions.)

## Remark

- If doExtras = true, this function looks for PSTHs under the name 'r\_x', and for psychometric curves under the name 'psy\_x', where x is the number of the experimental run (the statDir architecture is described here).

Published with MATLAB® R2013b
