# Supplementary material for: On the Number of Neurons and Time Scale of Integration Underlying the Formation of Percepts in the Brain
Source: PLoS Comput Biol. 2015 Mar 20;11(3):e1004082. doi: 10.1371/journal.pcbi.1004082 (PMC4368836; doi:10.1371/journal.pcbi.1004082)
Supplement: S1 Compressed file archive — (GZ) [file pcbi.1004082.s002.gz › WohrerMachens14_code/doc/html/kappafun_2AFC.html]

kappafun\_2AFC 

# kappafun\_2AFC

Compute the ratio kappa(Z), from Percept Covariance to Choice Covariance, in the same context as in the article : 2AFC task with a single, scalar stimulus value and a fixed threshold.

```
function kappa = kappafun_2AFC(Z, mu, includedStims, stimProb)

kappa = sum( stimProb .* normpdf_(includedStims, mu, Z) );

end
```

Published with MATLAB® R2013b
