# Supplementary material for: On the Number of Neurons and Time Scale of Integration Underlying the Formation of Percepts in the Brain
Source: PLoS Comput Biol. 2015 Mar 20;11(3):e1004082. doi: 10.1371/journal.pcbi.1004082 (PMC4368836; doi:10.1371/journal.pcbi.1004082)
Supplement: S1 Compressed file archive — (GZ) [file pcbi.1004082.s002.gz › WohrerMachens14_code/doc/html/compute_individual_statistics.html]

compute\_individual\_statistics 

# compute\_individual\_statistics

Compute the "individual statistics" for the recorded neurons (tuning signals, JPSTHs, CC curves), from raw experimental data. These individual statistics are the basic data required by the inference method in compute\_predictions.

- The context is the same as in the article: a 2AFC discrimination task with a single scalar stimulus and a fixed threshold.
- The raw experimental data must accessible by the program in "standard" format, as detailed below.
- The individual neuron statistics are saved in a statDir architecture, which can be read by function compute\_predictions.

## Contents

- Usage
- Output
- General structure of the experimental task
- Contents of input structure expe
- "Standard" access to the raw data
- Contents of input structure stat
- Remarks

## Usage

**compute\_individual\_statistics(baseDir, statDir, expe, stat, compVersion)**

- baseDir (string) : base directory for the experiment.
- statDir (string) : sub-directory of baseDir where all individual neuron statistics will be saved.
- expe : Matlab structure providing access to the raw data (see sections below).
- stat : Matlab structure providing the additional parameters required to compute the statistics (see final section).
- compVersion : optional argument defining the way of parsing the data (see comments inside the code).

## Output

A statDir architecture, as detailed here.

## General structure of the experimental task

- The experimental task considered comprises a finite number of ***experimental conditions***, that is, a set of trials which are similar from the point of view of the experimenter, and over which it is natural to perform averaging.
- On each trial, the animal makes a particular ***report***. The programs herein are tuned to the classic 2AFC task, where the report is binary (extensions to other types of report would only require relatively little changes).
- The recordings are segregated into a number of experimental ***runs***, i.e., a set of trials during which the same neurons were recorded. Typically, a run would correspond to one day's worth of neural recordings, before the electrodes are removed until the next session.
- For generality, we allow the data from a single run to be segregated into a number of ***subfiles***, e.g. for memory issues.

## Contents of input structure expe

Input structure expe provides all contextual informations about the experiment, and how to access the raw data. It must define all the following fields:

|  |  |
| --- | --- |
| expe.type | '2AFC' is the only choice so far! |
 expe.nCond | Number of experimental conditions in the data set. | expe.stimVals | Vector of size (nCond, 1), providing the stimulus value used in each experimental condition.  (Note that two different conditions may provide the same stimulus value, although they are not equivalent with respect to some other aspect of the task.) | expe.nRuns | Number of experimental runs in the data set. | expe.nFiles | Vector of size (nRuns, 1), giving the number of subfiles associated to each run. | expe.nN | Vector of size (nRuns, 1), giving the number of neurons (simultaneously) recorded in each of the runs.  (Ideally, each run should provide the same number of neurons, to ensure that all neurons in the experiment are statistically equivalent.) | expe.get\_raw | Handle to the external function in charge of providing "standard" access to the raw data, as described below. | expe.grargs | Cell array containing all the contextual arguments required by function expe.get\_raw.  We will then access the raw data with the generic call:  [ spikelist , condlist , choicelist ] = expe.get\_raw( nrun , nfile , expe.grargs{:} ) |

## "Standard" access to the raw data

The raw experimental data consist of the neural spike trains and animal report, on each experimental trial.

Since experimental data can be stored in several ways, we do not impose a specific storage for this raw data. Instead, we simply require that the user provide *access* to the raw data, by writing an external function with the following signature:

[spikelist , condlist , choicelist ] = my\_get\_raw( nrun , nfile , arg1 , arg2 , ...)

- Function name: my\_get\_raw is just an example. The user can choose any name, as long as they specify the function handle in structure expe accordingly. For example, here, they would define: expe.get\_raw = @my\_get\_raw.
- For the synthetic LIF simulations presented in the article, the "standard" access function is called simul\_get\_raw.
- Required input: The two first integers indicate the experimental run, and subfile, from which the data must be extracted.
- Remaining inputs : depend on the user's specific requirements for the my\_get\_raw function. For example, they could indicate the directory where the measures are stored, some categorical information regarding the experiment in question, etc.
- Required function ouput :

|  |  |
| --- | --- |
| spikelist | Matrix of size (nSpikes, 3), containing all recorded spikes in the file, across all trials and neurons (so nSpikes is generally a large number).  **Each line of spikelist must take the form [time neuron trial]**.  The first number indicates the time of the spike, in seconds, relative to some reference time « 0 » in the trial.  The second number is the index of the firing neuron.  The third number is the index of the trial where the spike occured. |
 condlist | Vector of size (nTrials, 1), specifying the experimental condition that was used on each trial.  For example, with 5 possible conditions : condlist = [2 5 5 3 1 4, etc ]. | choicelist | Vector of size (nTrials, 1), specifying the animal's final choice on each trial.  For example, with a binary choice : choicelist = [0 1 1 0 0 1 , etc ]. |

## Contents of input structure stat

Input structure stat provides additional parameters required to compute the statistics. It must define the following fields :

|  |  |
| --- | --- |
| stat.axT | Discretized time axis, as a set of linear time bins : *axT = Tmin : Tbin : Tmax.*  The time bins must be expressed in seconds, relative to the reference time « 0 » on each trial (which typically corresponds to stimulus onset).  In the arrays that will be constructed to store the individual neuron statistics, all temporal dimensions will have size *nT := length(axT)*. |
 stat.nBoot | Number of resamplings created. See the doc of **compute\_predictions** for more details. | stat.includedConds | Boolean vector of size (expe.nCond, 1), precising which experimental conditions should be used to compute neural tuning (b), JPSTH (C) and Choice Cov (d).  Leave empty for default behavior : include all conditions (as in the article). |

## Remarks

- This function also computes additional quantities which are not directly required by function compute\_predictions. Namely, the PSTH for each neuron, and the psychometric curve for the animal in each run.
- All the statistics computed by this function can be visualized thanks to function visualize\_statistics.
- In the case of the synthetic LIF simulations from the article, structure expe is created by function simul\_build\_experiment.
- **Special usage:** To create additional resamplings in a preexisting statDir , pass as input the structure stat contained in statDir/0\_header.mat, but set stat.nBoot to the larger, desired number of resamplings. This will create the supplementary files [n].mat inside statDir (see here).
- The inference method can be applied, with minimal modifications, to other types of experiments. For example, the 2-stimulus discrimination task of Romo and colleagues. In this case, function **compute\_individual\_statistics** cannot be used directly to create the statDir architecture. However, much of its contents could be recycled to write an equivalent function for the new type of task.

Published with MATLAB® R2013b
