# Supplementary material for: On the Number of Neurons and Time Scale of Integration Underlying the Formation of Percepts in the Brain
Source: PLoS Comput Biol. 2015 Mar 20;11(3):e1004082. doi: 10.1371/journal.pcbi.1004082 (PMC4368836; doi:10.1371/journal.pcbi.1004082)
Supplement: S1 Compressed file archive — (GZ) [file pcbi.1004082.s002.gz › WohrerMachens14_code/doc/html/compute_predictions.html]

compute\_predictions 

# compute\_predictions

Core computations of the inference method presented in the article ("Case 2"). All lengthy (up to several days!) computations are performed here, while the final details and visualization of the results are performed by function infer\_readout\_scales.

## Contents

- Usage
- Output
- Method parameters
- The statDir architecture
- Remarks, examples

## Usage

**compute\_predictions(baseDir, statDir, outFile, method)**

- baseDir (string) : base directory for the experiment.
- statDir (string) : sub-directory of baseDir containing all the individual neuron statistics, used as basis material by this function. For a standard 2AFC task, the whole content of statDir can be created by function compute\_individual\_statistics, and you do not need to know its architecture in details. Else, see this section for a detailed description.
- outFile (string) : name of the output file (which will be saved in baseDir).
- method : Matlab structure defining the parameters of the inference method (see further sections).

## Output

Essentially, this function computes the three indicators used for the inference, namely, *Z*, *q(u,t)*, and *V*. On the one hand, it computes the "true" (or "starred") versions of the indicators, directly on the data. On the other hand, it computes the "predicted" versions of the indicators, depending on readout parameters , , , and .

In detail, the quantities stored in the outFile are not directly *Z*, *q(u,t)* and *V*, but "building blocks" that can easily be recombined to obtain the three indicators (see comments in the code for details).

## Method parameters

Input structure method specifies (almost) all parameters of the inference method. It must define the following fields :

|  |  |
| --- | --- |
| method.K\_vals | Vector of size (1, nKs). All tested values for parameter *K* (number of readout neurons). |
 method.w\_vals | Vector of size (1, nws). All tested values for parameter *w* (integration window, in seconds). | method.tR\_vals | Vector of size (1, ntRs). All tested values for parameter *t\_R* (readout time, in seconds). | method.sigd\_vals | Vector of size (1, nsigds). All tested values for parameter *\sigma\_d* (decision noise, in stimulus units). | method.nEs | Vector of size (1, nKs). Number of tested candidate ensembles, for each tested ensemble size *K*. | method.I\_vals | Number of ''external neurons'' (I) associated to each candidate ensemble (E).  (Reminder : these ''external'' neurons are used to estimate the strength of choice signals outside the readout ensemble.) | method.kernelType | String. Shape of the temporal averaging kernel. 'Window' or 'Exponential'. | method.doBayes | Boolean. If *true*, apply our Bayesian regularization scheme, when estimating the optimal readout vector for each ensemble E (supplementary information of the article). This is recommended when the data come from relatively few recording trials (in practice : all the time). However, it slows down the whole method.  If *false*, classic ridge regularization can still be used instead... but it requires to choose an arbitrary strength of regularization. | method.bayesStopCrit | Stopping criterion in the EM algorithm for Bayes regularization (only used if method.doBayes == true).  (relative change in one step for parameters alpha and beta). | method.bayesBootTrick | Trick to gain some time during bootstrap repetitions.  (use parameters alpha and beta from the original data). | method.EPSI | Classic regularization of Fisher's LDA (only used if method.doBayes == false).  (EPSI>0 => ridge regularization. EPSI=0 => pseudoinverse.) | method.nBoot | Optional, to enforce a smaller number of resamplings than in the *statDir*. |

## The statDir architecture

If required by the user (e.g., to apply the inference method to another task than 2AFC), they can provide manually the "individual neuron statistics" required by the method. That is, the user must provide the following quantities for all recorded neurons in the experiment:

- The *tuning signal* () for each recorded neuron.
- The *Joint PSTH* () for each pair of simultaneously recorded neurons.
- The *choice covariance curve* () for each recorded neuron.
- The psychometric *just-noticeable-difference* () for the animal.

Each quantity is explicitly defined in the article. Once computed, these quantities should be stored under a stringent format inside a subfolder of baseDir, that we generally refer to as the [statDir].

First, a *header* file should be available, with the name [statDir]/0\_header.mat. It should contain the following variables, which summarize all the contextual information required by the inference method :

|  |  |
| --- | --- |
| nRuns | Number of experimental runs in the data set. |
 nN | Vector of size (nRuns, 1), giving the number of neurons (simultaneously) recorded in each of the runs.  (Ideally, each run should provide the same number of neurons, to ensure that all neurons in the experiment are statistically equivalent.) | axT | Discretized time axis, as a set of linear time bins : *axT = Tmin : Tbin : Tmax.*  The time bins must be expressed in seconds, relative to the reference time « 0 » on each trial (which typically corresponds to stimulus onset).  In the arrays that store the individual neuron statistics, all temporal dimensions must have size *nT := length(axT)*. | nBoot | Number of resamplings created. Each "individual neuron statistic" is stored under *nBoot* copies on the hard drive, where each copy corresponds to a resampling of the original trials.  In turn, function **compute\_predictions** will compute *nBoot* copies of the indicators *Z*, *q(u,t)*, *V*. Ultimately, this allows to obtain error bars on the indicators, and also to correct indicator *V* for the finite number of recording trials. | nTrials | Vector of size (nRuns, 1), providing the *total* number of trials (across all experimental conditions) used to compute the statistics for each neuron. This information is required to calibrate the Bayesian regularization procedure used by function **compute\_predictions**.  For generality, this number is allowed to vary between the different experimental runs. However, large variations from one run to the other are not encouraged. | kappafun | Handle to an external function in charge of computing function *\kappa(Z)* from the article, i.e., the conversion factor from *Percept Covariance* to *Choice Covariance* as a function of psychometric sensitivity. | kfargs | Cell array containing all the contextual arguments required by function kappafun.  Inside function **compute\_predictions**, we will then compute factor *kappa* with the generic call:  kappa = kappafun( Z , kfargs{:} ) |

Second, [statDir] must contain the measured statistics of activity for all neurons in the experiment. These statistics are stored in nBoot distinct mat files – with one different file storing the statistics for each resampling. (This is for memory issues.)

The name of each file is simply the bootstrap number : [statDir]/1.mat contains the original statistics, and then 2.mat, ... , until [nBoot].mat contain the resamplings.

In each mat file, the required statistics are stored, with the following names and sizes :

- Z (1) : Just-noticeable-difference for the animal.
- b\_x (nN , nT) : tuning signal for each neuron (in experimental run x).
- C\_x (nN, nT, nN, nT) : JPSTH for each pair of neurons (in experimental run x).
- d\_x (nN, nT) : Choice covariance signal for each neuron (in experimental run x).

A different array must exist for each experimental run, and the number of the run is appended at the end of the variable. Again, this is for a more efficient handling of memory.

## Remarks, examples

- Function compute\_individual\_statistics allows to create the statDir and its contents automatically, assuming the same experimental context as in the article : a 2AFC discrimination task with a single scalar stimulus and a fixed threshold. The conversion factor « kappa(Z) » in this context is implemented by function kappafun\_2AFC, using the same definition of Choice Covariance as in the article (weighted mean across all tested stimuli).
- In other experimental contexts (for example, the two-frequency somatosensory discrimination task of Romo and colleagues), other definitions may be required for function kappa – which is why its precise definition is left to the user in general.
- Illustration of the statDir architecture. Here is how we would access the JPSTH between neurons 7 and 103 in experimental run number 8, for the third bootstrap resampling :

```
        statmat = matfile( [baseDir,'/',statDir,'/3'] ) ;
        jpsth = squeeze( statmat.C_8 ( 7 , : , 103, : ) ;
```

- **Special usage:** Suppose that outFile contains less resamplings than the statDir (either because function **compute\_predictions** was interrupted before finishing, either because additional resamplings have been added inside the statDir afterwards). Simply pass method=[] as input. This will reload all information from the outFile, and compute the additional resamplings.

Published with MATLAB® R2013b
