# Supplementary material for: On the Number of Neurons and Time Scale of Integration Underlying the Formation of Percepts in the Brain
Source: PLoS Comput Biol. 2015 Mar 20;11(3):e1004082. doi: 10.1371/journal.pcbi.1004082 (PMC4368836; doi:10.1371/journal.pcbi.1004082)
Supplement: S1 Compressed file archive — (GZ) [file pcbi.1004082.s002.gz › WohrerMachens14_code/doc/html/infer_readout_scales.html]

infer\_readout\_scales 

# infer\_readout\_scales

Visualization part of the inference method presented in the article ("Case 2"). This function must be called after the core computations have been peformed with function compute\_predictions.

## Contents

- Usage
- Additional parameters for the inference
- Contents of resFile
- Visualization of the results

## Usage

**infer\_readout\_scales(baseDir, predFile, resFile, infer)**

- baseDir (string) : base directory for the experiment.
- predFile (string) : result file produced by function compute\_predictions. (It must exist in baseDir.)
- resFile (string) : output file for this function.
- infer : Matlab structure defining additional parameters of the inference method (see below). As a special usage, set infer=[] to reload and display data from a preexisting resFile.

## Additional parameters for the inference

The "main" parameters of the method are defined when calling function **compute\_predictions**. Here, structure infer defines additional parameters which allow to compare the "true" and "predicted" indicators (*Z*, *q(u,t)* and *V*), in order to estimate the most plausible readout parameters , , , and .

The following parameters finalize the computation of indicators *Z*, *q(u,v)* and *V*:

|  |  |
| --- | --- |
| infer.Ntot | Integer: Full population size *Ntot*. This number is used to weight in the correct proportions the predicted choice signals within (ensemble E) and outside (ensemble I) the readout ensemble.  You may assume knowledge of the value (as in the article), or set it to *Inf* (more realistic, assumes that *K << Ntot*). |
 infer.bootcorrec\_V | Boolean: proceed to bootstrap-based correction on V ?  Very recommended in case of realistic recordings with relatively few trials. | infer.tSmooth | Float: Characteristic time scale of the (Gaussian) temporal smoothing on indicator *q(u,t)*, in seconds.  It allows to decrease measurement noise in case of realistic recordings with relatively few trials. Leave empty for no smoothing. |

The following parameters specify the loss function which allows to compare "true" and "predicted" indicators. Each weight is expressed relative to (the inverse of) the corresponding indicator's "true" value.

|  |  |
| --- | --- |
| infer.weight\_Z | Relative weight of indicator \_Z\_ in the loss function (default value: 1) |
 infer.weight\_q | Relative weight of indicator \_q\_ in the loss function (default value: 1) | infer.weight\_V | Relative weight of indicator \_V\_ in the loss function (default value: 1) |

The following parameters allow to perform a "pseudo-Bayesian" inference of the readout parameters (not shown in the article).

|  |  |
| --- | --- |
| infer.cost2ll | Conversion ratio from the cost function to a log-likelihood. |
 infer.prior.XX | Various options to define the prior over the readout parameters. See comments in the code. |

Additional, optional parameters can be found in the comments of the function. They are about details of the inference method, not really interesting but left for reference.

## Contents of resFile

The data are stored in the general format:

```
      XX(nb).[star/pred]([dimP dimX]) ,
```

where XX is one of the following names: Z / q / V / Q / correcV, star indicates the "true" measures and pred the predictions, and nb runs from 1 to nBoot. Finally, dimP and dimX are array dimensions which take the following values :

```
      dimP = [nKs nsigds nws ntRs] (for pred), [] (for star)
      dimX = 1 (for Z, V, Q), nT^2 (for q)
```

- Q is our notation for the (w,tR)-average of q (noted  in the article). More generally, in the code, a capital letter indicates (w,tR)-averaging of the corresponding, lower-case quantity.
- As an exception to the above formats, one has : Q(nb).star(1 1 nws ntRs)
- 'correcV' is the bootstrap-based correction over *V*, to account for the finite number of recording trials (the correction method is detailed in the article).

## Visualization of the results

The main visualization tool is a GUI, which allows to display all the computed values for the indicators (except q(u,t), see below).

- It can either display the *true* value of the indicator, or its *predicted* value as a function of the tested readout parameters. It can also display the *MSE* between true and predicted value, or the associated *likelihood*.
- You can choose the bounds of representation, and display the results for any of the tested resamplings (1 being the original samples).
- Amongst the 4 tested parameters, two are chosen to vary along the X and Y axis.
- The other 2 parameters are fixed, with one of the following methods:

|  |  |
| --- | --- |
| "Max likelihood" | Pick the "best" parameters, i.e., the ones corresponding to the argmin of the MSE function. |
 "Marginalize" | Marginalize across all the possible values for the parameters, using the final ('posterior') likelihood function. [This is kind of ad hoc!] | "Manual value" | Enter manually a specific value, in the form of two indices. [Pick them inside the correct bounds!] |

Two additional GUIs are also proposed:

1. Represent indicator *q(u,t)*, both in its true ("starred") and predicted versions. [The overall MSE between the two can be represented in the main GUI].
2. Represent the final set of parameters achieving the minimum of the MSE function. The actual estimator is represented as a red dot. The resampled estimators are represented as gray dots, and their covariance structure provides a confidence interval (in red) around the actual estimator.

All GUIs used for visualization are stored in matlab\_code/visualization.

Published with MATLAB® R2014b
