# Supplementary material for: On the Number of Neurons and Time Scale of Integration Underlying the Formation of Percepts in the Brain
Source: PLoS Comput Biol. 2015 Mar 20;11(3):e1004082. doi: 10.1371/journal.pcbi.1004082 (PMC4368836; doi:10.1371/journal.pcbi.1004082)
Supplement: S1 Compressed file archive — (GZ) [file pcbi.1004082.s002.gz › WohrerMachens14_code/doc/html/simul_get_raw.html]

simul\_get\_raw 

# simul\_get\_raw

"Standard access" function for the raw data in our simulated LIF network.

## Contents

- Usage
- Code

## Usage

```
function [spikelist, condlist, choicelist] = simul_get_raw(nrun, nfile, baseDir, data, allreports)
```

- nrun (int) : experimental run for which the data should be retrieved.
- nfile (int) : subfile (of the run) for which the data should be retrieved.
- baseDir (string) : base directory for the experiment.
- data (structure) : data parameters of the LIF simulation.
- allreports (array) : all reports for the "animal", as created by function simul\_build\_experiment.

For more details, check out the main function simul\_build\_experiment, which implements the "link" between the LIF simulations and the "standard" format of the data. (In particular, the commented code in "Part III" of the function).

## Code

The code is left for reference, as it is rather short, and vaguely informative:

```
% By convention, a "trial" consists of a transition "Stim1 -> Stim2", with
% time 0 corresponding to the transition, negative times ( > -stepT ) to
% stimulus 1, and positive times ( < stepT ) to stimulus 2.

% Retrieve the data for this file...

[simdata, sim] = simul_retrieve(baseDir, data.fileIDs_meas(nrun, nfile) );

nIsteps = sim.nIsteps ;
nRepeat = sim.nRepeat ;
stepT = sim.stepT ;

allspikes = simdata.spikes{2}( data.iTot(:,nrun) ) ;
nN = length(allspikes);
clear simdata

% ... and output it in the "standard" format:

% (I) spikelist(nSpikes, 3)

nSpikes = sum(cellfun(@length, allspikes));
spikelist = zeros(2*nSpikes, 3);

c = 0 ;                                         % spike count
for ni = 1:nN
    stp = floor(allspikes{ni}/stepT);           % which stimulus step (starts at 0)
    for contrib = 1:2
        if contrib == 1                         % Spikes' contribution to their own time step
            goodstp = stp>0;                    % (discard step 0 [burn in] as positive contributor)
            trial = stp(goodstp);

        else                                    % Spikes' contribution to the following time step
            goodstp = stp<nIsteps*nRepeat;      % (discard last step as negative contributor)
            trial = stp(goodstp)+1;
        end
        nkept = length(trial);                  % number of spikes kept for this neuron+contrib
        spikelist(c+(1:nkept), 1) = ...         % time inside relevant step
            allspikes{ni}(goodstp) - stepT*trial ;
        spikelist(c+(1:nkept), 2) = ni ;        % neuron index
        spikelist(c+(1:nkept), 3) = trial ;     % trial
        c = c + nkept ;
    end
end
spikelist = spikelist(1:c, :);                 % cut the few leftovers


% (II) condlist(trial)
condlist = repmat( (1:nIsteps)' , [nRepeat 1]) ;


% (III) choicelist(trial)
choicelist = reshape( allreports(:,:,nrun,nfile), [],1) ;
```

Published with MATLAB® R2013b
