# Supplementary figures and images for: On the Number of Neurons and Time Scale of Integration Underlying the Formation of Percepts in the Brain
Source: PLoS Comput Biol. 2015 Mar 20;11(3):e1004082. doi: 10.1371/journal.pcbi.1004082 (PMC4368836; doi:10.1371/journal.pcbi.1004082)

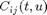

Supplement: S1 Compressed file archive — (GZ) [file pcbi.1004082.s002.gz › WohrerMachens14_code/doc/html/compute_predictions_eq31209.png]

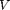

Supplement: S1 Compressed file archive — (GZ) [file pcbi.1004082.s002.gz › WohrerMachens14_code/doc/html/compute_predictions_eq20021.png]

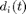

Supplement: S1 Compressed file archive — (GZ) [file pcbi.1004082.s002.gz › WohrerMachens14_code/doc/html/compute_predictions_eq43749.png]

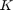

Supplement: S1 Compressed file archive — (GZ) [file pcbi.1004082.s002.gz › WohrerMachens14_code/doc/html/infer_readout_scales_eq03845174387838694102.png]

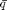

Supplement: S1 Compressed file archive — (GZ) [file pcbi.1004082.s002.gz › WohrerMachens14_code/doc/html/infer_readout_scales_eq20715.png]

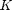

Supplement: S1 Compressed file archive — (GZ) [file pcbi.1004082.s002.gz › WohrerMachens14_code/doc/html/compute_predictions_eq14888.png]

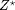

Supplement: S1 Compressed file archive — (GZ) [file pcbi.1004082.s002.gz › WohrerMachens14_code/doc/html/compute_predictions_eq29160.png]

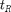

Supplement: S1 Compressed file archive — (GZ) [file pcbi.1004082.s002.gz › WohrerMachens14_code/doc/html/compute_predictions_eq07961.png]

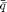

Supplement: S1 Compressed file archive — (GZ) [file pcbi.1004082.s002.gz › WohrerMachens14_code/doc/html/infer_readout_scales_eq43520.png]

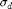

Supplement: S1 Compressed file archive — (GZ) [file pcbi.1004082.s002.gz › WohrerMachens14_code/doc/html/compute_predictions_eq32774.png]

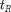

Supplement: S1 Compressed file archive — (GZ) [file pcbi.1004082.s002.gz › WohrerMachens14_code/doc/html/infer_readout_scales_eq07961.png]

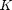

Supplement: S1 Compressed file archive — (GZ) [file pcbi.1004082.s002.gz › WohrerMachens14_code/doc/html/infer_readout_scales_eq14888.png]

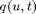

Supplement: S1 Compressed file archive — (GZ) [file pcbi.1004082.s002.gz › WohrerMachens14_code/doc/html/compute_predictions_eq40992.png]

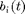

Supplement: S1 Compressed file archive — (GZ) [file pcbi.1004082.s002.gz › WohrerMachens14_code/doc/html/compute_predictions_eq52908.png]

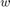

Supplement: S1 Compressed file archive — (GZ) [file pcbi.1004082.s002.gz › WohrerMachens14_code/doc/html/infer_readout_scales_eq00125694759345388081.png]

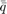

Supplement: S1 Compressed file archive — (GZ) [file pcbi.1004082.s002.gz › WohrerMachens14_code/doc/html/infer_readout_scales_eq01337433555260353225.png]

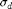

Supplement: S1 Compressed file archive — (GZ) [file pcbi.1004082.s002.gz › WohrerMachens14_code/doc/html/infer_readout_scales_eq32774.png]

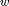

Supplement: S1 Compressed file archive — (GZ) [file pcbi.1004082.s002.gz › WohrerMachens14_code/doc/html/infer_readout_scales_eq64535.png]

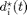

Supplement: S1 Compressed file archive — (GZ) [file pcbi.1004082.s002.gz › WohrerMachens14_code/doc/html/compute_predictions_eq11516.png]

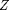

Supplement: S1 Compressed file archive — (GZ) [file pcbi.1004082.s002.gz › WohrerMachens14_code/doc/html/compute_predictions_eq14833.png]

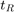

Supplement: S1 Compressed file archive — (GZ) [file pcbi.1004082.s002.gz › WohrerMachens14_code/doc/html/infer_readout_scales_eq14736097699691072511.png]

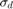

Supplement: S1 Compressed file archive — (GZ) [file pcbi.1004082.s002.gz › WohrerMachens14_code/doc/html/infer_readout_scales_eq10452312776744969231.png]

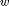

Supplement: S1 Compressed file archive — (GZ) [file pcbi.1004082.s002.gz › WohrerMachens14_code/doc/html/compute_predictions_eq64535.png]
